# Comparative effects of different types of cardioplegia in cardiac surgery: A network meta-analysis

**DOI:** 10.3389/fcvm.2022.996744

**Published:** 2022-09-13

**Authors:** Jia Tan, Siwei Bi, Jingyi Li, Jun Gu, Yishun Wang, Jiyue Xiong, Xiang Yu, Lei Du

**Affiliations:** ^1^Department of Anesthesiology, West China Hospital, Sichuan University, Chengdu, China; ^2^Department of Burn and Plastic Surgery, West China Hospital, Sichuan University, Chengdu, China; ^3^West China School of Medicine, Sichuan University, Chengdu, China; ^4^Department of Cardiovascular Surgery, West China Hospital, Sichuan University, Chengdu, China

**Keywords:** cardiac surgery, cardiac protection, blood cardioplegia, del Nido, HTK, St. Thomas, network meta-analysis

## Abstract

**Objective:**

To compare the outcomes of four types of cardioplegia during cardiac surgery: del Nido (DN), blood cardioplegia (BC), histidine-tryptophan-ketoglutarate (HTK) and St. Thomas.

**Methods:**

Randomized controlled trials (RCTs) and observational cohort studies from 2005 to 2021 were identified in PubMed, Embase, and Cochrane databases. Data were extracted for the primary endpoint of perioperative mortality as well as the following secondary endpoints: atrial fibrillation, renal failure, stroke, use of an intra-aortic balloon pump, re-exploration, intensive care unit stay and hospital stay. A network meta-analysis comparing all four types of cardioplegia was performed, as well as direct meta-analysis comparing pairs of cardioplegia types.

**Results:**

Data were extracted from 18 RCTs and 49 observational cohort studies involving 18,191 adult patients (55 studies) and 1,634 children (12 studies). Among adult patients, risk of mortality was significantly higher for HTK (1.89, 95% CI 1.10, 3.52) and BC (RR 1.73, 95% CI 1.22, 2.79) than for DN. Risk of atrial fibrillation was significantly higher for BC (RR 1.41, 95% CI 1.09, 1.86) and DN (RR 1.51, 95% CI 1.15, 2.03) than for HTK. Among pediatric patients, no significant differences in endpoints were observed among the four types of cardioplegia.

**Conclusions:**

This network meta-analysis suggests that among adult patients undergoing cardiac surgery, DN may be associated with lower perioperative mortality than HTK or BC, while risk of atrial fibrillation may be lower with HTK than with BC or DN.

## Introduction

During cardiac surgery, cardiac arrest is typically induced using cardioplegia under cardiopulmonary bypass (CPB) in order to ensure a bloodless surgical field and to protect the myocardium (1). Inadequate cardiac protection can increase the risk of perioperative mortality, atrial fibrillation, renal failure, stroke, use of an intra-aortic balloon pump (IABP), and extended stay in the intensive care unit (ICU) or hospital more generally. Several types of cardioplegia have been routinely performed since the 1950s (2). Common types of cardioplegia involving only crystalloid materials include the St. Thomas type and the histidine-tryptophan-ketoglutarate (HTK) type (3). The St. Thomas type involves an extracellular solution (4), while HTK involves an intracellular solution. HTK is widely used in cardiac and transplant surgeries to preserve organs (5).

Two other types of cardioplegia involve mixtures of crystalloids and blood products. The original blood cardioplegia (BC) is usually performed using a 1:4 mixture of the two. Del Nido (DN) cardioplegia, involving a 4:1 mixture, has recently entered clinical use (6). First used for pediatric cardiac surgery, it is now frequently used also for adult cardiac surgery (7).

Pairs of cardioplegia types have been compared in several clinical trials, but the results for a given pair have not always been consistent, and some techniques have not yet been compared head-to-head. Here we conducted a network meta-analysis (NMA) to evaluate the safety and efficacy of the four types of cardioplegia, based on direct and indirect evidence (8). We hope to provide clinical evidence to guide the choice of cardioplegia type.

## Methods

This NMA was performed according to the appropriate PRISMA extension (9) and MOOSE guidelines (10).

### Study selection and exclusion

We developed strategies for searching the literature and performing meta-analysis based on the following PICOS criteria: *patients*, adults or children undergoing cardiac surgery; *intervention and comparator*, any comparison involving DN, BC, HTK and/or St. Thomas types of cardioplegia; *outcomes*, perioperative mortality (defined as in-hospital or 30-day mortality) as the primary outcome, as well as atrial fibrillation, renal failure, stroke, use of an IABP, re-exploration, ICU stay, and hospital stay as secondary outcomes; *studies*, randomized controlled trials (RCTs) or observational cohort studies.

Thus, to be included in the meta-analysis, studies had to be RCTs or observational cohort studies of patients undergoing cardiac surgery involving at least one of the four types of cardioplegia: DN, BC, HTK or St. Thomas. The endpoint of each outcome depended on the longest follow-up in each study.

Studies were excluded from our review if they were conducted on animals or *in vitro*, if we did not have access to the full text, or if the study sample overlapped with a sample in a more recent study. We also excluded letters, commentaries, conference proceedings and trial protocols.

### Search strategy

Literature in the PubMed, Embase and Cochrane databases was systematically searched using the following search terms: cardiac surgery, del Nido, HTK solution, St. Thomas' solution, blood cardioplegia, randomized controlled trial and cohort study (Supplementary Tables 1–3). The range of possible publication dates was limited to 01 January 2005 through 31 December 2021. Publication language was limited to English. Disagreements about study inclusion were resolved through discussion; if necessary, a senior reviewer (J.G.) was consulted.

### Data extraction

Data were extracted from the selected studies by three reviewers (Y.-S. Wang, J.-G. Xiong, X.Y.) using a standard collection form consistent with the guidelines of the Cochrane Collaboration for Systematic Reviews (11). The following data were extracted: the first author of the study, publication year, country, intervention, sex, sample size, mean age, median cross time, median CPB time, temperature of cardioplegia solution, redosing interval, route of delivery, types of cardiac surgery, perioperative mortality, atrial fibrillation, renal failure, stroke, IABP, re-exploration, ICU stay, and hospital stay. Discrepancies in the data extracted by the three reviewers were resolved through discussion and, if necessary, consultation with a senior investigator (L.D.). Missing standard deviations were estimated from interquartile ranges according to the guidelines of the Cochrane Collaboration for Systematic Reviews (11, 12).

### Quality assessment

The methodological quality of RCTs was evaluated based on the Cochrane risk of bias tool (11), which assesses the following items: (1) random sequence generation, (2) allocation sequence concealment, (3) blinding of participants and personnel, (4) blinding of the outcome assessment, (5) incomplete outcome data, (6) selective reporting, and (7) other biases. The quality of observational cohort studies was assessed using the Newcastle-Ottawa Scale (NOS) (13).

### NMA of cardioplegia types

The transitivity assumption (14) was evaluated by comparing the distribution of the following potential effect modifiers across studies: publication frequency, median cross time, median CPB time, sex, and mean age. Means were compared in a pairwise fashion using Student's *t* test and across general groups using analysis of variance (ANOVA). *P* < 0.05 was considered significant. Pooled results were reported in terms of the mean difference (MD) for continuous outcomes or risk ratio (RR) for dichotomous outcomes, together with the corresponding 95% confidence intervals (CIs).

A random-effect model was used for the NMA. For each outcome, a network plot was established, in which each node represented an intervention and the size of nodes and thickness of connections between them reflected the number of studies involved in the comparison. The results were shown in a league plot. Interventions were ranked according to their surface under the curve cumulative ranking probabilities (SUCRA) (15). Higher score in the ranking meant greater likelihood that the given treatment would produce the greatest benefit.

### Subgroup analysis

The NMA was repeated for all outcomes using data either only from RCTs or only from cohort studies.

### Pairwise meta-analysis

Direct pairwise meta-analysis of cardioplegia types was conducted, and the results were reported in terms of RR and MD, together with the corresponding 95% CIs. Heterogeneity of pooled data was assessed using Cochrane's Q test and the I^2^ statistic (16). *P* > 0.1 in the Q test and *I*^2^ = 0% were taken to indicate absence of heterogeneity. Data were meta-analyzed using the Mantel–Haenszel fixed-effect model when *P* > 0.1 and *I*^2^ <50%; otherwise, they were analyzed using a random-effect model.

### Network consistency

The consistency between direct and indirect evidence was evaluated using node-splitting analysis, in which *P* < 0.05 was considered to indicate inconsistency (17). For comparisons involving at least 10 studies, comparison-adjusted funnel plots were generated and visually inspected: plots that were symmetrical along the midline were considered to indicate the absence of low-quality studies with small samples (14). The Egger regression test was conducted to assess the asymmetry of the funnel plot, with *P* > 0.05 taken to indicate no significant publication bias.

All analyses and plots were generated in R 3.4.0 (https://www.r-project.org). The following R packages were used: “gemtc” (18), “rjags” (19), “dmetar” (20), “ggplot2” (21), “BUGSnet” (22) and “netmeta” (23).

## Results

### Selected studies

**Figure 1:**
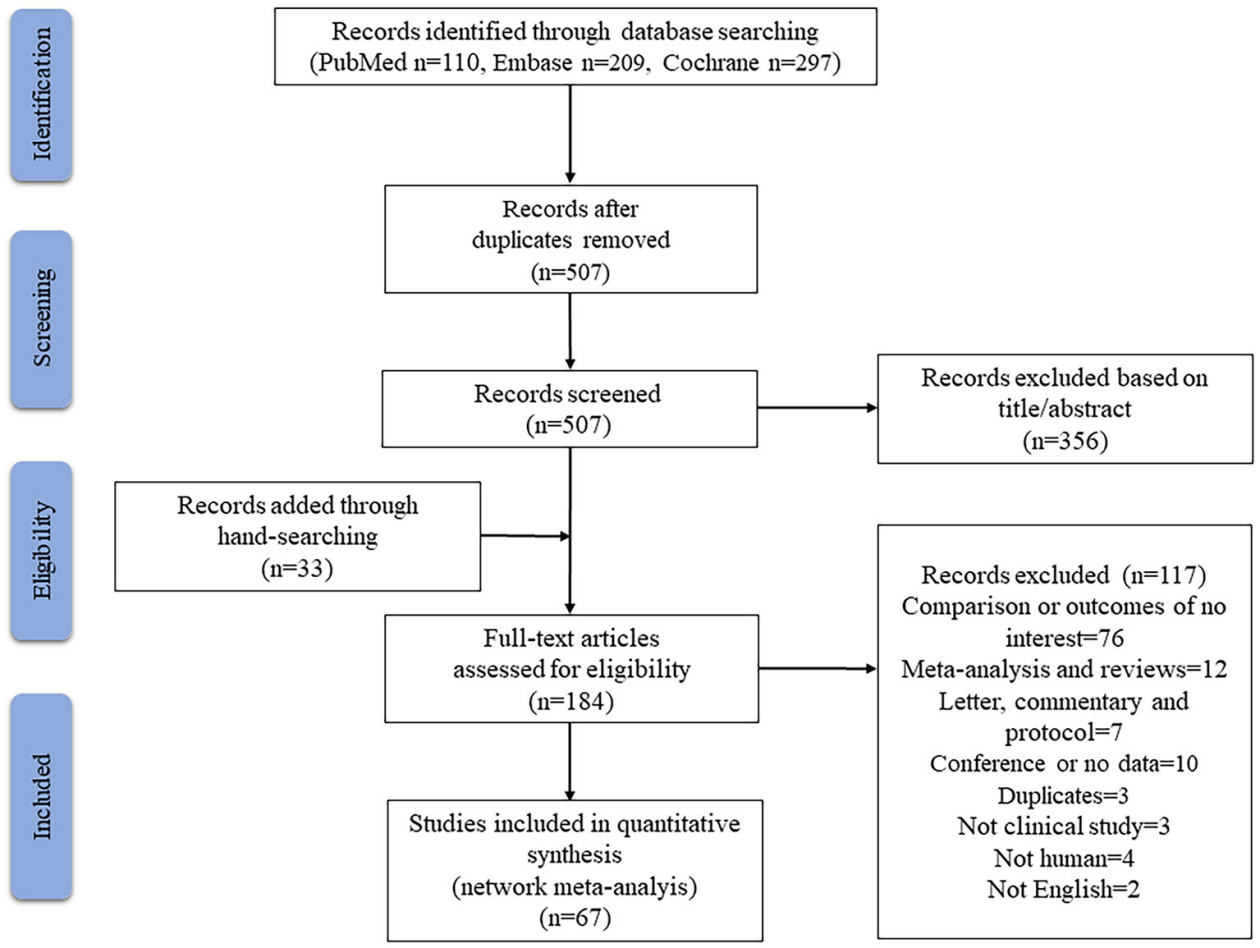
Flowchart for searching and identifying eligible studies.

The electronic literature search retrieved a total of 616 articles from PubMed, Embase, and Cochrane (Figure 1), of which 109 were excluded as duplicates and 356 were excluded based on their titles or abstracts. Manual searching of reference lists in relevant articles identified another 33 studies. After reviewing the full text of 184 studies, 67 were retained, comprising 18 RCTs and 49 observational cohort studies involving altogether 19,825 patients. Each study involved one or more of the following types of cardioplegia: BC, HTK, DN and St. Thomas (Supplementary Tables 4, 5).

### Risk of bias within studies

Assessment of bias in the 18 RCTs was summarized in Supplementary Table 6. One trial was judged as having low risk of bias, two as having high risk of selective reporting, and the remaining 15 as having unclear risks. There was insufficient information to assess the existence of other bias.

Bias in observational cohort studies was assessed using the Newcastle–Ottawa scale (Supplementary Table 7). All studies scored 6 or higher.

### Outcomes for adult patients

Among the 67 studies, 55 were adult trials involving 18,191 patients with a mean age from 40 to 75 years, the majority of whom were male (65%). Sample size per study ranged from 40 to 2,108 patients (median, 154). Median cross time ranged from 33 to 161 min. Median CPB time ranged from 52 to 215 min. BC was the most frequent type of cardioplegia. Potential effect modifiers showed transitivity across the trials. Participants were similar in median cross time, median CPB time, proportion of males and mean age for all four types of cardioplegia (Supplementary Figure 1). More detailed information was shown in Supplementary Tables 4, 5.

#### Perioperative mortality

**Figure 2:**
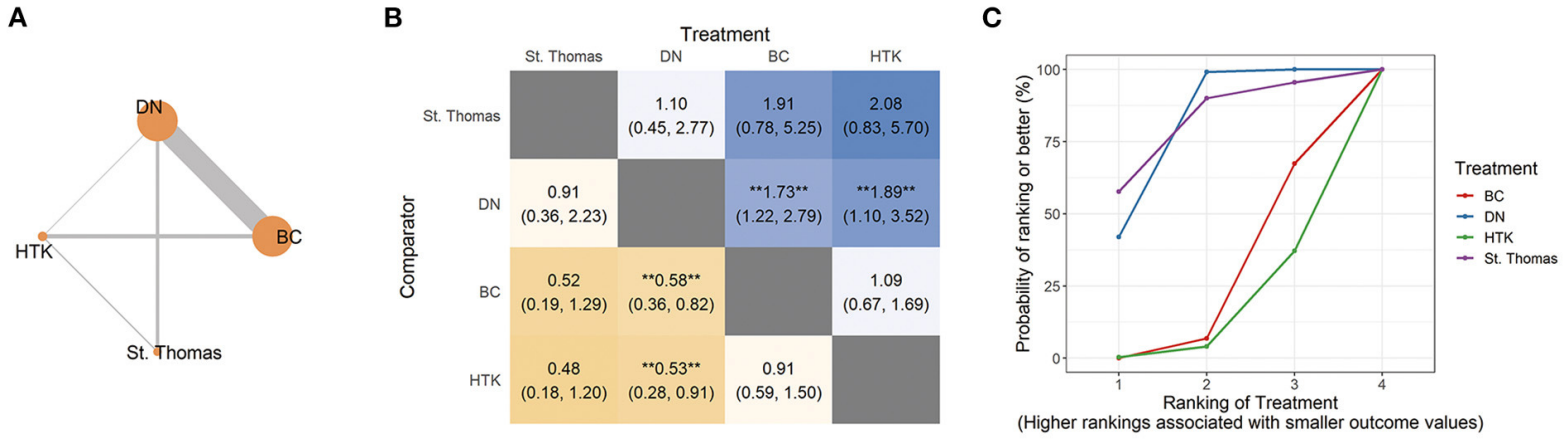
Network analysis of perioperative mortality across all adult trials. **(A)** The network plot shows the interventions included in the network analysis. Each node represents an intervention, and the thickness of connections between nodes reflects the number of studies in the comparison. **(B)** The league plot for perioperative mortality. The number in each cell refers to the comparison between the given column and row. Statistically significant results are marked with double asterisks. **(C)** Plot of the surface under the cumulative ranking curve (SUCRA). BC, blood cardioplegia; DN, del Nido cardioplegia; HTK, histidine-tryptophan-ketoglutarate cardioplegia.

Trials involving adults most often performed pairwise comparisons of BC with DN (30 trials, 10,008 patients) or HTK (6 trials, 5,011 patients; Figure 2A; Table 1). Risk of perioperative mortality was significantly higher for BC (RR 1.73, 95% CI 1.22, 2.79) and HTK (RR 1.89, 95% CI 1.10, 3.52) than for DN. In contrast, risk of mortality was similar for HTK as for BC (RR 1.09, 95% CI 0.67, 1.69; Figure 2B).

**Table 1:** Meta-analyses of trials directly comparing two types of cardioplegias.

**Patient group**	**Outcomes**	**Comparison**	**Studies**	**Sample size**	**Event 1**	**Event 2**	**RR/MD (95% CI)**	***P*-value**	***I^2^***
Adult	Perioperative mortality	DN vs. BC	30	10,008	90/4,732	116/5,276	0.86 (0.65, 1.15)	0.94	0
		DN vs. HTK	1	182	1/94	0/88	2.81 (0.12, 68.07)	-	-
		DN vs. St.Thomas	5	622	3/311	6/311	0.57 (0.17, 1.93)	0.97	0
		HTK vs. BC	6	5,011	78/1,672	111/3,339	1.21 (0.90, 1.64)	0.36	0.09
		HTK vs. St. Thomas	2	292	7/169	4/123	1.11 (0.34, 3.66)	-	-
	Atrial fibrillation	DN vs. BC	25	5,998	796/3,049	713/2,949	**1.09 (1.00, 1.19)**	0.15	0.23
		DN vs. HTK	1	182	8/94	4/88	1.87 (0.58, 6.00)	-	-
		DN vs. St.Thomas	3	1,286	81/490	124/796	1.08 (0.83, 1.39)	0.82	0
		HTK vs. BC	4	743	90/362	124/381	**0.73 (0.59, 0.91)**	0.97	0
		HTK vs. St. Thomas	1	104	11/54	14/50	0.73 (0.37, 1.45)	-	-
	Renal failure	DN vs. BC	17	4,694	110/2,325	129/2,369	0.89 (0.71, 1.12)	0.96	0
		DN vs. St.Thomas	1	200	2/100	3/100	0.67 (0.11, 3.90)	-	-
		HTK vs. BC	5	4,504	70/1,428	225/3,076	0.94 (0.71, 1.23)	0.2	0.34
	Stroke	DN vs. BC	24	6,332	59/3,116	52/3,216	1.18 (0.82, 1.70)	0.96	0
		DN vs. St.Thomas	1	132	0/66	1/66	0.33 (0.0.1, 8.04)	-	-
		HTK vs. BC	3	4,134	32/1,243	58/2,891	1.21 (0.75, 1.94)	0.55	0
		HTK vs. St. Thomas	1	104	0/54	0/50	-	-	-
	IABP	DN vs. BC	11	3,881	91/1,996	94/1,885	1.03 (0.77, 1.37)	0.43	0
		DN vs. HTK	1	182	8/94	5/88	1.50 (0.51, 4.41)	-	-
		DN vs. St.Thomas	2	300	4/150	6/150	0.69 (0.21, 2.25)	0.62	0
		HTK vs. BC	5	4,649	50/1,491	73/3,158	0.86 (0.60, 1.24)	0.9	0
		HTK vs. St. Thomas	1	188	5/115	2/73	1.59 (0.32, 7.97)	-	-
	Re-exploration	DN vs. BC	13	3,581	31/1,429	62/2,152	0.93 (0.60, 1.42)	0.66	0
		DN vs. St.Thomas	1	200	3/100	3/100	1.00 (0.21, 4.84)	-	-
		HTK vs. BC	3	2,596	91/1,298	91/1,298	1.00 (0.76, 1.31)	0.8	0
		HTK vs. St. Thomas	2	292	22/169	14/123	1.07 (0.57, 2.01)	0.27	0.18
	ICU stay	DN vs. BC	23	4,376	2,079	2,297	2.82 (−0.42, 6.06)	***p*** **<** **0.01**	0.72
		DN vs. HTK	2	222	115	107	−0.58 (−2.36, 1.19)	***p*** **<** **0.01**	0.92
		DN vs. St.Thomas	5	1,585	649	936	−2.40 (−5.69, 0.89)	**0.07**	0.53
		HTK vs. BC	7	1,409	697	712	−0.58 (−2.36, 1.19)	**0.07**	0.48
		HTK vs. St. Thomas	1	104	54	50	−14.40 (−45.24, 16.44)	-	-
	Hospital stay	DN vs. BC	22	4,686	2,386	2,300	0.16 (−0.06, 0.38)	***p*** **<** **0.01**	0.47
		DN vs. HTK	1	182	94	88	**0.60 (0.31, 0.89)**	**0.04**	-
		DN vs. St.Thomas	4	631	325	306	−0.04 (−0.86, 0.78)	***p*** **<** **0.01**	0.84
		HTK vs. BC	5	1,249	617	632	−0.36 (−0.77, 0.05)	**0.04**	0.61
Children	Perioperative mortality	DN vs. BC	2	281	2/137	6/144	0.34 (0.07, 1.70)	0.43	0
		DN vs. HTK	1	100	1/50	1/50	1.00 (0.06, 15.55)	-	-
		DN vs. St.Thomas	2	620	14/310	20/310	0.70 (0.36, 1.36)	0.35	0
		HTK vs. BC	3	313	7/145	8/168	1.06 (0.42, 2.69)	0.28	0.22
		HTK vs. St.Thomas	1	101	2/75	4/26	**0.17 (0.03, 0.89)**	-	-
	ICU stay	DN vs. BC	1	56	30	26	9.60 (−20.33, 39.53)	-	-
		DN vs. HTK	1	100	50	50	−4.75 (−11.10, 1.60)	-	-
		DN vs. St.Thomas	5	839	422	417	−2.71 (−39.67, 34.25)	***p*** **<** **0.01**	0.84
		HTK vs. BC	2	263	120	143	25.88 (−18.38, 70.13)	**0.09**	0.66
		HTK vs. St.Thomas	1	101	75	26	**-242.40 (−276.47,−208.33)**	-	-
	Hospital stay	DN vs. BC	2	281	137	144	−0.52 (−4.30, 3.26)	0.82	0
		DN vs. HTK	1	100	50	50	**-0.76 (−1.15,−0.37)**	-	-
		DN vs. St.Thomas	3	680	340	340	−0.53 (−2.92, 1.85)	**0.02**	0.74
		HTK vs. BC	2	263	120	143	−0.27 (−2.70, 2.15)	0.44	0
		HTK vs. St.Thomas	1	101	75	26	**-11.50 (−13.07,−9.93)**	-	-

Statistically significant results are shown in bold. BC, blood cardioplegia; CI, confidence interval; DN, del Nido cardioplegia; HTK, histidine-tryptophan-ketoglutarate cardioplegia; IABP, intra-aortic balloon pump; ICU, intensive care unit; MD, mean difference; RR, risk ratio.

SUCRA ranking based on direct and indirect comparisons assigned a higher ranking to St. Thomas. However, the NMA resulted in non-significant RRs with wide CIs: St. Thomas vs. DN, RR 0.91, 95% CI 0.36, 2.23; St. Thomas vs. BC, RR 0.52, 95% CI 0.19, 1.29; and St. Thomas vs. HTK, RR 0.48, 95% CI 0.18, 1.20 (Figures 2B,C).

Pairwise meta-analysis of types of cardioplegia revealed no significant differences (Table 1). The pooled data in these pairwise comparisons showed low heterogeneity, with *I*^2^ ranging from 0 to 9%.

#### Atrial fibrillation

**Figure 3:**
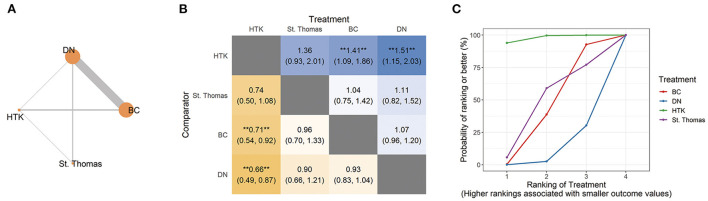
Network analysis of atrial fibrillation across all adult trials. **(A)** The network plot shows the interventions included in the network analysis. Each node represents an intervention, and the thickness of connections between nodes reflects the number of studies in the comparison. **(B)** The league plot for atrial fibrillation. The number in each cell refers to the comparison between the given column and row. Statistically significant results are marked with double asterisks. **(C)** Plot of the surface under the cumulative ranking curve (SUCRA). BC, blood cardioplegia; DN, del Nido cardioplegia; HTK, histidine-tryptophan-ketoglutarate cardioplegia.

Twenty-five trials involving 5,998 patients compared DN with BC for this outcome (Figure 3A; Table 1). In this NMA, the risk of atrial fibrillation was significantly higher for BC (RR 1.41, 95% CI 1.09, 1.86) and DN (RR 1.51, 95% CI 1.15, 2.03) than for HTK. Risk was similar among St. Thomas, BC and DN (Figure 3).

Pairwise meta-analysis indicated significantly higher risk of atrial fibrillation for DN than for BC (RR 1.09, 95% CI 1.00, 1.19; *I*^2^ = 23%; 25 studies, 5,998 patients). Consistent with the NMA, HTK was associated with lower risk than BC (RR 0.73, 95% CI 0.59, 0.91; *I*^2^ = 0%; 4 studies, 743 patients; Table 1).

#### Other outcomes

For most other outcomes, trials compared DN with BC: renal failure, 17 trials involving 4,694 patients; stroke, 24 trials involving 6,332 patients; IABP, 11 trials involving 3,881 patients; re-exploration, 13 trials involving 3,581 patients; ICU stay, 23 trials involving 4,376 patients; and hospital stay, 22 trials involving 4,686 patients. In the NMA, SUCRA rankings differed across the types of cardioplegia for different outcomes. Nevertheless, the four types did not differ significantly in risk of renal failure, stroke, IABP, re-exploration, or length of stay in the ICU or hospital (Table 1; Supplementary Figures 2–7).

Pairwise meta-analysis showed that DN was associated with longer length of hospital stay than HTK (MD 0.60, 95% CI 0.31, 0.89; 1 trial, 182 patients). No significant differences were observed in pairwise comparisons of renal failure, stroke, IABP, re-exploration or length of ICU stay (Table 1).

#### Subgroup analysis

For NMA of only RCTs, no significant differences were observed in perioperative mortality, atrial fibrillation, renal failure, length of ICU or hospital stay. Other outcomes could not be analyzed for lack of data (Supplementary Figures 8–13).

For NMA of only cohort studies, risk of perioperative mortality was significantly higher for BC (RR 1.71, 95% CI 1.19, 2.80) and HTK (RR 1.97, 95% CI 1.12, 3.91) than for DN. DN showed higher risk of atrial fibrillation than HTK (RR 1.48, 95% CI 1.01, 2.23). These results were consistent with NMA of all studies. No significant differences were observed in the other secondary outcomes (Supplementary Figures 14–22).

#### Network consistency

Node-splitting analysis did not detect significant inconsistency between direct and indirect evidence for any outcomes (*P* > 0.05; Supplementary Table 8). Neither comparison-adjusted funnel plots nor the Egger test indicated significant publication bias (*P* > 0.05; Supplementary Figure 23).

### Outcomes for pediatric patients

Twelve studies involved 1,634 pediatric patients, whose mean age ranged from 18.6 days to 8.7 years. Sample size ranged from 50 to 500 patients (median, 101), 53% of whom were male. Median cross time ranged from 48 to 165 min, and median CPB time from 66 to 233 min. Potential effect modifiers showed high transitivity across the trials (Supplementary Figure 24; Supplementary Tables 4, 5).

#### Perioperative mortality

**Figure 4:**
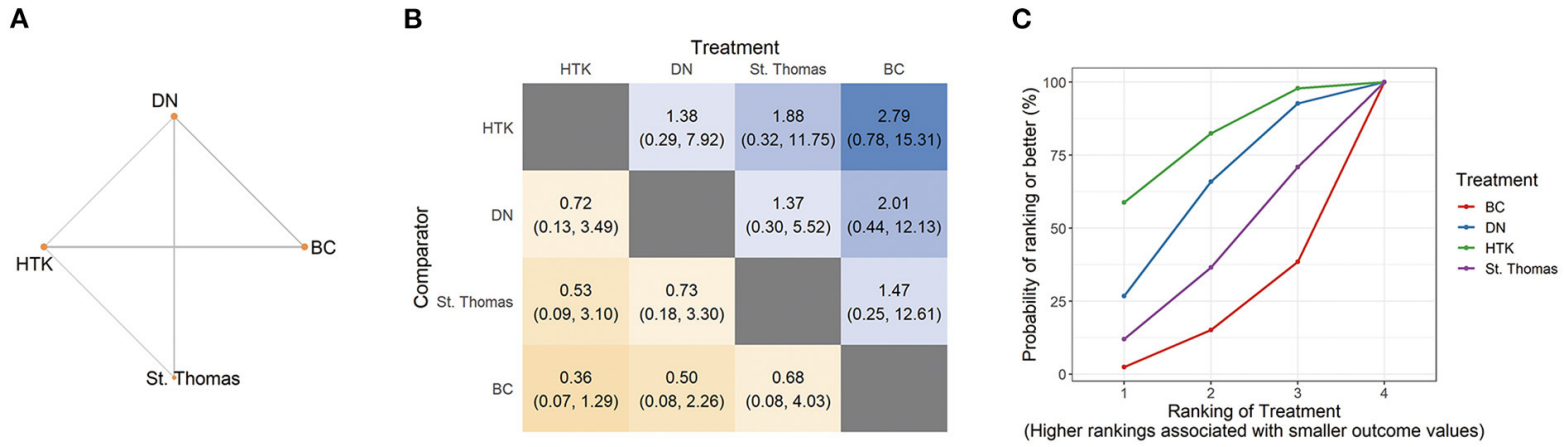
Network analysis of perioperative mortality across all pediatric trials. **(A)** The network plot shows the interventions included in the network analysis. Each node represents an intervention and the thickness of connections between nodes represent the number of studies involved in a comparison. **(B)** The league plot for perioperative mortality. The number in each cell refers to the comparison between the given column and row. **(C)** Plot of the surface under the cumulative ranking curve (SUCRA). BC, blood cardioplegia; DN, del Nido cardioplegia; HTK, histidine-tryptophan-ketoglutarate cardioplegia.

Synthesis of direct and indirect comparisons in the NMA showed that the four types of cardioplegia did not differ significantly in risk of mortality. The high proportion of indirect comparisons led to non-significant RRs with wide CIs: HTK vs. DN, RR 0.72, 95% CI 0.13, 3.49; HTK vs. St. Thomas, RR 0.53, 95% CI 0.09, 3.10; HTK vs. BC, RR 0.36, 95% CI 0.07, 1.29 (Figure 4). Nevertheless, HTK had a higher SUCRA value.

Pairwise meta-analysis of one trial indicated significantly lower risk of mortality with HTK than with St. Thomas (RR 0.17, 95% CI 0.03, 0.89, 101 patients; Table 1).

#### ICU and hospital stay

NMA indicated no significant differences in either ICU stay or hospital stay among the four types of cardioplegia. Nevertheless, SUCRA ranked HTK first and St. Thomas last for shorter ICU stay and hospital stay. Other outcomes could not be analyzed for lack of data (Supplementary Figures 25, 26).

Pairwise meta-analysis of one trial with 101 patients showed that HTK was associated with significantly shorter ICU stay (MD−242.40, 95% CI−276.47,−208.33) and hospital stay (MD−11.50, 95% CI−13.07,−9.93) than St. Thomas. Another study showed that DN was associated with significantly shorter hospital stay than HTK (MD−0.76, 95% CI−1.15,−0.37; Table 1).

#### Subgroup analysis

For NMA of only RCTs, risk of perioperative mortality was significantly higher for BC (RR 7.54, 95% CI 1.13, 96.04) than for HTK. These results should be interpreted with caution because only BC, DN and HTK were directly compared, and the CIs are extremely broad. Length of ICU and hospital stay did not differ among the four types of cardioplegia (Supplementary Figures 27–30).

For NMA of only cohort studies, no significant differences were observed in perioperative mortality, length of ICU or hospital stay (Supplementary Figures 31–34).

#### Network consistency

Node-splitting analysis did not detect significant disagreement between direct and indirect evidence for outcomes in pediatric patients, except for comparisons of ICU stay for DN and HTK vs. St. Thomas (*P* < 0.05; Supplementary Table 8). Funnel plots were not generated for pediatric trials since fewer than 10 studies were pooled for each outcome.

## Discussion

Ideally cardioplegia should achieve rapid diastolic arrest and show satisfactory myocardial protection, reversibility and low toxicity (2). Since the concept of chemical cardiac arrest was first proposed in the 1950s (24), different types of cardioplegia have been developed and compared, but it is unclear which types are optimal for different types of cardiac surgery patients. Systematic reviews and meta-analyses have come to conflicting conclusions (25–30). To our knowledge, the present NMA provides the first indication that both HTK and BC may be associated with higher risk of perioperative mortality than DN in adult patients. In children, however, HTK might be of greater benefit than the other three types of cardioplegia in terms of perioperative mortality, ICU stay and hospital stay, though the differences among the four types did not achieve statistical significance in our analyses. This study may help guide the selection of cardioplegia for adult and pediatric patients undergoing cardiac surgery.

Previous meta-analyses of adult patients reported comparable risk of mortality for different types of cardioplegia, but the analyses diverged on whether different types were associated with similar risk of adverse cardiac events. For example, one study (27) concluded that compared to crystalloid cardioplegia, BC was associated with lower risk of low output syndrome (LOS) and early increase in creatine kinase–myocardial band (CK-MB), but similar risk of myocardial infarction (MI). Another study (30) reported lower incidence of perioperative MI with BC than crystalloid cardioplegia, but similar incidences of other cardiac events. A meta-analysis (29) found no difference in LOS or MI between BC and crystalloid cardioplegia. It is difficult to compare those previous studies with one another or with the present work because of differences in sample size as well as potential differences in risk factors among the patients and in the type of crystalloid cardioplegia, which was usually not specified.

Our work goes beyond an NMA of seven types of cardioplegia in adult cardiac surgery (31). That analysis, based on studies retrieved up to 29 November 2020, defined the primary outcome as serum concentrations of myocardial injury markers. Comparisons mostly involved cold, warm or warm terminal BC and/or crystalloid cardioplegia, but not adequate in St. Thomas or HTK. That analysis reported no significant differences among the seven types of cardioplegia, but this may reflect the small samples of the included studies. The present NMA drew on data published through 31 December 2021 to compare four types of cardioplegia in terms of the primary outcome of perioperative mortality.

In fact, the present NMA draws on a large sample to provide perhaps the most detailed comparison of outcomes among four well-defined types of cardioplegia. The NMA showed that, among adult patients, DN may be associated with lower risk of perioperative mortality than HTK or BC. On the other hand, HTK may be associated with lower risk of atrial fibrillation than DN and BC. Pairwise meta-analysis also suggested a lower risk of atrial fibrillation with HTK than with BC. However, NMA and pairwise meta-analysis did not agree on how DN compared with HTK or BC in terms of atrial fibrillation. DN is a diluted variation of BC, which provides oxygen for myocardial aerobic metabolism, as well as low levels of calcium ion and small numbers of leukocytes. This may mitigate calcium overload and leukocyte-induced inflammation. HTK, for its part, has been shown to protect the myocardium for up to 3 h after a single dose (32). On one hand, the prolonged intermittent myocardial perfusion in HTK may aggravate myocardial ischemia injuries; on the other hand, the perfusion solution in HTK may promote ATP-generating glycolysis, inhibit inflammation by generating NO, and neutralize acidosis. As a result, atrial muscle consumes little oxygen during HTK, which may reduce the risk of atrial fibrillation.

Similar with previous meta-analysis of outcomes (28), our NMA also showed that there was no difference between blood or crystalloid types of cardioplegia with regard to mortality, length of ICU stay or hospital stay in pediatric cardiac surgery. However, in NMA involving only RCTs, HTK was associated with significantly lower risk of mortality than BC for children. This discrepancy may reflect the anatomical, functional and metabolic differences between immature and adult myocardium, which make the pediatric heart more resistant to ischemia than adult heart (33). Up to 90% of ATP in myocardium is generated through the oxidation of fatty acids in adults, but through the oxidation of glucose in children (34). Tryptophan and ketoglutaric acid in HTK enhance myocardial glycolysis during ischemia and maintain high levels of intracellular ATP *via* two energy-generating pathways (35), which may explain its superior performance in pediatric cardiac surgery. At the same time, the lack of calcium in HTK may alleviate intracellular calcium overload during ischemia/reperfusion, to which pediatric myocardium is more sensitive than adult myocardium (36). On the other hand, HTK can induce hyponatremia, which may lead to postoperative seizures in pediatric patients (35). Few pediatric studies in our NMA reported data on this complication, so further study of HTK and pediatric cardiac protection is needed.

### Limitations

First, we did not limit our NMA to RCTs, since we considered that increasing the overall sample by adding high-quality observational cohort studies could improve the level of evidence. To assess the effects of the different types of study, NMA was re-analyzed based on data only from RCTs or only from cohort studies. Nevertheless, patients receiving St. Thomas showed significantly younger mean age than those receiving HTK in cohort studies of adults. In addition, the proportions of male patients receiving St. Thomas differed significantly from those receiving BC or HTK among RCTs of adults. Thus, our results from subgroup analyses should be interpreted with caution.

Second, the size of pooled samples for each type of cardioplegia varied widely, from 9,433 patients in 50 trials for BC and 6,465 patients in 51 trials for DN, to 2,325 patients in 19 trials for HTK or 1,602 patients in 15 trials for St. Thomas. This variation may make our comparisons less reliable, particularly those involving St. Thomas. Large RCTs involving St. Thomas are needed for future study.

Third, although the NMA showed statistical consistency, we cannot entirely exclude the presence of imbalances in effect modifiers and therefore residual confounding bias. Such modifiers may include the complexity of surgery, anesthetic method, type and extent of cardiac disease, and overall myocardial protection strategy (37). Variations in these modifiers may help explain differences between our NMA and pairwise meta-analysis. The EuroSCORE can assess the risk of heart surgery (38), but we were unable to compare this score across types of cardioplegia because most studies did not report it at the patient level. More evidence from RCTs and meta-analysis are needed to define the benefit–risk profiles for different types of cardioplegia in cardiac surgery.

Fourth, our study included relatively few pediatric trials, preventing us from conducting subgroup analyses based on age or cyanotic differences. Such analyses are important for determining which types of cardioplegia provide better myocardial protection to pediatric patients.

Finally, we searched only three research databases and considered only studies published in English. We also started with studies from 2005 because before that year, most studies focused on comparing only BC and crystalloid cardioplegia (27). This may increase risk of selection bias. Nevertheless, we did not detect evidence of significant publication bias or bias in the transitivity of potential effect modifiers.

## Conclusions

This NMA of four types of cardioplegia widely used during cardiac surgery suggests that in adult patients, DN may be associated with lower perioperative mortality than HTK and BC, while HTK may be associated with lower atrial fibrillation risk than DN and BC. Large, multicenter RCTs are needed to thoroughly define benefit–risk profiles for different types of cardioplegia in cardiac surgery.

## Data availability statement

The raw data supporting the conclusions of this article will be made available by the authors, without undue reservation.

## Author contributions

JG raised the idea of this study and developed strategies for study selection. JL and JT searched for the studies. JT designed and wrote the manuscript. Statistical analysis was completed by SB. YW, JX, and XY participated in data collection and quality assessment of the included studies. JG and LD provided the consultations and revised the manuscript. All authors contributed to the article and approved the submitted version.

## Funding

This work was supported by the 1·3·5 Project for Disciplines of Excellence-Clinical Research Incubation Project of West China Hospital, Sichuan University, China (Grant No. 2017-120).

## Conflict of interest

The authors declare that the research was conducted in the absence of any commercial or financial relationships that could be construed as a potential conflict of interest.

## Publisher's note

All claims expressed in this article are solely those of the authors and do not necessarily represent those of their affiliated organizations, or those of the publisher, the editors and the reviewers. Any product that may be evaluated in this article, or claim that may be made by its manufacturer, is not guaranteed or endorsed by the publisher.

## Abbreviations

BC: blood cardioplegia
CI: confidence interval
CPB: cardiopulmonary bypass
DN: del Nido cardioplegia
HTK: histidine-tryptophan-ketoglutarate cardioplegia
IABP: intra-aortic balloon pump
ICU: intensive care unit
MD: mean difference
RCT: randomized controlled trial
RR: risk ratio.
