# Supplementary material for: Comparative effects of different types of cardioplegia in cardiac surgery: A network meta-analysis
Source: Front Cardiovasc Med. 2022 Sep 13;9:996744. doi: 10.3389/fcvm.2022.996744 (PMC9513158; doi:10.3389/fcvm.2022.996744)
Supplement: Supplementary file 1 [file Data_Sheet_1.DOCX]

**Supplementary Figure 1.** Transitivity analysis of each cardioplegia type across all adult patients. A, Frequency of interventions published in clinical trials. B, Median cross time (min). C, Median CPB time (min). D, Proportion of males. E, Mean age (year). Means were compared in a pairwise fashion using Student’s t test and across general groups using ANOVA. P < 0.05 was considered significant. ANOVA, [analysis of variance](https://www.investopedia.com/terms/a/anova.asp); BC, blood cardioplegia; CPB, cardiopulmonary bypass; DN, del Nido cardioplegia; HTK, histidine-tryptophan-ketoglutarate cardioplegia.

**Supplementary Figure 2.** Network analysis of renal failure across all adult trials. A, The network plot shows the interventions included in the network analysis. Each node represents an intervention, and the thickness of connections between nodes reflects the number of studies in the comparison. B, The league plot for renal failure. The number in each cell refers to the comparison between the given column and row. C, Plot of the surface under the cumulative ranking curve (SUCRA). BC, blood cardioplegia; DN, del Nido cardioplegia; HTK, histidine-tryptophan-ketoglutarate cardioplegia.

**Supplementary Figure 3.** Network analysis of stroke across all adult trials. A, The network plot shows the interventions included in the network analysis. Each node represents an intervention, and the thickness of connections between nodes reflects the number of studies in the comparison. B, The league plot for stroke. The number in each cell refers to the comparison between the given column and row. C, Plot of the surface under the cumulative ranking curve (SUCRA). BC, blood cardioplegia; DN, del Nido cardioplegia; HTK, histidine-tryptophan-ketoglutarate cardioplegia.

**Supplementary Figure 4.** Network analysis of the use of intra-aortic balloon pump across all adult trials. A, The network plot shows the interventions included in the network analysis. Each node represents an intervention, and the thickness of connections between nodes reflects the number of studies in the comparison. B, The league plot for intra-aortic balloon pump. The number in each cell refers to the comparison between the given column and row. C, Plot of the surface under the cumulative ranking curve (SUCRA). BC, blood cardioplegia; DN, del Nido cardioplegia; HTK, histidine-tryptophan-ketoglutarate cardioplegia.

**Supplementary Figure 5.** Network analysis of re-exploration across all adult trials. A, The network plot shows the interventions included in the network analysis. Each node represents an intervention, and the thickness of connections between nodes reflects the number of studies in the comparison. B, The league plot for re-exploration. The number in each cell refers to the comparison between the given column and row. C, Plot of the surface under the cumulative ranking curve (SUCRA). BC, blood cardioplegia; DN, del Nido cardioplegia; HTK, histidine-tryptophan-ketoglutarate cardioplegia.

**Supplementary Figure 6.** Network analysis of ICU stay across all adult trials. A, The network plot shows the interventions included in the network analysis. Each node represents an intervention, and the thickness of connections between nodes reflects the number of studies in the comparison. B, The league plot for ICU saty. The number in each cell refers to the comparison between the given column and row. C, Plot of the surface under the cumulative ranking curve (SUCRA). BC, blood cardioplegia; DN, del Nido cardioplegia; HTK, histidine-tryptophan-ketoglutarate cardioplegia; ICU, intensive care unit.

**Supplementary Figure 7.** Network analysis of hospital stay across all adult trials. A, The network plot shows the interventions included in the network analysis. Each node represents an intervention, and the thickness of connections between nodes reflects the number of studies in the comparison. B, The league plot for hospital stay. The number in each cell refers to the comparison between the given column and row. C, Plot of the surface under the cumulative ranking curve (SUCRA). BC, blood cardioplegia; DN, del Nido cardioplegia; HTK, histidine-tryptophan-ketoglutarate cardioplegia.

**Supplementary Figure 8.** Transitivity analysis of each cardioplegia type across adult patients only in RCTs. A, Frequency of interventions published in clinical trials. B, Median cross time (min). C, Median CPB time (min). D, Proportion of males. E, Mean age (year). Means were compared in a pairwise fashion using Student’s t test and across general groups using ANOVA. P < 0.05 was considered significant. ANOVA, [analysis of variance](https://www.investopedia.com/terms/a/anova.asp); BC, blood cardioplegia; CPB, cardiopulmonary bypass; DN, del Nido cardioplegia; HTK, histidine-tryptophan-ketoglutarate cardioplegia.

**Supplementary Figure 9.** Network analysis of perioperative mortality across adult patients only in RCTs. A, The network plot shows the interventions included in the network analysis. Each node represents an intervention, and the thickness of connections between nodes reflects the number of studies in the comparison. B, The league plot for perioperative mortality. The number in each cell refers to the comparison between the given column and row. C, Plot of the surface under the cumulative ranking curve (SUCRA). BC, blood cardioplegia; DN, del Nido cardioplegia; HTK, histidine-tryptophan-ketoglutarate cardioplegia; RCT, randomized controlled trial.

**Supplementary Figure 10.** Network analysis of atrial fibrillation across adult patients only in RCTs. A, The network plot shows the interventions included in the network analysis. Each node represents an intervention, and the thickness of connections between nodes reflects the number of studies in the comparison. B, The league plot for atrial fibrillation. The number in each cell refers to the comparison between the given column and row. C, Plot of the surface under the cumulative ranking curve (SUCRA). BC, blood cardioplegia; DN, del Nido cardioplegia; HTK, histidine-tryptophan-ketoglutarate cardioplegia; RCT, randomized controlled trial.

**Supplementary Figure 11.** Network analysis of renal failure across adult patients only in RCTs. A, The network plot shows the interventions included in the network analysis. Each node represents an intervention, and the thickness of connections between nodes reflects the number of studies in the comparison. B, The league plot for renal failure. The number in each cell refers to the comparison between the given column and row. C, Plot of the surface under the cumulative ranking curve (SUCRA). BC, blood cardioplegia; DN, del Nido cardioplegia; HTK, histidine-tryptophan-ketoglutarate cardioplegia; RCT, randomized controlled trial.

**Supplementary Figure 12.** Network analysis of ICU stay across adult patients only in RCTs. A, The network plot shows the interventions included in the network analysis. Each node represents an intervention, and the thickness of connections between nodes reflects the number of studies in the comparison. B, The league plot for ICU stay. The number in each cell refers to the comparison between the given column and row. C, Plot of the surface under the cumulative ranking curve (SUCRA). BC, blood cardioplegia; DN, del Nido cardioplegia; HTK, histidine-tryptophan-ketoglutarate cardioplegia; ICU, intensive care unit; RCT, randomized controlled trial.

**Supplementary Figure 13.** Network analysis of hospital stay across adult patients only in RCTs. A, The network plot shows the interventions included in the network analysis. Each node represents an intervention, and the thickness of connections between nodes reflects the number of studies in the comparison. B, The league plot for hospital stay. The number in each cell refers to the comparison between the given column and row. C, Plot of the surface under the cumulative ranking curve (SUCRA). BC, blood cardioplegia; DN, del Nido cardioplegia; HTK, histidine-tryptophan-ketoglutarate cardioplegia; RCT, randomized controlled trial.

**Supplementary Figure 14.** Transitivity analysis of each cardioplegia type across adult patients only in cohort studies. A, Frequency of interventions published in clinical trials. B, Median cross time (min). C, Median CPB time (min). D, Proportion of males. E, Mean age (year). Means were compared in a pairwise fashion using Student’s t test and across general groups using ANOVA. P < 0.05 was considered significant. ANOVA, [analysis of variance](https://www.investopedia.com/terms/a/anova.asp); BC, blood cardioplegia; CPB, cardiopulmonary bypass; DN, del Nido cardioplegia; HTK, histidine-tryptophan-ketoglutarate cardioplegia.

**Supplementary Figure 15.** Network analysis of perioperative mortality across adult patients only in cohort studies. A, The network plot shows the interventions included in the network analysis. Each node represents an intervention, and the thickness of connections between nodes reflects the number of studies in the comparison. B, The league plot for perioperative mortality. The number in each cell refers to the comparison between the given column and row. Statistically significant results are marked with double asterisks. C, Plot of the surface under the cumulative ranking curve (SUCRA). BC, blood cardioplegia; DN, del Nido cardioplegia; HTK, histidine-tryptophan-ketoglutarate cardioplegia.

**Supplementary Figure 16.** Network analysis of atrial fibrillation across adult patients only in cohort studies. A, The network plot shows the interventions included in the network analysis. Each node represents an intervention, and the thickness of connections between nodes reflects the number of studies in the comparison. B, The league plot for atrial fibrillation. The number in each cell refers to the comparison between the given column and row. Statistically significant results are marked with double asterisks. C, Plot of the surface under the cumulative ranking curve (SUCRA). BC, blood cardioplegia; DN, del Nido cardioplegia; HTK, histidine-tryptophan-ketoglutarate cardioplegia.

**Supplementary Figure 17.** Network analysis of renal failure across adult patients only in cohort studies. A, The network plot shows the interventions included in the network analysis. Each node represents an intervention, and the thickness of connections between nodes reflects the number of studies in the comparison. B, The league plot for renal failure. The number in each cell refers to the comparison between the given column and row. C, Plot of the surface under the cumulative ranking curve (SUCRA). BC, blood cardioplegia; DN, del Nido cardioplegia; HTK, histidine-tryptophan-ketoglutarate cardioplegia.

**Supplementary Figure 18.** Network analysis of stroke across adult patients only in cohort studies. A, The network plot shows the interventions included in the network analysis. Each node represents an intervention, and the thickness of connections between nodes reflects the number of studies in the comparison. B, The league plot for stroke. The number in each cell refers to the comparison between the given column and row. C, Plot of the surface under the cumulative ranking curve (SUCRA). BC, blood cardioplegia; DN, del Nido cardioplegia; HTK, histidine-tryptophan-ketoglutarate cardioplegia.

**Supplementary Figure 19.** Network analysis of the use of intra-aortic balloon pump across adult patients only in cohort studies. A, The network plot shows the interventions included in the network analysis. Each node represents an intervention, and the thickness of connections between nodes reflects the number of studies in the comparison. B, The league plot for intra-aortic balloon pump. The number in each cell refers to the comparison between the given column and row. C, Plot of the surface under the cumulative ranking curve (SUCRA). BC, blood cardioplegia; DN, del Nido cardioplegia; HTK, histidine-tryptophan-ketoglutarate cardioplegia.

**Supplementary Figure 20.** Network analysis of re-exploration across adult patients only in cohort studies. A, The network plot shows the interventions included in the network analysis. Each node represents an intervention, and the thickness of connections between nodes reflects the number of studies in the comparison. B, The league plot for re-exploration. The number in each cell refers to the comparison between the given column and row. C, Plot of the surface under the cumulative ranking curve (SUCRA). BC, blood cardioplegia; DN, del Nido cardioplegia; HTK, histidine-tryptophan-ketoglutarate cardioplegia.

**Supplementary Figure 21.** Network analysis of ICU stay across adult patients only in cohort studies. A, The network plot shows the interventions included in the network analysis. Each node represents an intervention, and the thickness of connections between nodes reflects the number of studies in the comparison. B, The league plot for ICU saty. The number in each cell refers to the comparison between the given column and row. C, Plot of the surface under the cumulative ranking curve (SUCRA). BC, blood cardioplegia; DN, del Nido cardioplegia; HTK, histidine-tryptophan-ketoglutarate cardioplegia; ICU, intensive care unit.

**Supplementary Figure 22.** Network analysis of hospital stay across adult patients only in cohort studies. A, The network plot shows the interventions included in the network analysis. Each node represents an intervention, and the thickness of connections between nodes reflects the number of studies in the comparison. B, The league plot for hospital stay. The number in each cell refers to the comparison between the given column and row. C, Plot of the surface under the cumulative ranking curve (SUCRA). BC, blood cardioplegia; DN, del Nido cardioplegia; HTK, histidine-tryptophan-ketoglutarate cardioplegia.

**Supplementary Figure 23.** Comparison-adjusted funnel plots for the network describing each outcome based on all trials involving adults. A, Perioperative mortality. B, Atrial fibrillation. C, Renal failure. D, Stroke. E, Intra-aortic balloon pump. F, Re-exploration. G, ICU stay. H, Hospital stay. Each observation is the difference between a study estimate and the mean effect based on direct meta-analysis. Each color represents a comparison between interventions. P > 0.05 indicates no significant publication bias. BC, blood cardioplegia; DN, del Nido cardioplegia; HTK, histidine-tryptophan-ketoglutarate cardioplegia; ICU, intensive care unit.

**Supplementary Figure 24.** Transitivity analysis of each cardioplegia type across all pediatric patients. A, Frequency of interventions published in clinical trials. B, Median cross time (min). C, Median CPB time (min). D, Proportion of males. E, Mean age (year). A Means were compared in a pairwise fashion using Student’s t test and across general groups using ANOVA. P < 0.05 was considered significant. ANOVA, [analysis of variance](https://www.investopedia.com/terms/a/anova.asp); BC, blood cardioplegia; CPB, cardiopulmonary bypass; DN, del Nido cardioplegia; HTK, histidine-tryptophan-ketoglutarate cardioplegia.

**Supplementary Figure 25.** Network analysis of ICU stay across all pediatric trials. A, The network plot shows the interventions included in the network analysis. Each node represents an intervention, and the thickness of connections between nodes reflects the number of studies in the comparison. B, The league plot for ICU stay. The number in each cell refers to the comparison between the given column and row. C, Plot of the surface under the cumulative ranking curve (SUCRA). BC, blood cardioplegia; DN, del Nido cardioplegia; HTK, histidine-tryptophan-ketoglutarate cardioplegia; ICU, intensive care unit.

**Supplementary Figure 26.** Network analysis of hospital stay across all pediatric trials. A, The network plot shows the interventions included in the network analysis. Each node represents an intervention, and the thickness of connections between nodes reflects the number of studies in the comparison. B, The league plot for hospital stay. The number in each cell refers to the comparison between the given column and row. C, Plot of the surface under the cumulative ranking curve (SUCRA). BC, blood cardioplegia; DN, del Nido cardioplegia; HTK, histidine-tryptophan-ketoglutarate cardioplegia.

**Supplementary Figure 27.** Transitivity analysis of each cardioplegia type across pediatric patients only in RCTs. A, Frequency of interventions published in clinical trials. B, Median cross time (min). C, Median CPB time (min). D, Proportion of males. E, Mean age (year). Means were compared in a pairwise fashion using Student’s t test and across general groups using ANOVA. P < 0.05 was considered significant. ANOVA, [analysis of variance](https://www.investopedia.com/terms/a/anova.asp); BC, blood cardioplegia; CPB, cardiopulmonary bypass; DN, del Nido cardioplegia; HTK, histidine-tryptophan-ketoglutarate cardioplegia.

**Supplementary Figure 28.** Network analysis of perioperative mortality across pediatric patients only in RCTs. A, The network plot shows the interventions included in the network analysis. Each node represents an intervention, and the thickness of connections between nodes reflects the number of studies in the comparison. B, The league plot for perioperative mortality. The number in each cell refers to the comparison between the given column and row. Statistically significant results are marked with double asterisks. C, Plot of the surface under the cumulative ranking curve (SUCRA). BC, blood cardioplegia; DN, del Nido cardioplegia; HTK, histidine-tryptophan-ketoglutarate cardioplegia; RCT, randomized controlled trial.

**Supplementary Figure 29.** Network analysis of ICU stay across pediatric patients only in RCTs. A, The network plot shows the interventions included in the network analysis. Each node represents an intervention, and the thickness of connections between nodes reflects the number of studies in the comparison. B, The league plot for ICU stay. The number in each cell refers to the comparison between the given column and row. C, Plot of the surface under the cumulative ranking curve (SUCRA). BC, blood cardioplegia; DN, del Nido cardioplegia; HTK, histidine-tryptophan-ketoglutarate cardioplegia; ICU, intensive care unit; RCT, randomized controlled trial.

**Supplementary Figure 30.** Network analysis of hospital stay across pediatric patients only in RCTs. A, The network plot shows the interventions included in the network analysis. Each node represents an intervention, and the thickness of connections between nodes reflects the number of studies in the comparison. B, The league plot for hospital stay. The number in each cell refers to the comparison between the given column and row. C, Plot of the surface under the cumulative ranking curve (SUCRA). BC, blood cardioplegia; DN, del Nido cardioplegia; HTK, histidine-tryptophan-ketoglutarate cardioplegia; RCT, randomized controlled trial.

**Supplementary Figure 31.** Transitivity analysis of each cardioplegia type across pediatric patients only in cohort studies. A, Frequency of interventions published in clinical trials. B, Median cross time (min). C, Median CPB time (min). D, Proportion of males. E, Mean age (year). Means were compared in a pairwise fashion using Student’s t test and across general groups using ANOVA. P < 0.05 was considered significant. ANOVA, [analysis of variance](https://www.investopedia.com/terms/a/anova.asp); BC, blood cardioplegia; CPB, cardiopulmonary bypass; DN, del Nido cardioplegia; HTK, histidine-tryptophan-ketoglutarate cardioplegia.

**Supplementary Figure 32.** Network analysis of perioperative mortality across pediatric patients only in cohort studies. A, The network plot shows the interventions included in the network analysis. Each node represents an intervention, and the thickness of connections between nodes reflects the number of studies in the comparison. B, The league plot for perioperative mortality. The number in each cell refers to the comparison between the given column and row. C, Plot of the surface under the cumulative ranking curve (SUCRA). BC, blood cardioplegia; DN, del Nido cardioplegia; HTK, histidine-tryptophan-ketoglutarate cardioplegia; RCT, randomized controlled trial.

**Supplementary Figure 33.** Network analysis of ICU stay across pediatric patients only in cohort studies. A, The network plot shows the interventions included in the network analysis. Each node represents an intervention, and the thickness of connections between nodes reflects the number of studies in the comparison. B, The league plot for ICU stay. The number in each cell refers to the comparison between the given column and row. C, Plot of the surface under the cumulative ranking curve (SUCRA). BC, blood cardioplegia; DN, del Nido cardioplegia; HTK, histidine-tryptophan-ketoglutarate cardioplegia; ICU, intensive care unit; RCT, randomized controlled trial.

**Supplementary Figure 34.** Network analysis of hospital stay across pediatric patients only in cohort studies. A, The network plot shows the interventions included in the network analysis. Each node represents an intervention, and the thickness of connections between nodes reflects the number of studies in the comparison. B, The league plot for hospital stay. The number in each cell refers to the comparison between the given column and row. C, Plot of the surface under the cumulative ranking curve (SUCRA). BC, blood cardioplegia; DN, del Nido cardioplegia; HTK, histidine-tryptophan-ketoglutarate cardioplegia; RCT, randomized controlled trial.
