# Supplementary material for: Comparative effects of different types of cardioplegia in cardiac surgery: A network meta-analysis
Source: Front Cardiovasc Med. 2022 Sep 13;9:996744. doi: 10.3389/fcvm.2022.996744 (PMC9513158; doi:10.3389/fcvm.2022.996744)
Supplement: Supplementary file 2 [file Data_Sheet_2.docx]

List of Supplementary Tables and Figures

Table S1. PubMed searches. 3-6

Table S2. Embase searches. 7-8

Table S3. Cochrane searches. 9-10

Table S4. Characteristics of the 67 studies in the network meta-analysis. 11-16

Table S5. Administration of four types of cardioplegia and types of cardiac surgery in the included studies. 17-22

Table S6. Summary of risk of bias in the included randomized controlled trials. 23

Table S7. Quality of the observational cohort studies, based on the Newcastle–Ottawa scale. 24-26

Table S8. Node-splitting analysis of the network meta-analysis in order to assess consistency between direct and indirect evidence. 27-29

Figure S1. Transitivity analysis of each cardioplegia type across all adult patients. 30

Figure S2. NMA of renal failure across all adult trials. 31

Figure S3. NMA of stroke across all adult trials. 32

Figure S4. NMA of the use of intra-aortic balloon pump across all adult trials. 33

Figure S5. NMA of re-exploration across all adult trials. 34

Figure S6. NMA of ICU stay across all adult trials. 35

Figure S7. NMA of hospital stay across all adult trials. 36

Figure S8. Transitivity analysis of each cardioplegia type across adult patients only in RCTs. 37

Figure S9. NMA of perioperative mortality across adult patients only in RCTs. 38

Figure S10. NMA of atrial fibrillation across adult patients only in RCTs. 39

Figure S11. NMA of renal failure across adult patients only in RCTs. 40

Figure S12. NMA of ICU stay across adult patients only in RCTs. 41

Figure S13. NMA of hospital stay across adult patients only in RCTs. 42

Figure S14. Transitivity analysis of each cardioplegia type across adult patients only in cohort studies. 43

Figure S15. NMA of perioperative mortality across adult patients only in cohort studies. 44

Figure S16. NMA of atrial fibrillation across adult patients only in cohort studies. 45

Figure S17. NMA of renal failure across adult patients only in cohort studies. 46

Figure S18. NMA of stroke across adult patients only in cohort studies. 47

Figure S19. NMA of the use of intra-aortic balloon pump across adult patients only in cohort studies. 48

Figure S20. NMA of re-exploration across adult patients only in cohort studies. 49

Figure S21. NMA of ICU stay across adult patients only in cohort studies. 50

Figure S22. NMA of hospital stay across adult patients only in cohort studies. 51

Figure S23. Comparison-adjusted funnel plots. 52

Figure S24. Transitivity analysis of each cardioplegia type across all pediatric patients. 53

Figure S25. NMA of ICU stay across all pediatric trials. 54

Figure S26. NMA of hospital stay across all pediatric trials. 55

Figure S27. Transitivity analysis of each cardioplegia type across pediatric patients only in RCTs. 56

Figure S28. NMA of perioperative mortality across pediatric patients only in RCTs. 57

Figure S29. NMA of ICU stay across pediatric patients only in RCTs. 58

Figure S30. NMA of hospital stay across pediatric patients only in RCTs. 59

Figure S31. Transitivity analysis of each cardioplegia type across pediatric patients only in cohort studies. 60

Figure S32. NMA of perioperative mortality across pediatric patients only in cohort studies. 61

Figure S33. NMA of ICU stay across pediatric patients only in cohort studies. 62

Figure S34. NMA of hospital stay across pediatric patients only in cohort studies. 63

Supplementary References 64-70

PRISMA NMA Checklist ……………………………………………………….71-75

Supplementary Table 1. PubMed searches.

| No. | Query | No. of results |
| --- | --- | --- |
| 17 | (("Thoracic Surgery"[MeSH Terms] OR ("surgery thoracic"[Title/Abstract] OR "surgery cardiac"[Title/Abstract] OR "surgery heart"[Title/Abstract] OR "heart surgery"[Title/Abstract] OR "cardiac surgery"[Title/Abstract])) AND ("del nido"[Title/Abstract] OR ("Bretschneider cardioplegic solution"[Supplementary Concept] OR ("custodiol solution"[Title/Abstract] OR "htk solution"[Title/Abstract] OR "htk solution of bretschneider"[Title/Abstract] OR "bretschneider solution"[Title/Abstract] OR "histidine tryptophan ketoglutarate solution"[Title/Abstract])) OR ("st thomas hospital cardioplegic solution"[Supplementary Concept] OR ("sth solution"[Title/Abstract] OR (("cardioplegic"[All Fields] OR "cardioplegically"[All Fields]) AND "soln"[All Fields] AND "STH"[Title/Abstract]) OR "Plegisol"[Title/Abstract])) OR "blood cardioplegia"[Title/Abstract]) AND ("randomized controlled trial"[Publication Type] OR "randomized"[Title/Abstract] OR "placebo"[Title/Abstract] OR ("prognosis"[MeSH Terms:noexp] OR "diagnosed"[Title/Abstract] OR "cohort*"[Title/Abstract] OR "cohort effect"[MeSH Terms] OR "cohort studies"[MeSH Terms:noexp] OR "predictor*"[Title/Abstract] OR "death"[Title/Abstract] OR "models, statistical"[MeSH Terms])) AND 2005/01/01:2021/12/31[Date - Publication]) AND (2005/1/1:2021/12/31[pdat]) | 110 |
| 16 | ((("Thoracic Surgery"[Mesh]) OR (((((Surgery, Thoracic[Title/Abstract]) OR (Surgery, Cardiac[Title/Abstract])) OR (Surgery, Heart[Title/Abstract])) OR (Heart Surgery[Title/Abstract])) OR (Cardiac Surgery[Title/Abstract]))) AND ((((Del Nido[Title/Abstract]) OR (("Bretschneider cardioplegic solution" [Supplementary Concept]) OR (((((Custodiol solution[Title/Abstract]) OR (HTK solution[Title/Abstract])) OR (HTK solution of Bretschneider[Title/Abstract])) OR (Bretschneider solution[Title/Abstract])) OR (histidine-tryptophan-ketoglutarate solution[Title/Abstract])))) OR (("St. Thomas' Hospital cardioplegic solution" [Supplementary Concept]) OR (((STH solution[Title/Abstract]) OR (cardioplegic soln STH[Title/Abstract])) OR (Plegisol[Title/Abstract])))) OR (blood cardioplegia[Title/Abstract]))) AND ((randomized controlled trial[Publication Type] OR randomized[Title/Abstract] OR placebo[Title/Abstract]) OR (prognosis[MeSH:noexp] OR diagnosed[Title/Abstract] OR cohort*[Title/Abstract] OR cohort effect[MeSH] OR cohort studies[MeSH:noexp] OR predictor*[Title/Abstract] OR death[Title/Abstract] OR "models, statistical"[MeSH])) | 176 |
| 15 | (randomized controlled trial[Publication Type] OR randomized[Title/Abstract] OR placebo[Title/Abstract]) OR (prognosis[MeSH:noexp] OR diagnosed[Title/Abstract] OR cohort*[Title/Abstract] OR cohort effect[MeSH] OR cohort studies[MeSH:noexp] OR predictor*[Title/Abstract] OR death[Title/Abstract] OR "models, statistical"[MeSH]) | 3,910,414 |
| 14 | prognosis[MeSH:noexp] OR diagnosed[Title/Abstract] OR cohort*[Title/Abstract] OR cohort effect[MeSH] OR cohort studies[MeSH:noexp] OR predictor*[Title/Abstract] OR death[Title/Abstract] OR "models, statistical"[MeSH] | 3,118,293 |
| 13 | randomized controlled trial[Publication Type] OR randomized[Title/Abstract] OR placebo[Title/Abstract] | 935,907 |
| 12 | (((Del Nido[Title/Abstract]) OR (("Bretschneider cardioplegic solution" [Supplementary Concept]) OR (((((Custodiol solution[Title/Abstract]) OR (HTK solution[Title/Abstract])) OR (HTK solution of Bretschneider[Title/Abstract])) OR (Bretschneider solution[Title/Abstract])) OR (histidine-tryptophan-ketoglutarate solution[Title/Abstract])))) OR (("St. Thomas' Hospital cardioplegic solution" [Supplementary Concept]) OR (((STH solution[Title/Abstract]) OR (cardioplegic soln STH[Title/Abstract])) OR (Plegisol[Title/Abstract])))) OR (blood cardioplegia[Title/Abstract]) | 2,479 |
| 11 | blood cardioplegia[Title/Abstract] | 1,320 |
| 10 | ("St. Thomas' Hospital cardioplegic solution" [Supplementary Concept]) OR (((STH solution[Title/Abstract]) OR (cardioplegic soln STH[Title/Abstract])) OR (Plegisol[Title/Abstract])) | 373 |
| 9 | ((STH solution[Title/Abstract]) OR (cardioplegic soln STH[Title/Abstract])) OR (Plegisol[Title/Abstract]) | 55 |
| 8 | "St. Thomas' Hospital cardioplegic solution" [Supplementary Concept] | 347 |
| 7 | ("Bretschneider cardioplegic solution" [Supplementary Concept]) OR (((((Custodiol solution[Title/Abstract]) OR (HTK solution[Title/Abstract])) OR (HTK solution of Bretschneider[Title/Abstract])) OR (Bretschneider solution[Title/Abstract])) OR (histidine-tryptophan-ketoglutarate solution[Title/Abstract])) | 781 |
| 6 | ((((Custodiol solution[Title/Abstract]) OR (HTK solution[Title/Abstract])) OR (HTK solution of Bretschneider[Title/Abstract])) OR (Bretschneider solution[Title/Abstract])) OR (histidine-tryptophan-ketoglutarate solution[Title/Abstract]) | 553 |
| 5 | "Bretschneider cardioplegic solution" [Supplementary Concept] | 545 |
| 4 | Del Nido[Title/Abstract] | 172 |
| 3 | ("Thoracic Surgery"[Mesh]) OR (((((Surgery, Thoracic[Title/Abstract]) OR (Surgery, Cardiac[Title/Abstract])) OR (Surgery, Heart[Title/Abstract])) OR (Heart Surgery[Title/Abstract])) OR (Cardiac Surgery[Title/Abstract])) | 70,774 |
| 2 | ((((Surgery, Thoracic[Title/Abstract]) OR (Surgery, Cardiac[Title/Abstract])) OR (Surgery, Heart[Title/Abstract])) OR (Heart Surgery[Title/Abstract])) OR (Cardiac Surgery[Title/Abstract]) | 63,335 |
| 1 | "Thoracic Surgery"[Mesh] | 13,245 |

Supplementary Table 2. Embase searches.

| No. | Query | No. of results |
| --- | --- | --- |
| 17 | #3 AND #12 AND #15 AND [humans]/lim AND [english]/lim AND [clinical study]/lim AND [embase]/lim AND [2005-2021]/py | 209 |
| 16 | #3 AND #12 AND #15 | 429 |
| 15 | #13 OR #14 | 3,871,089 |
| 14 | 'prognosis':ab,ti OR 'cohort':ab,ti OR 'cohort effect':ab,ti OR 'cohort studies':ab,ti OR 'predictor':ab,ti OR 'death':ab,ti OR 'models, statistical':ab,ti | 2,957,535 |
| 13 | 'randomized controlled trial':ab,ti OR 'randomized':ab,ti OR 'placebo':ab,ti | 1,036,885 |
| 12 | #4 OR #7 OR #10 OR #11 | 2,718 |
| 11 | 'blood cardioplegia'/exp | 15 |
| 10 | #8 OR #9 | 486 |
| 9 | 'St. Thomas hospital cardioplegic solution':ab,ti OR 'sth solution':ab,ti OR 'cardioplegic soln sth':ab,ti OR 'plegisol':ab,ti | 238 |
| 8 | 'st thomas hospital solution'/exp | 308 |
| 7 | #5 OR #6 | 1,358 |
| 6 | 'bretschneider cardioplegic solution':ab,ti OR 'custodiol solution':ab,ti OR 'htk solution of bretschneider':ab,ti OR 'bretschneider solution':ab,ti OR 'histidine-tryptophan-ketoglutarate solution':ab,ti | 340 |
| 5 | 'htk solution'/exp | 1,214 |
| 4 | 'del nido' OR (del AND nido) | 957 |
| 3 | #1 OR #2 | 1,037,992 |
| 2 | 'surgery, thoracic':ab,ti OR 'surgery, cardiac':ab,ti OR 'surgery, heart':ab,ti OR 'thoracic surgery':ab,ti OR 'cardiac surgery':ab,ti | 92,930 |
| 1 | 'heart surgery'/exp OR 'heart surgery' OR (('heart'/exp OR heart) AND ('surgery'/exp OR surgery)) | 1,015,841 |

Supplementary Table 3. Cochrane searches.

| No. | Query | No. of results |
| --- | --- | --- |
| 1 | MeSH descriptor: [Thoracic Surgery] explode all trees | 174 |
| 2 | (Surgery, Thoracic):ti,ab,kw OR (Surgery, Cardiac):ti,ab,kw OR (Surgery, Heart):ti,ab,kw OR (Heart Surgery):ti,ab,kw OR (Cardiac Surgery):ti,ab,kw | 41678 |
| 3 | #1 OR #2 | 41678 |
| 4 | Del Nido | 76 |
| 5 | (Bretschneider cardioplegic solution):ti,ab,kw OR (Custodiol solution):ti,ab,kw OR (HTK solution):ti,ab,kw OR (HTK solution of Bretschneider):ti,ab,kw OR (Bretschneider solution):ti,ab,kw OR (histidine-tryptophan-ketoglutarate solution):ti,ab,kw | 125 |
| 6 | (St. Thomas' Hospital cardioplegic solution):ti,ab,kw OR (STH solution):ti,ab,kw OR (cardioplegic soln STH):ti,ab,kw OR (Plegisol):ti,ab,kw | 47 |
| 7 | blood cardioplegia | 781 |
| 8 | #4 OR #5 OR #6 OR #7 | 920 |
| 9 | (randomized controlled trial):ti,ab,kw OR (randomized):ti,ab,kw OR (placebo):ti,ab,kw | 1108718 |
| 10 | (prognosis):ti,ab,kw OR (cohort):ti,ab,kw OR (cohort effect):ti,ab,kw OR (cohort studies):ti,ab,kw OR (predictor):ti,ab,kw OR (death):ti,ab,kw OR (models, statistical):ti,ab,kw | 170179 |
| 11 | #9 OR #10 | 1152620 |
| 12 | #3 AND #8 AND #11 with Cochrane Library publication date Between Jan 2005 and Dec 2021, in Trials | 297 |

Supplementary Table 4. Characteristics of the 67 studies in the network meta-analysis.

| Author | Year | Country | Intervention | Total sample | Male proportion | | Mean age (year) | Median cross time (min) | Median CPB time (min) | Study type |  |  |  |  |
| --- | --- | --- | --- | --- | --- | --- | --- | --- | --- | --- | --- | --- | --- | --- |
| Ad et al^E1^ | 2018 | USA | DN | 48 | 83% | 65.3 | | NR | NR | RCT |  |  |  |  |
|  |  |  | BC | 41 | 76% | 65.1 | | NR | NR |  |  |  |  |  |
| Ali et al^E2^ | 2021 | Egypt | HTK | 160 | 74% | 44.19 | | 101.53 | 141.06 | RCT |  |  |  |  |
|  |  |  | BC | 160 | 75% | 43.11 | | 147.92 | 197.74 |  |  |  |  |  |
| Arafat et al^E3^ | 2021 | Egypt | DN | 73 | 78% | 61 | | 97 | 131.9 | Cohort study |  |  |  |  |
|  |  |  | BC | 81 | 80% | 65 | | 107.5 | 141.2 |  |  |  |  |  |
| Bigdelian et al^E4^ | 2020 | Iran | DN | 30 | 60% | 1.65 | | 71.33 | 98.33 | RCT |  |  |  |  |
|  |  |  | St. Thomas | 30 | 53% | 1.86 | | 67.47 | 92.87 |  |  |  |  |  |
| Braathen et al^E5^ | 2011 | Norway | HTK | 38 | 66% | 59 | | 75 | 107 | RCT |  |  |  |  |
|  |  |  | BC | 38 | 89% | 59 | | 73 | 103 |  |  |  |  |  |
| Busro et al^E6^ | 2018 | Indonesia | HTK | 54 | 54% | 25.5 (months) | | 64.25 | 110.03 | RCT |  |  |  |  |
|  |  |  | BC | 55 | 58% | 23 (months) | | 67.46 | 179.22 |  |  |  |  |  |
| Caneo et al^E7^ | 2021 | Brazil | DN | 250 | 49% | 2.8 | | 70 | 102 | Cohort study |  |  |  |  |
|  |  |  | St. Thomas | 250 | 48% | 3.1 | | 64 | 93 |  |  |  |  |  |
| Cayir et al^E8^ | 2020 | Turkey | DN | 100 | NR | NR | | 53.2 | 86.4 | Cohort study |  |  |  |  |
|  |  |  | St. Thomas | 100 | NR | NR | | 63.8 | 98.2 |  |  |  |  |  |
| Chen et al^E9^ | 2021 | China | DN | 62 | 50% | 55.3 | | 93.2 | 165 | Cohort study |  |  |  |  |
|  |  |  | BC | 60 | 60% | 54.9 | | 91.8 | 168 |  |  |  |  |  |
| Cvetković et al^E10^ | 2021 | Serbia | HTK | 54 | 74% | 64.5 | | 49.1 | 82.2 | RCT |  |  |  |  |
|  |  |  | St. Thomas | 50 | 88% | 65.3 | | 41 | 74.5 |  |  |  |  |  |
| Elassal et al^E11^ | 2020 | Egypt | DN | 60 | 62% | 8.88 (months) | | 48 | 66 | Cohort study |  |  |  |  |
|  |  |  | St. Thomas | 60 | 48% | 10.92 (months) | | 48.5 | 66 |  |  |  |  |  |
| Gaudino et al^E12^ | 2013 | Italy | HTK | 31 | 81% | 64 | | 71 | 92 | RCT |  |  |  |  |
|  |  |  | BC | 29 | 72% | 61 | | 73 | 95 |  |  |  |  |  |
| George et al^13^ | 2020 | India | DN | 143 | 70% | 48.2 | | 59 | 90 | Cohort study |  |  |  |  |
|  |  |  | BC | 143 | 60% | 46.7 | | 57 | 87 |  |  |  |  |  |
| Gorjipour et al^E14^ | 2017 | Iran | DN | 32 | 53% | 2.33 | | 96.47 | 137.47 | RCT |  |  |  |  |
|  |  |  | St. Thomas | 27 | 33% | 2.02 | | 82.04 | 127.64 |  |  |  |  |  |
| Guajardo et al^E15^ | 2017 | USA | DN | 159 | 65% | 62.49 | | 52.3 | 64.41 | Cohort study |  |  |  |  |
|  |  |  | BC | 249 | 75% | 64.1 | | 50.97 | 63.67 |  |  |  |  |  |
| Gunaydin et al^E16^ | 2020 | Turkey | HTK | 88 | 64% | 68 | | 79 | NR | Cohort study |  |  |  |  |
|  |  |  | DN | 94 | 65% | 71 | | 81 | NR |  |  |  |  |  |
|  |  |  | BC | 107 | 67% | 73 | | 82 | NR |  |  |  |  |  |
| Haan et al^E17^ | 2020 | Netherlands | HTK | 115 | 70% | 68.4 | | 125.6 | 179.3 | Cohort study |  |  |  |  |
|  |  |  | St. Thomas | 73 | 70% | 68.7 | | 93.1 | 129.6 |  |  |  |  |  |
| Hamad et al^E18^ | 2017 | Canada | DN | 25 | 80% | 73.8 | | 55.6 | 65.5 | Cohort study |  |  |  |  |
|  |  |  | BC | 25 | 80% | 74.7 | | 64.3 | 76.6 |  |  |  |  |  |
| Haranal et al^E19^ | 2020 | Malaysia | DN | 50 | 58% | 1.99 | | 60 | 97 | RCT |  |  |  |  |
|  |  |  | St. Thomas | 50 | 50% | 1.67 | | 57 | 92 |  |  |  |  |  |
| Hoyer et al^E20^ | 2017 | Germany | HTK | 825 | 57% | 68 | | 55 | 78 | Cohort study |  |  |  |  |
|  |  |  | BC | 825 | 59% | 68 | | 52 | 77 |  |  |  |  |  |
| Hummel et al^E21^ | 2016 | Germany | HTK | 181 | 67% | 72 | | 80 | 106 | Cohort study |  |  |  |  |
|  |  |  | BC | 181 | 67% | 71 | | 89 | 120 |  |  |  |  |  |
| Jakub et al^E22^ | 2019 | Poland | DN | 1236 | 70% | 66.6 | | 40 | 65 | Cohort study |  |  |  |  |
|  |  |  | BC | 872 | 75% | 66.2 | | 33 | 55 |  |  |  |  |  |
| Khaled et al^E23^ | 2020 | Saudi Arabia | DN | 231 | 61% | 55 | | 118 | 150 | Cohort study |  |  |  |  |
|  |  |  | BC | 74 | 62% | 58 | | 143 | 178 |  |  |  |  |  |
| Kim et al^E24^ | 2018 | Austria | DN | 149 | 61% | 61.7 | | 76.1 | 98.6 | Cohort study |  |  |  |  |
|  |  |  | BC | 892 | 54% | 59.3 | | 94.8 | 148.4 |  |  |  |  |  |
| Kim et al^E25^ | 2016 | South Korea | DN | 39 | 54% | 56 | | 97 | 158 | Cohort study |  |  |  |  |
|  |  |  | BC | 39 | 59% | 54 | | 88 | 157 |  |  |  |  |  |
| Koda et al^E26^ | 2021 | USA | DN | 254 | 47% | 65 | | 107 | 155 | Cohort study |  |  |  |  |
|  |  |  | BC | 254 | 51% | 64.9 | | 116 | 147 |  |  |  |  |  |
| Koeckert et al^E27^ | 2018 | USA | DN | 59 | 78% | 69.1 | | 58.1 | 77.6 | Cohort study |  |  |  |  |
|  |  |  | BC | 122 | 56% | 69.7 | | 53.5 | 71 |  |  |  |  |  |
| Kuserli et al^E28^ | 2020 | Turkey | DN | 36 | 69% | 65.5 | | 96.75 | 129.39 | Cohort study |  |  |  |  |
|  |  |  | BC | 36 | 72% | 67 | | 116.42 | 151.94 |  |  |  |  |  |
| Lama et al^E29^ | 2021 | Nepal | DN | 45 | 80% | 59.98 | | 66.22 | 106.13 | RCT |  |  |  |  |
|  |  |  | St. Thomas | 45 | 84% | 57.51 | | 72.07 | 107.62 |  |  |  |  |  |
| Lenoir et al^E30^ | 2021 | Canada | DN | 55 | 80% | 48.6 | | 145 | 163 | Cohort study |  |  |  |  |
|  |  |  | BC | 55 | 73% | 48.7 | | 161 | 181 |  |  |  |  |  |
| Li et al^E31^ | 2016 | China | HTK | 75 | 51% | 3.4 | | 165.4 | 233.2 | Cohort study |  |  |  |  |
|  |  |  | St. Thomas | 26 | 62% | 1.9 | | 142.6 | 226 |  |  |  |  |  |
| Luo et al^E32^ | 2019 | China | DN | 66 | 52% | 50.5 | | 56.5 | 98 | Cohort study |  |  |  |  |
|  |  |  | St. Thomas | 66 | 53% | 52.5 | | 61.5 | 105.5 |  |  |  |  |  |
| Marzouk et al^E33^ | 2020 | Canada | DN | 131 | 73% | 65.8 | | 81.1 | 106.3 | Cohort study |  |  | 81.1 | 106.3 |
|  |  |  | BC | 251 | 71% | 65.8 | | 99.4 | 125.1 |  |  |  | 99.4 | 125.1 |
| Mehrabanian et al^E34^ | 2018 | Iran | DN | 21 | 33% | 57.14 | | 73.76 | 103.19 | RCT |  |  |  |  |
|  |  |  | HTK | 19 | 37% | 59.47 | | 83.96 | 97.36 |  |  |  |  |  |
| Mercan et al^E35^ | 2020 | Turkey | HTK | 25 | 76% | 60.1 | | 54.5 | 94.2 | RCT |  |  |  |  |
|  |  |  | BC | 25 | 84% | 62.7 | | 49.7 | 93.3 |  |  |  |  |  |
| Mick et al^E36^ | 2015 | USA | DN | 195 | 70% | 68 | | 67 | 98 | Cohort study |  |  |  |  |
|  |  |  | BC | 195 | 67% | 69 | | 71 | 100 |  |  |  |  |  |
| Mishra et al^E37^ | 2016 | India | DN | 50 | 74% | 52.48 | | 110.15 | 158.6 | Cohort study |  |  |  |  |
|  |  |  | St. Thomas | 50 | 78% | 50.96 | | 133.56 | 179.81 |  |  |  |  |  |
| Negi et al^E38^ | 2019 | India | DN | 30 | 50% | 8.7 | | 98.2 | 143.5 | RCT |  |  |  |  |
|  |  |  | BC | 26 | 69% | 7.6 | | 97.3 | 146.7 |  |  |  |  |  |
| O'Donnell et al^E39^ | 2019 | USA | DN | 54 | 83% | 63 | | 74.4 | 98.3 | Cohort study |  |  |  |  |
|  |  |  | BC | 27 | 74% | 61.3 | | 87.4 | 114.5 |  |  |  |  |  |
| Orak et al^40^ | 2020 | Turkey | DN | 43 | 53% | 54.95 | | 77.93 | 114.58 | Cohort study |  |  |  |  |
|  |  |  | BC | 40 | 55% | 59.83 | | 70.73 | 112.43 |  |  |  |  |  |
| Ota et al^E41^ | 2015 | USA | DN | 54 | 70% | 71.4 | | 52 | 60 | Cohort study |  |  |  |  |
|  |  |  | BC | 54 | 67% | 71.5 | | 71 | 84 |  |  |  |  |  |
| Palo et al^E42^ | 2017 | Italy | HTK | 46 | 52% | 59 | | 106 | 150 | Cohort study |  |  |  |  |
|  |  |  | BC | 44 | 48% | 54 | | 88 | 129 |  |  |  |  |  |
| Pizano et al^E43^ | 2018 | Colombia | HTK | 292 | 58% | 57.7 | | 98.5 | 147.5 | Cohort study |  |  |  |  |
|  |  |  | BC | 292 | 64% | 58.7 | | 116.1 | 132.8 |  |  |  |  |  |
| Pourmoghadam et al^E44^ | 2017 | USA | DN | 107 | 56% | 85 (days) | | 90 | 186 | Cohort study |  |  |  |  |
|  |  |  | BC | 118 | 60% | 98 (days) | | 100 | 175 |  |  |  |  |  |
| Pragliola et al^E45^ | 2020 | Arabia | DN | 102 | 73% | 54 | | 108 | NR | Cohort study |  |  |  |  |
|  |  |  | BC | 102 | 76% | 52 | | 106 | NR |  |  |  |  |  |
| Qulisy et al^E46^ | 2016 | Saudi Arabia | HTK | 66 | 56% | 291.50 (days) | | 65 | 98 | Cohort study |  |  |  |  |
|  |  |  | BC | 88 | 47% | 305.50 (days) | | 49 | 77 |  |  |  |  |  |
| Rai et al^E47^ | 2021 | India | DN | 40 | 75% | 66.6 | | 50 | 60 | Cohort study |  |  |  |  |
|  |  |  | BC | 40 | 65% | 68.7 | | 70 | 90 |  |  |  |  |  |
| Reidy et al^E48^ | 2021 | USA | DN | 70 | 100% | 67 | | 63.9 | 96.8 | Cohort study |  |  |  |  |
|  |  |  | BC | 85 | 99% | 66 | | 71.7 | 117 |  |  |  |  |  |
| Ross et al^E49^ | 2021 | Australia | DN | 171 | 62% | 62 | | 67 | 96 | Cohort study |  |  |  |  |
|  |  |  | BC | 326 | 71% | 64 | | 63 | 96 |  |  |  |  |  |
| Sanetra et al^E50^ | 2019 | Poland | DN | 75 | 64% | 62.53 | | 55.18 | 67.96 | RCT |  |  |  |  |
|  |  |  | BC | 75 | 51% | 63.83 | | 55.52 | 69.25 |  |  |  |  |  |
| Sanri et al^E51^ | 2021 | Turkey | DN | 132 | 80% | 61.67 | | 59.3 | 95.16 | Cohort study |  |  |  |  |
|  |  |  | BC | 123 | 74% | 60.78 | | 62.8 | 90.01 |  |  |  |  |  |
| Schutz et al^E52^ | 2020 | USA | DN | 420 | 74% | 66.2 | | 32.817 | 53.086 | Cohort study |  |  |  |  |
|  |  |  | BC | 443 | 79% | 66.6 | | 33.284 | 52.099 |  |  |  |  |  |
| Sharma et al^E53^ | 2021 | India | DN | 114 | 54% | 42.16 | | 72.6 | 92.1 | Cohort study |  |  |  |  |
|  |  |  | St. Thomas | 95 | 59% | 41.17 | | 98.2 | 129.5 |  |  |  |  |  |
| Shi et al^E54^ | 2020 | China | DN | 43 | 84% | 58.63 | | 84.72 | 123.88 | Cohort study |  |  |  |  |
|  |  |  | BC | 43 | 70% | 55.88 | | 82.63 | 123.09 |  |  |  |  |  |
| Shu et al^E55^ | 2021 | China | DN | 324 | 52% | 60.44 | | 80 | 116 | Cohort study |  |  |  |  |
|  |  |  | St. Thomas | 630 | 58% | 58.96 | | 61 | 89 |  |  |  |  |  |
| Talwar et al^E56^ | 2019 | India | DN | 50 | 72% | 4.45 | | 85.04 | 126.04 | RCT |  |  |  |  |
|  |  |  | HTK | 50 | 66% | 5.1 | | 87.06 | 128.92 |  |  |  |  |  |
| Timek et al^E57^ | 2016 | USA | DN | 100 | NR | 66.4 | | 68.7 | 86.6 | Cohort study |  |  |  |  |
|  |  |  | BC | 100 | NR | 65.1 | | 74.9 | 93.2 |  |  |  |  |  |
| Timek et al^E58^ | 2019 | USA | DN | 501 | 77% | 66 | | 69 | 81 | Cohort study |  |  |  |  |
|  |  |  | BC | 350 | 79% | 65 | | 86 | 99 |  |  |  |  |  |
| Ucak et al^E59^ | 2019 | Turkey | DN | 112 | 62% | 62.4 | | 43.7 | 67.9 | RCT |  |  |  |  |
|  |  |  | BC | 185 | 56% | 63.7 | | 54.3 | 77.2 |  |  |  |  |  |
| Ucak et al^E60^ | 2021 | Turkey | DN | 52 | 65% | 64.02 | | 41.5 | 53.7 | Cohort study |  |  |  |  |
|  |  |  | BC | 85 | 60% | 63.24 | | 48.7 | 60.8 |  |  |  |  |  |
| Vaidya et al^E61^ | 2020 | Nepal | DN | 50 | 70% | 41.75 | | 65.45 | 119.35 | Cohort study |  |  |  |  |
|  |  |  | St. Thomas | 50 | 50% | 40.35 | | 56.75 | 106.5 |  |  |  |  |  |
| Valente et al^E62^ | 2019 | Brazil | HTK | 25 | 36% | 28.5 (days) | | 57 | 80.3 | RCT |  |  |  |  |
|  |  |  | BC | 25 | 40% | 18.6 (days) | | 54.2 | 79.8 |  |  |  |  |  |
| Viana et al^E63^ | 2013 | Australia | HTK | 126 | 71% | 64 | | 153 | 215 | Cohort study |  |  |  |  |
|  |  |  | BC | 1774 | 71% | 65 | | 103 | 139 |  |  |  |  |  |
| Vistarini et al^E64^ | 2017 | Canada | DN | 25 | NR | 71 | | 56 | 67 | Cohort study |  |  |  |  |
|  |  |  | BC | 21 | NR | 72 | | 48 | 59 |  |  |  |  |  |
| Vivacqua et al^E65^ | 2019 | USA | HTK | 55 | 53% | 63 | | 74 | 99 | RCT |  |  |  |  |
|  |  |  | BC | 55 | 64% | 70 | | 77 | 102 |  |  |  |  |  |
| Yerebakan et al^E66^ | 2014 | USA | DN | 40 | 63% | 68.7 | | NR | NR | Cohort study |  |  |  |  |
|  |  |  | BC | 40 | 53% | 66.6 | | NR | NR |  |  |  |  |  |
| Ziazadeh et al^E67^ | 2017 | USA | DN | 63 | 65% | 66 | | 80 | 108 | Cohort study |  |  |  |  |
|  |  |  | BC | 63 | 60% | 67 | | 102 | 135 |  |  |  |  |  |

BC, blood cardioplegia; CPB, cardiopulmonary bypass; DN, del Nido cardioplegia; HTK, Histidine-tryptophan-ketoglutarate cardioplegia; NR, not reported; RCT, randomized controlled trial.

Supplementary Table 5. Administration of four types of cardioplegia and types of cardiac surgery in the included studies.

| Study | Intervention | Temperature (℃) | | Interval (min) | | Route | Patient type | Types of surgery |
| --- | --- | --- | --- | --- | --- | --- | --- | --- |
| Ad et al. 2018 | DN | 6-10 | 90 | | Antegrade/Retrograde | | Adult | First-time CABG, valve, or CABG/valve surgery |
|  | BC | 8-11 | 20 | | Antegrade/Retrograde | |  |  |
| Ali et al. 2021 | HTK | 4 | - | | Antegrade | | Adult | Multiple cardiac surgery |
|  | BC | 29 | 30-45 | | Antegrade | |  |  |
| Arafat et al. 2021 | DN | 4 | 90 | | Antegrade | | Adult | CABG, valve, or CABG/valve surgery |
|  | BC | NR | NR | | NR | |  |  |
| Bigdelian et al. 2020 | DN | NR | NR | | NR | | Children | Complete heart repair surgery of TOF |
|  | St. Thomas | NR | NR | | NR | |  |  |
| Braathen et al. 2011 | HTK | 4-8 | - | | Antegrade | | Adult | Elective mitral valve surgery |
|  | BC | 4-8 | 20 | | Antegrade | |  |  |
| Busro et al. 2018 | HTK | 4-10 | - | | NR | | Children | Biventricular repair |
|  | BC | 34-36 | NR | | NR | |  |  |
| Caneo et al. 2021 | DN | NR | 90 | | NR | | Children | Congenital cardiac surgery (RACHS-1) |
|  | St. Thomas | NR | 20 | | NR | |  |  |
| Cayir et al. 2020 | DN | 4-6 | - | | Antegrade | | Adult | Isolated CABG |
|  | St. Thomas | 4-7 | 20 | | Antegrade | |  |  |
| Chen et al. 2021 | DN | NR | 90 | | Antegrade | | Adult | Acute aortic dissection surgery |
|  | BC | 4 | 30 | | Antegrade/Retrograde | |  |  |
| Cvetković et al. 2021 | HTK | 4-8 | 120 | | Antegrade | | Adult | Primary isolated CABG |
|  | St. Thomas | 4-8 | 20 | | Antegrade | |  |  |
| Elassal et al. 2020 | DN | 10-12 | - | | Antegrade | | Children | CABG or valve surgery |
|  | St. Thomas | 10-12 | 20 | | Antegrade | |  |  |
| Gaudino et al. 2013 | HTK | NR | - | | Antegrade | | Adult | Mitral valve surgery |
|  | BC | NR | 15 | | Antegrade | |  |  |
| George et al. 2020 | DN | 4-6 | 60 | | Antegrade | | Adult | CABG or valve surgery |
|  | BC | 6-8 | 20-25 | | Antegrade | |  |  |
| Gorjipour et al. 2017 | DN | NR | - | | Antegrade | | Children | Corrective surgery of TOF |
|  | St. Thomas | NR | 40 | | Antegrade | |  |  |
| Guajardo et al. 2017 | DN | NR | - | | Antegrade | | Adult | Low-risk, first-time CABG |
|  | BC | NR | 15-20 | | Antegrade | |  |  |
| Gunaydin et al. 2020 | HTK | 4-8 | - | | Antegrade/Retrograde | | Adult | High-risk CABG |
|  | DN | 4-8 | - | | Antegrade/Retrograde | |  |  |
|  | BC | 4-8 | 25 | | Antegrade/Retrograde | |  |  |
| Haan et al. 2020 | HTK | NR | 90-180 | | Antegrade | | Adult | Multiple cardiac surgery |
|  | St. Thomas | NR | 45 | | Antegrade | |  |  |
| Hamad et al. 2017 | DN | NR | - | | Antegrade | | Adult | Primary aortic valve replacement and concomitant CABG |
|  | BC | NR | 15-20 | | Antegrade/Retrograde | |  |  |
| Haranal et al. 2020 | DN | 2-8 | 120 | | NR | | Children | Congenital cardiac surgery |
|  | St. Thomas | NR | 20 | | NR | |  |  |
| Hoyer et al. 2017 | HTK | 5–8 | 90 | | Antegrade/Retrograde | | Adult | Isolated aortic valve replacement |
|  | BC | 15 | 20 | | Antegrade/Retrograde | |  |  |
| Hummel et al. 2016 | HTK | NR | NR | | Antegrade | | Adult | Minimally invasive and open valve surgery |
|  | BC | NR | 20-30 | | Antegrade/Retrograde | |  |  |
| Jakub et al. 2019 | DN | NR | NR | | NR | | Adult | Coronary or heart valve surgery |
|  | BC | NR | NR | | NR | |  |  |
| Khaled et al. 2020 | DN | NR | 90 | | Antegrade | | Adult | Multiple cardiac surgery |
|  | BC | 4 | 15-20 | | Antegrade | |  |  |
| Kim et al. 2018 | DN | NR | 100 | | Antegrade | | Adult | Cardiac valve surgery |
|  | BC | NR | 20-30 | | Antegrade/Retrograde | |  |  |
| Kim et al. 2016 | DN | NR | 90 | | Antegrade/Retrograde | | Adult | Multiple cardiac surgery |
|  | BC | NR | 20 | | Antegrade/Retrograde | |  |  |
| Koda et al. 2021 | DN | 4 | 60 | | Antegrade/Retrograde | | Adult | Multiple cardiac surgery |
|  | BC | 4 | 20 | | Antegrade/Retrograde | |  |  |
| Koeckert et al. 2018 | DN | NR | 60 | | Antegrade/Retrograde | | Adult | Minimally invasive aortic valve replacement |
|  | BC | NR | 20 | | Antegrade/Retrograde | |  |  |
| Kuserli et al. 2020 | DN | 4-8 | 60 | | Antegrade | | Adult | Aortic root surgery |
|  | BC | 4-8 | 20 | | Antegrade | |  |  |
| Lama et al. 2021 | DN | 4-6 | 90 | | Antegrade/Retrograde | | Adult | CABG |
|  | St. Thomas | 4-6 | 20 | | Antegrade/Retrograde | |  |  |
| Lenoir et al. 2021 | DN | 4 | 60 | | Antegrade/Retrograde | | Adult | Aortic root surgery |
|  | BC | 4 | 15-20 | | Antegrade/Retrograde | |  |  |
| Li et al. 2016 | HTK | 4-8 | - | | NR | | Children | Arterial switch operation |
|  | St. Thomas | 4-8 | 30 | | Antegrade | |  |  |
| Luo et al. 2019 | DN | 10 | - | | NR | | Adult | Minimally invasive valve surgery |
|  | St. Thomas | 10 | 20 | | NR | |  |  |
| Marzouk et al. 2020 | DN | 4 | 90 | | Antegrade/Retrograde | | Adult | Multiple cardiac surgery |
|  | BC | 4 | 15-20 | | Antegrade/Retrograde | |  |  |
| Mehrabanian et al. 2018 | DN | NR | 90 | | Antegrade | | Adult | Multiple cardiac surgery |
|  | HTK | 5-8 | 180 | | Antegrade | |  |  |
| Mercan et al. 2020 | HTK | NR | - | | Antegrade | | Adult | Isolated coronary artery bypass surgery |
|  | BC | 4 | 20 | | Antegrade | |  |  |
| Mick et al. 2015 | DN | NR | NR | | Antegrade | | Adult | Primary isolated aortic or mitral valve surgery |
|  | BC | NR | 15-20 | | Antegrade/Retrograde | |  |  |
| Mishra et al. 2016 | DN | 4 | 90 | | Antegrade/Retrograde | | Adult | Elective CABG or DVR surgery |
|  | St. Thomas | 4 | NR | | Antegrade/Retrograde | |  |  |
| Negi et al. 2019 | DN | 4-8 | 75 | | Antegrade | | Children | Intracardiac repair of TOF |
|  | BC | 8-12 | 20 | | Antegrade | |  |  |
| O'Donnell et al. 2019 | BC | 4 | 15-20 | | Antegrade | | Adult | CABG |
|  | DN | 4 | 90 | | Antegrade | |  |  |
| Orak et al. 2020 | DN | NR | NR | | Antegrade | | Adult | Cardiopulmonary bypass surgery |
|  | BC | NR | NR | | Antegrade | |  |  |
| Ota et al. 2015 | DN | 4 | 60-90 | | Antegrade/Retrograde | | Adult | Aortic valve replacement |
|  | BC | 4 | 20 | | Antegrade/Retrograde | |  |  |
| Palo et al. 2017 | HTK | NR | - | | Antegrade | | Adult | Multiple cardiac surgery |
|  | BC | NR | 20 | | Antegrade | |  |  |
| Pizano et al. 2018 | HTK | 4–8 | 180 | | Antegrade | | Adult | Multiple cardiac surgery |
|  | BC | 4–8 | 20-25 | | Antegrade/Retrograde | |  |  |
| Pourmoghadam et al. 2017 | DN | 4-6 | 60-90 | | Antegrade | | Children | Congenital heart surgery |
|  | BC | 4-6 | 20 | | Antegrade | |  |  |
| Pragliola et al. 2020 | DN | 4 | 90 | | Antegrade | | Adult | Multiple cardiac surgery |
|  | BC | 37 | 15 | | Antegrade | |  |  |
| Qulisy et al. 2016 | HTK | 4-8 | NR | | NR | | Children | Congenital cardiac surgery (RACHS-1) |
|  | BC | 10-15 | 20 | | NR | |  |  |
| Rai et al. 2021 | DN | 6-8 | 60-80 | | Antegrade | | Adult | Isolated mitral valve replacement surgery |
|  | BC | 5-8 | 20 | | Antegrade | |  |  |
| Reidy et al. 2021 | DN | NR | NR | | Antegrade | | Adult | CABG |
|  | BC | NR | 15-20 | | Antegrade | |  |  |
| Ross et al. 2021 | DN | 6 | 90 | | Antegrade/Retrograde | | Adult | Multiple cardiac surgery |
|  | BC | 34 | 20-30 | | Antegrade/Retrograde | |  |  |
| Sanetra et al. 2019 | DN | 4 | 90 | | Antegrade | | Adult | Aortic valve replacement |
|  | BC | 4 | 20-30 | | Antegrade | |  |  |
| Sanri et al. 2021 | DN | 4 | 60 | | Antegrade/Retrograde | | Adult | Isolated CABG |
|  | BC | 4 | 15-20 | | Antegrade/Retrograde | |  |  |
| Schutz et al. 2020 | DN | NR | 60-90 | | Antegrade/Retrograde | | Adult | CABG |
|  | BC | 4 | 6-20 | | Antegrade/Retrograde | |  |  |
| Sharma et al. 2021 | DN | 8 | - | | Antegrade | | Adult | DVR |
|  | St. Thomas | 8 | 20 | | Antegrade | |  |  |
| Shi et al. 2020 | DN | 4 | 60 | | Antegrade | | Adult | Aortic root surgery |
|  | BC | 4 | 20-30 | | Antegrade/Retrograde | |  |  |
| Shu et al. 2021 | DN | NR | NR | | NR | | Adult | Multiple cardiac surgery |
|  | St. Thomas | NR | NR | | NR | |  |  |
| Talwar et al. 2019 | DN | 8-10 | NR | | NR | | Children | Intracardiac repair of TOF |
|  | HTK | 4-8 | NR | | NR | |  |  |
| Timek et al. 2016 | DN | 4 | 60 | | Antegrade | | Adult | CABG |
|  | BC | 5 | 15-20 | | Antegrade/Retrograde | |  |  |
| Timek et al. 2019 | DN | 4 | 60 | | Antegrade | | Adult | Isolated CABG |
|  | BC | 4 | 15-20 | | Antegrade/Retrograde | |  |  |
| Ucak et al. 2019 | DN | 4 | 60 | | Antegrade | | Adult | CABG |
|  | BC | 33-34 | 15-20 | | Antegrade/Retrograde | |  |  |
| Ucak et al. 2021 | DN | 4 | - | | Antegrade | | Adult | Aortic valve replacement surgery |
|  | BC | 24-28 | 15-20 | | Antegrade | |  |  |
| Vaidya et al. 2020 | DN | NR | 90 | | Antegrade | | Adult | Open-heart surgery |
|  | St. Thomas | NR | 25 | | Antegrade | |  |  |
| Valente et al. 2019 | HTK | 5 | - | | Antegrade | | Children | Correction surgery of cyanogenic congenital cardiopathies |
|  | BC | 20 | 30 | | Antegrade | |  |  |
| Viana et al. 2013 | HTK | 4 | 180 | | Antegrade/Retrograde | | Adult | Multiple cardiac surgery |
|  | BC | 28 | 15-20 | | Antegrade/Retrograde | |  |  |
| Vistarini et al. 2017 | DN | NR | - | | Antegrade | | Adult | Minimally invasive aortic valve surgery |
|  | BC | NR | 15-20 | | Antegrade/Retrograde | |  |  |
| Vivacqua et al. 2019 | HTK | 4-5 | - | | Antegrade | | Adult | Multiple cardiac surgery |
|  | BC | 4-8 | 20 | | Antegrade | |  |  |
| Yerebakan et al. 2014 | DN | 4 | 90 | | Antegrade/Retrograde | | Adult | High-risk CABG after acute myocardial infarction |
|  | BC | 5 | 20 | | Antegrade/Retrograde | |  |  |
| Ziazadeh et al. 2017 | DN | 4 | 60 | | Antegrade | | Adult | Minimally invasive aortic valve surgery |
|  | BC | 4 | 15-20 | | Antegrade/Retrograde | |  |  |

-, A single dose of cardioplegic solution was given, or patients who received additional cardioplegic solution were excluded from the study; BC, blood cardioplegia; CABG, coronary artery bypass grafting; CHD, congenital heart disease; DN, del Nido cardioplegia; DVR, double valve replacement; HTK, histidine-tryptophan-ketoglutarate cardioplegia; NR, not reported; RACHS, Risk Adjustment in Congenital Heart Surgery; TOF, tetralogy of Fallot.

Supplementary Table 6. Summary of risk of bias in the included randomized controlled trials.

| Study | Selection bias | | Performance bias | Detection bias | Attrition bias | Reporting bias | Other bias |
| --- | --- | --- | --- | --- | --- | --- | --- |
|  | Random sequence generation | Allocation concealment | Blinding of participants  and personnel | Blinding of outcome  assessment | Incomplete outcome data | Selective reporting |  |
| Ad et al. 2017 | Low | Low | Low | Low | Unclear | Unclear | Unclear |
| Ali et al. 2021 | Low | Low | Low | Low | Low | Low | Unclear |
| Bigdelian et al. 2020 | Low | Low | Low | Low | Low | Low | Unclear |
| Braathen et al. 2011 | Low | Low | Low | Low | Low | Unclear | Unclear |
| Busro et al. 2018 | Low | Low | Low | Low | Low | High | Unclear |
| Cvetković et al. 2021 | Low | Low | Low | Low | Low | Low | Unclear |
| Gaudino et al. 2013 | Low | Unclear | Low | Low | Low | Low | Unclear |
| Gorjipour et al. 2017 | Low | Low | Low | Low | Low | High | Unclear |
| Haranal et al. 2020 | Low | Low | Low | Low | Low | Low | Low |
| Lama et al. 2021 | Low | Low | Low | Low | Low | Low | Unclear |
| Mehrabanian et al. 2018 | Low | Unclear | Low | Low | Low | Low | Unclear |
| Mercan et al. 2020 | Low | Low | Low | Low | Low | Low | Unclear |
| Negi et al. 2019 | Unclear | Unclear | Unclear | Low | Low | Unclear | Unclear |
| Sanetra et al. 2019 | Low | Low | Low | Low | Low | Low | Unclear |
| Talwar et al. 2019 | Low | Low | Low | Low | Low | Low | Unclear |
| Ucak et al. 2019 | Unclear | Low | Unclear | Low | Low | Low | Unclear |
| Valente et al. 2019 | Unclear | Low | Unclear | Low | Low | Low | Unclear |
| Vivacqua et al. 2019 | Low | Low | Unclear | Low | Low | Low | Unclear |

Supplementary Table 7. Quality of the observational cohort studies, based on the Newcastle–Ottawa scale.

| Study | Selection | | | | Comparability | Outcome | | | Score |
| --- | --- | --- | --- | --- | --- | --- | --- | --- | --- |
|  | I | II | III | IV | V | VI | VII | VIII |  |
| Arafat et al. 2021 | * | * | * | * | * | * | * |  | 7 |
| Caneo et al. 2021 | * | * | * | * | ** | * |  |  | 7 |
| Cayir et al. 2020 | * | * | * | * | ** | * |  |  | 7 |
| Chen et al. 2021 | * | * | * | * | ** | * |  |  | 7 |
| Elassal et al. 2020 | * | * | * | * | ** | * | * | * | 9 |
| George et al. 2020 | * | * | * | * | ** | * | * |  | 8 |
| Guajardo et al. 2017 | * | * | * | * | ** | * |  |  | 7 |
| Gunaydin et al. 2020 | * | * | * | * | ** | * | * |  | 8 |
| Haan et al. 2020 | * | * | * | * | ** | * | * |  | 8 |
| Hamad et al. 2017 | * | * |  | * | * | * | * |  | 6 |
| Hoyer et al. 2017 | * | * | * | * | * | * | * |  | 7 |
| Hummel et al. 2016 | * | * |  | * | ** | * |  |  | 6 |
| Jakub et al. 2019 | * | * |  | * | ** | * |  |  | 6 |
| Khaled et al. 2020 | * | * | * | * | ** | * | * |  | 8 |
| Kim et al. 2018 | * | * | * | * | * | * |  |  | 6 |
| Kim et al. 2016 | * | * | * | * | ** | * |  |  | 7 |
| Koda et al. 2021 | * | * | * | * | * | * |  |  | 6 |
| Koeckert et al. 2018 | * | * | * | * | * | * |  |  | 7 |
| Kuserli et al. 2020 | * | * | * | * | * | * | * |  | 7 |
| Lenoir et al. 2021 | * | * | * | * | * | * | * |  | 7 |
| Li et al. 2016 | * | * | * | * | * | * |  |  | 6 |
| Luo et al. 2019 | * | * | * | * | * |  |  |  | 6 |
| Marzouk et al. 2020 | * | * | * | * | * | * |  |  | 9 |
| Mick et al. 2015 | * | * |  | * | ** | * |  |  | 7 |
| Mishra et al. 2016 | * | * | * | * | ** | * |  |  | 6 |
| O'Donnell et al. 2019 | * | * | * | * | ** | * | * | * | 7 |
| Orak et al. 2020 | * | * | * | * | * | * | * |  | 7 |
| Ota et al. 2016 | * | * | * | * | ** | * |  |  | 7 |
| Palo et al. 2017 | * | * | * | * | * | * |  |  | 9 |
| Pizano et al. 2018 | * | * | * | * | * | * | * |  | 6 |
| Pourmoghadam et al. 2017 | * | * | * | * | ** | * | * | * | 7 |
| Pragliola et al. 2020 | * | * | * | * | * | * |  |  | 7 |
| Qulisy et al. 2016 | * | * |  | * | ** | * | * |  | 6 |
| Rai et al. 2021 | * | * | * | * | * | * | * |  | 6 |
| Reidy et al. 2021 | * | * |  | * | * | * |  |  | 8 |
| Ross et al. 2021 | * | * | * | * | * | * |  |  | 6 |
| Sanri et al. 2021 | * | * | * | * | * | * |  |  | 6 |
| Schutz et al. 2020 | * | * | * | * | ** | * | * | * | 9 |
| Sharma et al. 2021 | * | * | * | * | * | * |  |  | 7 |
| Shi et al. 2020 | * | * | * | * | ** | * | * |  | 6 |
| Shu et al. 2021 | * | * | * | * | * | * |  |  | 6 |
| Timek et al. 2016 | * | * | * | * | * | * |  |  | 6 |
| Timek et al. 2019 | * | * | * | * | * | * |  |  | 6 |
| Ucak et al. 2021 | * | * | * | * | * | * | * |  | 7 |
| Vaidya et al. 2020 | * | * | * | * | * | * | * | * | 8 |
| Viana et al. 2013 | * | * | * | * | * | * |  |  | 6 |
| Vistarini et al. 2017 | * | * | * | * | * | * | * |  | 6 |
| Yerebakan et al. 2014 | * | * | * | * | ** | * | * | * | 9 |
| Ziazadeh et al. 2017 | * | * | * | * | * | * | * | * | 8 |

I, Representativeness of the exposed cohort; II, Selection of the non-exposed cohort; III, Ascertainment of exposure; IV Demonstration that the outcome of interest was not present at start of study; V, Comparability of cohorts on the basis of the design or analysis; VI, Assessment of outcome; VII, Was follow-up long enough for outcomes to occur?; VIII, Adequacy of cohort follow-up.

Supplementary Table 8. Node-splitting analysis of the network meta-analysis in order to assess consistency between direct and indirect evidence.

| Patient type | Outcome | Comparison | K | Prop | Direct | Indirect | Difference | P-value |
| --- | --- | --- | --- | --- | --- | --- | --- | --- |
| Adults | Perioperative mortality | DN vs BC | 21 | 0.97 | -0.08 | -0.18 | 0.11 | 0.9 |
|  |  | DN vs HTK | 1 | 0.02 | 1.03 | -0.33 | 1.36 | 0.41 |
|  |  | DN vs St. Thomas | 5 | 0.51 | -0.53 | -0.18 | -0.35 | 0.7 |
|  |  | HTK vs BC | 6 | 0.97 | 0.22 | 0.44 | -0.22 | 0.8 |
|  |  | HTK vs St. Thomas | 1 | 0.55 | 0.11 | -0.25 | 0.35 | 0.7 |
|  |  | St. Thomas vs BC | 0 | 0 | - | 0.28 | - | - |
|  | Atrial fibrillation | DN vs BC | 25 | 0.98 | 0.09 | 0.1 | -0.01 | 0.98 |
|  |  | DN vs HTK | 1 | 0.04 | 0.63 | 0.4 | 0.23 | 0.71 |
|  |  | DN vs St. Thomas | 3 | 0.88 | 0.08 | 0.09 | -0.01 | 0.97 |
|  |  | HTK vs BC | 4 | 0.91 | -0.31 | -0.38 | 0.07 | 0.86 |
|  |  | HTK vs St. Thomas | 1 | 0.21 | -0.32 | -0.33 | 0.01 | 0.97 |
|  |  | St. Thomas vs BC | 0 | 0 | - | 0.01 | - | - |
|  | Renal failure | DN vs BC | 17 | 1 | -0.13 | - | - | - |
|  |  | DN vs HTK | 0 | 0 | - | -0.11 | - | - |
|  |  | DN vs St. Thomas | 1 | 1 | -0.41 | - | - | - |
|  |  | HTK vs BC | 5 | 1 | -0.02 | - | - | - |
|  |  | HTK vs St. Thomas | 0 | 0 | - | -0.3 | - | - |
|  |  | St. Thomas vs BC | 0 | 0 | - | 0.27 | - | - |
|  | Stroke | DN vs BC | 21 | 1 | 0.16 | - | - | - |
|  |  | DN vs HTK | 0 | 0 | - | -0.04 | - | - |
|  |  | DN vs St. Thomas | 1 | 1 | -1.1 | - | - | - |
|  |  | HTK vs BC | 3 | 1 | 0.2 | - | - | - |
|  |  | HTK vs St. Thomas | 0 | 0 | - | -1.06 | - | - |
|  |  | St. Thomas vs BC | 0 | 0 | - | 1.26 | - | - |
|  | IABP | DN vs BC | 9 | 0.96 | 0.02 | -0.4 | 0.42 | 0.55 |
|  |  | DN vs HTK | 1 | 0.16 | 0.4 | 0.1 | 0.3 | 0.61 |
|  |  | DN vs St. Thomas | 2 | 0.66 | -0.35 | 0.66 | -1.01 | 0.34 |
|  |  | HTK vs BC | 5 | 0.94 | -0.14 | -0.2 | 0.06 | 0.94 |
|  |  | HTK vs St. Thomas | 1 | 0.38 | 0.46 | -0.54 | 1.01 | 0.34 |
|  |  | St. Thomas vs BC | 0 | 0 | - | 0.01 | - | - |
|  | Re-exploration | DN vs BC | 12 | 0.94 | -0.06 | -0.08 | 0.02 | 0.99 |
|  |  | DN vs HTK | 0 | 0 | - | -0.07 | - | - |
|  |  | DN vs St. Thomas | 1 | 0.22 | 0 | 0.02 | -0.02 | 0.99 |
|  |  | HTK vs BC | 3 | 0.98 | 0.01 | 0.02 | -0.02 | 0.99 |
|  |  | HTK vs St. Thomas | 2 | 0.87 | 0.09 | 0.07 | 0.02 | 0.99 |
|  |  | St. Thomas vs BC | 0 | 0 | - | -0.08 | - | - |
|  | ICU stay | DN vs BC | 23 | 0.93 | 2.93 | -1.11 | 4.03 | 0.44 |
|  |  | DN vs HTK | 2 | 0.39 | 4.12 | 1.96 | 2.16 | 0.59 |
|  |  | DN vs St. Thomas | 5 | 0.98 | -2.68 | -11.73 | 9.05 | 0.58 |
|  |  | HTK vs BC | 7 | 0.86 | -0.1 | -0.4 | 0.3 | 0.95 |
|  |  | HTK vs St. Thomas | 1 | 0.03 | -14.4 | -5.35 | -9.05 | 0.58 |
|  |  | St. Thomas vs BC | 0 | 0 | - | 5.48 | - | - |
|  | Hospital stay | DN vs BC | 22 | 0.98 | 0.16 | 0.62 | -0.46 | 0.61 |
|  |  | DN vs HTK | 1 | 0.41 | 0.6 | 0.46 | 0.14 | 0.77 |
|  |  | DN vs St. Thomas | 4 | 1 | 0.11 | - | - | - |
|  |  | HTK vs BC | 5 | 0.9 | -0.34 | -0.35 | 0.01 | 0.99 |
|  |  | HTK vs St. Thomas | 0 | 0 | - | -0.41 | - | - |
|  |  | St. Thomas vs BC | 0 | 0 | - | 0.06 | - | - |
| Children | Perioperative mortality | DN vs BC | 2 | 0.57 | -0.97 | 0.98 | -1.95 | 0.17 |
|  |  | DN vs HTK | 1 | 0.22 | 0 | 0.28 | -0.28 | 0.87 |
|  |  | DN vs St. Thomas | 2 | 0.86 | -0.26 | -2.38 | 2.12 | 0.13 |
|  |  | HTK vs BC | 3 | 0.81 | 0.01 | -1.94 | 1.95 | 0.17 |
|  |  | HTK vs St. Thomas | 1 | 0.53 | -1.75 | 0.37 | -2.12 | 0.13 |
|  |  | St. Thomas vs BC | 0 | 0 | - | 0.4 | - | - |
|  | ICU stay | DN vs BC | 1 | 0.51 | 9.6 | 126.94 | -117.34 | 0.29 |
|  |  | DN vs HTK | 1 | 0.42 | -4.75 | 123.88 | -128.63 | 0.19 |
|  |  | DN vs St. Thomas | 5 | 0.86 | -1.59 | -251.49 | 249.89 | 0.02 |
|  |  | HTK vs St. Thomas | 1 | 0.45 | -242.4 | 7.49 | -249.89 | 0.02 |
|  |  | HTK vs BC | 2 | 0.75 | 25.49 | -91.85 | 117.34 | 0.29 |
|  |  | St. Thomas vs BC | 0 | 0 | - | 102.18 | - | - |
|  | Hospital stay | DN vs BC | 2 | 0.65 | -0.46 | 4.16 | -4.62 | 0.53 |
|  |  | DN vs HTK | 1 | 0.4 | -0.76 | 4.86 | -5.62 | 0.42 |
|  |  | DN vs St. Thomas | 3 | 0.77 | 0.08 | -12.11 | 12.19 | 0.12 |
|  |  | HTK vs St. Thomas | 1 | 0.5 | -11.5 | 0.69 | -12.19 | 0.12 |
|  |  | HTK vs BC | 2 | 0.69 | -0.03 | -4.65 | 4.62 | 0.53 |
|  |  | St. Thomas vs BC | 0 | 0 | - | 3.93 | - | - |

K, Number of studies providing direct evidence; Prop, Proportion of direct evidence; Direct, Estimated treatment effect (RR/MD) derived from direct evidence; Indirect, Estimated treatment effect (RR/MD) derived from indirect evidence; Difference, difference in the network (direct versus indirect); P-value, p-value of test for disagreement (direct versus indirect). P＜0.05 was considered to indicate inconsistency and was shown in bold. BC, blood cardioplegia; DN, del Nido cardioplegia; HTK, histidine-tryptophan-ketoglutarate cardioplegia; IABP, intra-aortic balloon pump; ICU, intensive care unit.


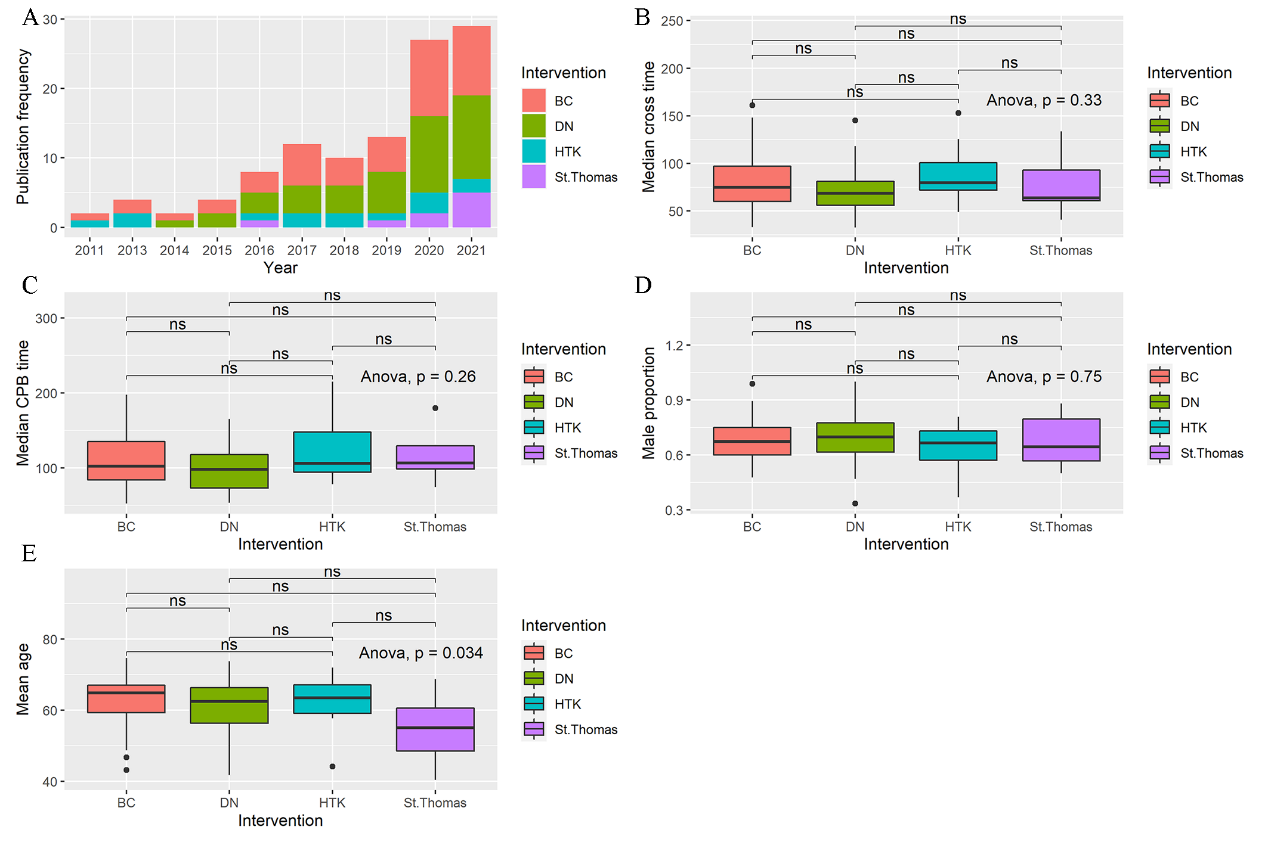


Figure S1. Transitivity analysis of each cardioplegia type across all adult patients.


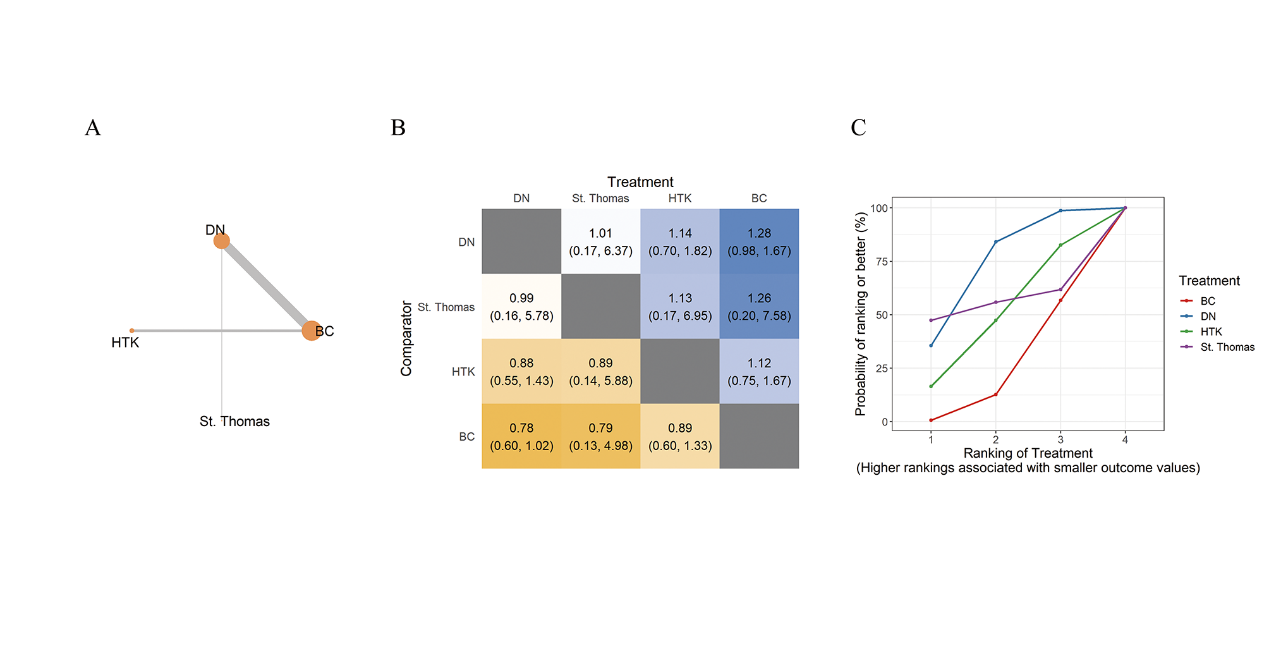


Figure S2. NMA of renal failure across all adult trials.


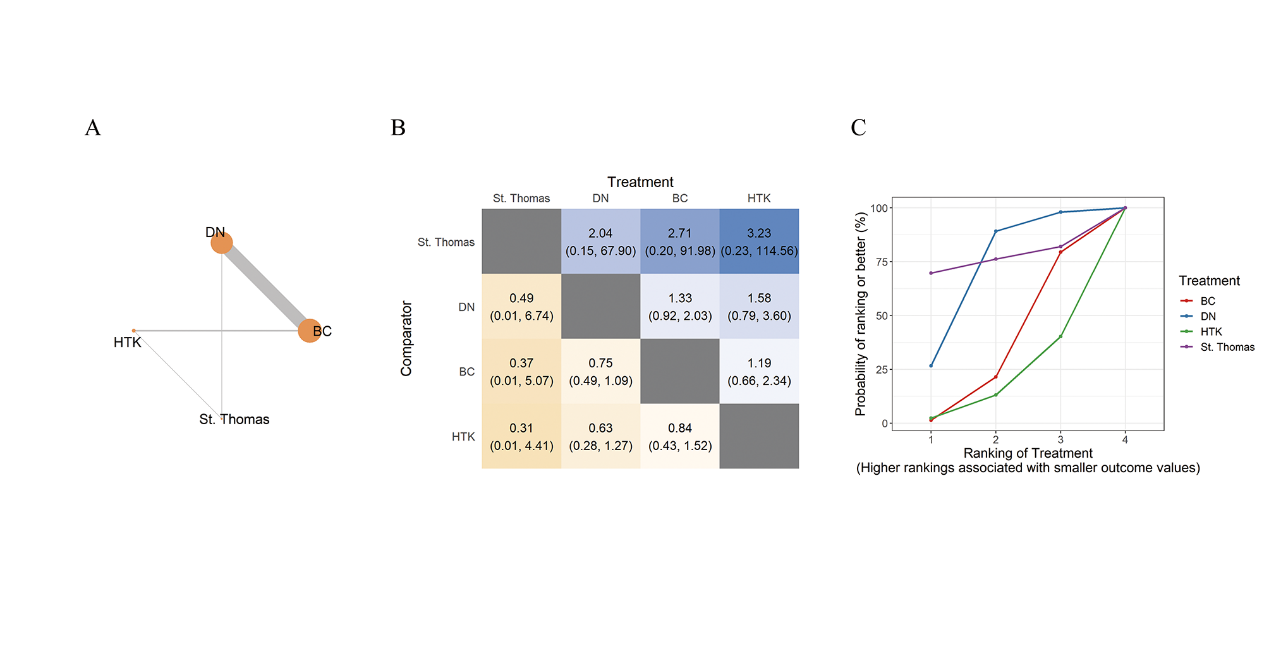


Figure S3. NMA of stroke across all adult trials.


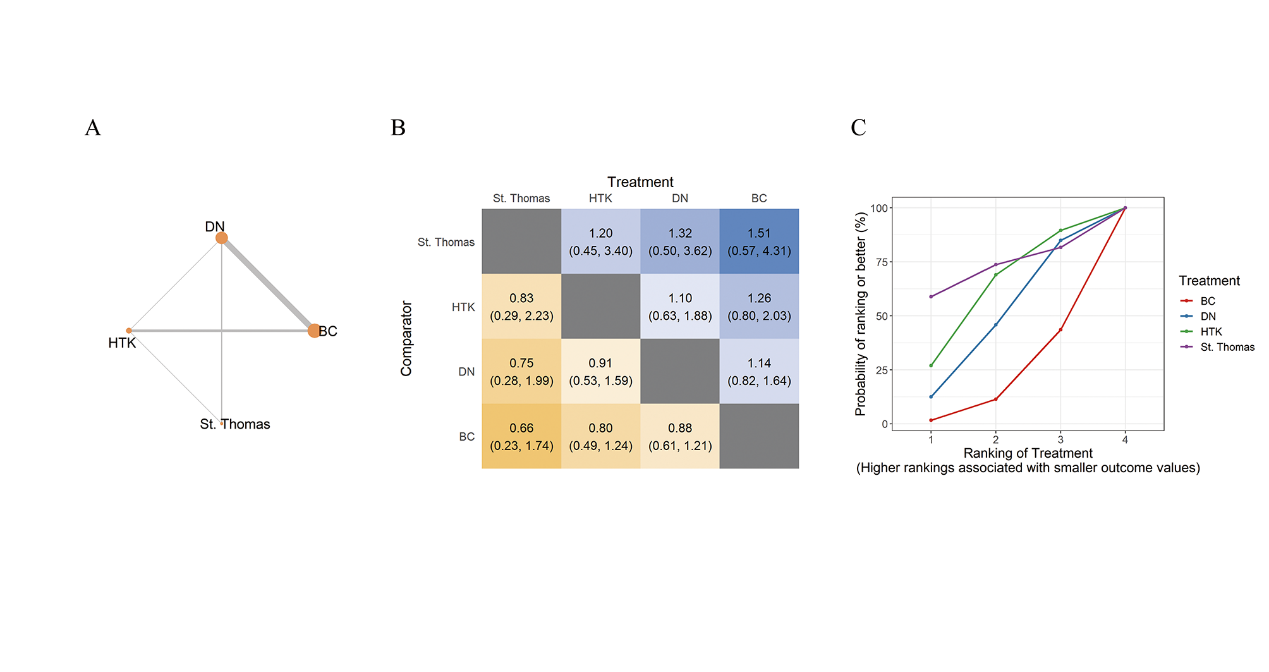


Figure S4. NMA of the use of intra-aortic balloon pump across all adult trials.


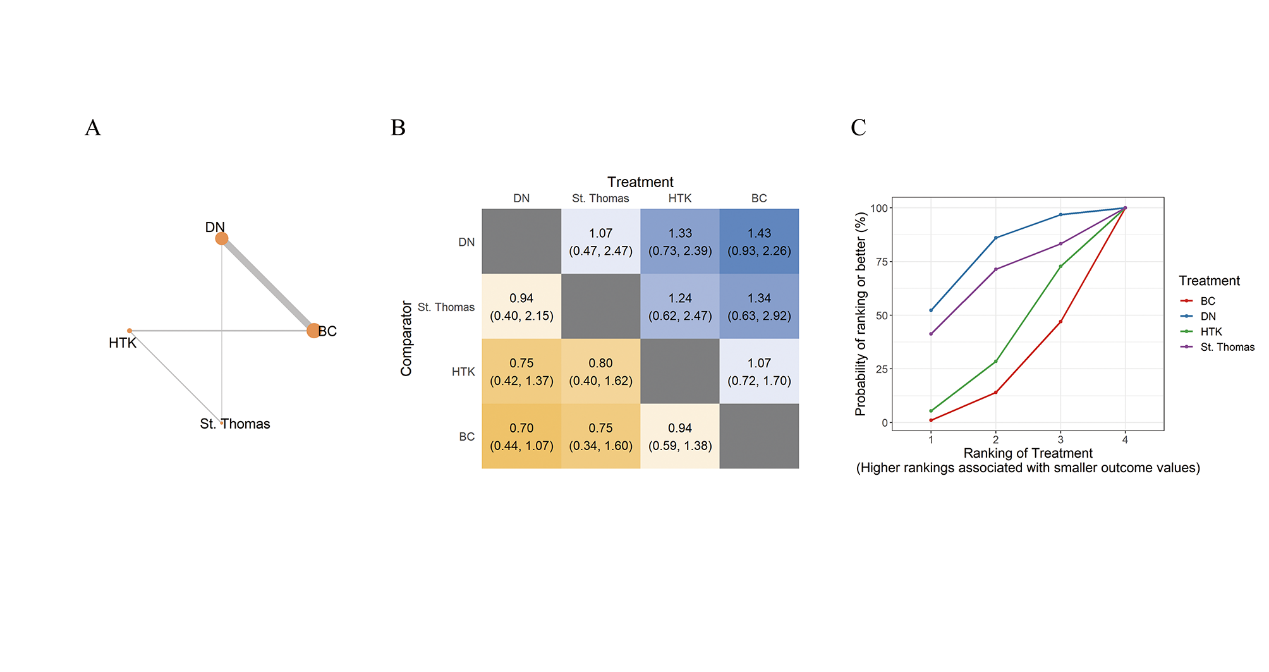


Figure S5. NMA of re-exploration across all adult trials.


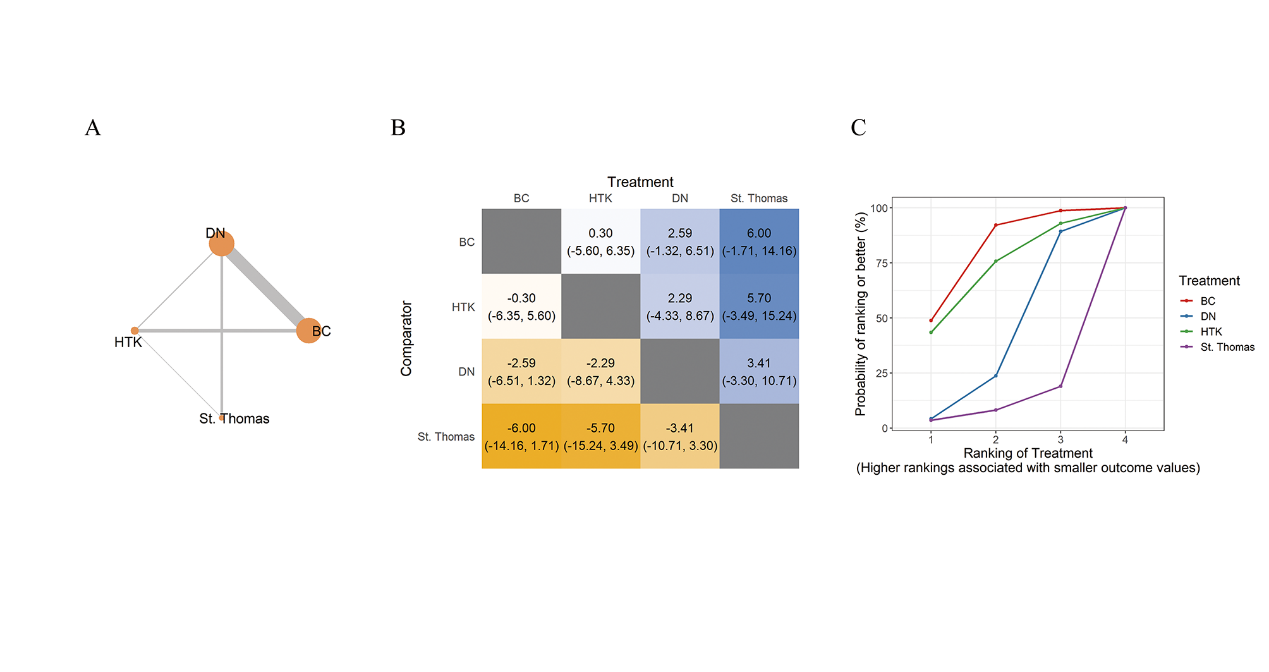


Figure S6. NMA of ICU stay across all adult trials.


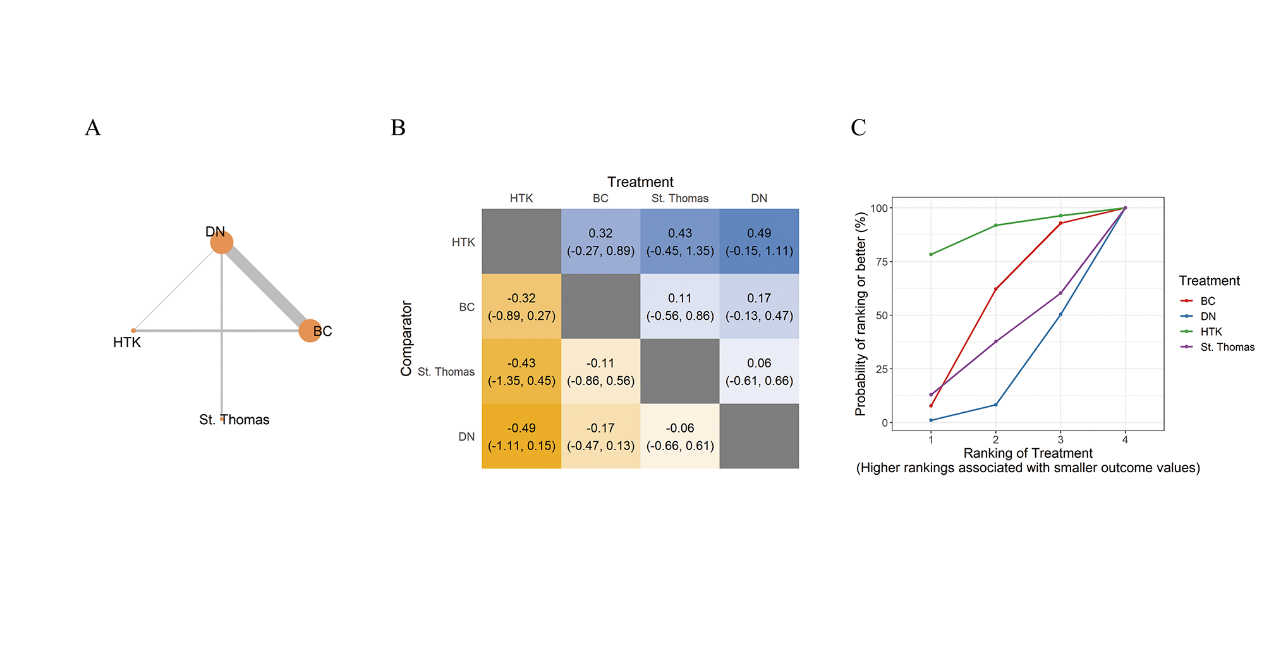


Figure S7. NMA of hospital stay across all adult trials.


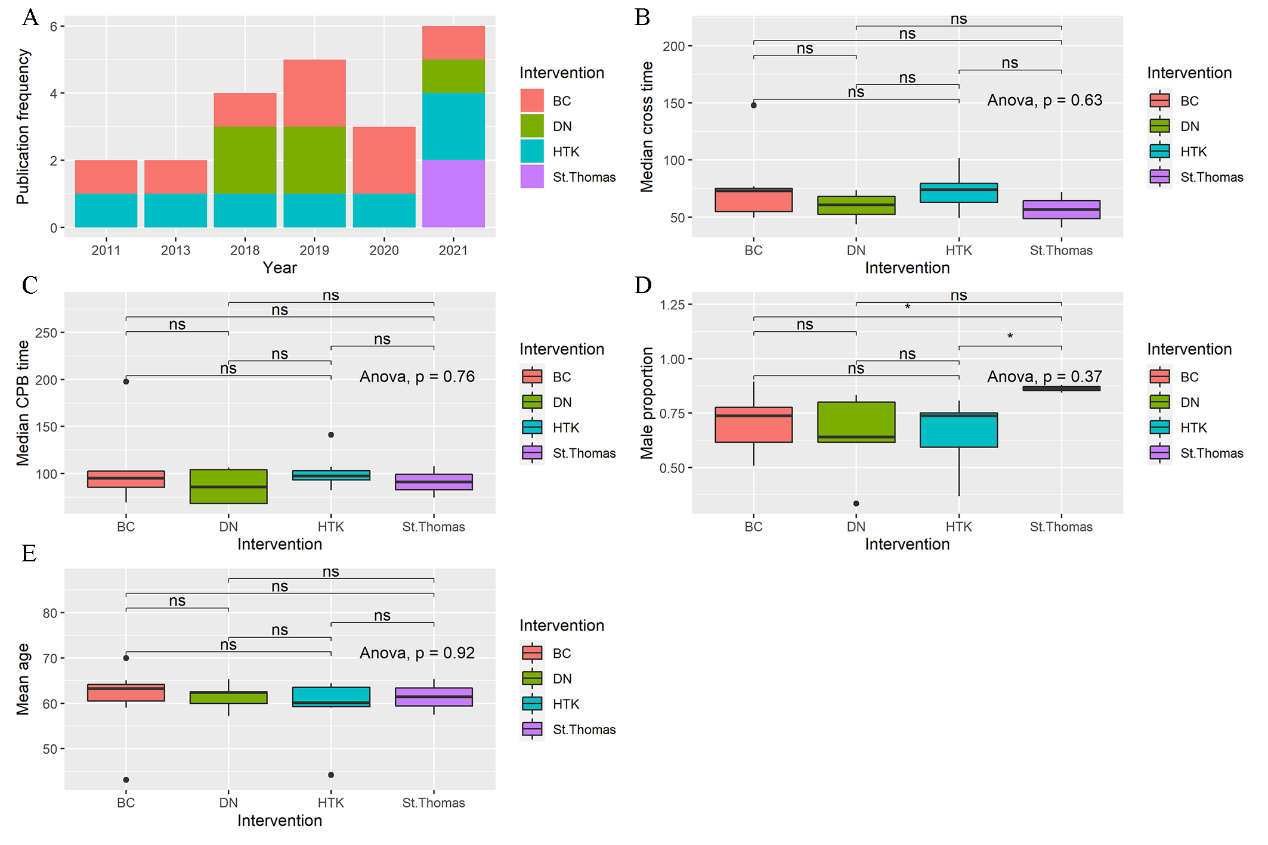


Figure S8. Transitivity analysis of each cardioplegia type across adult patients only in RCTs.


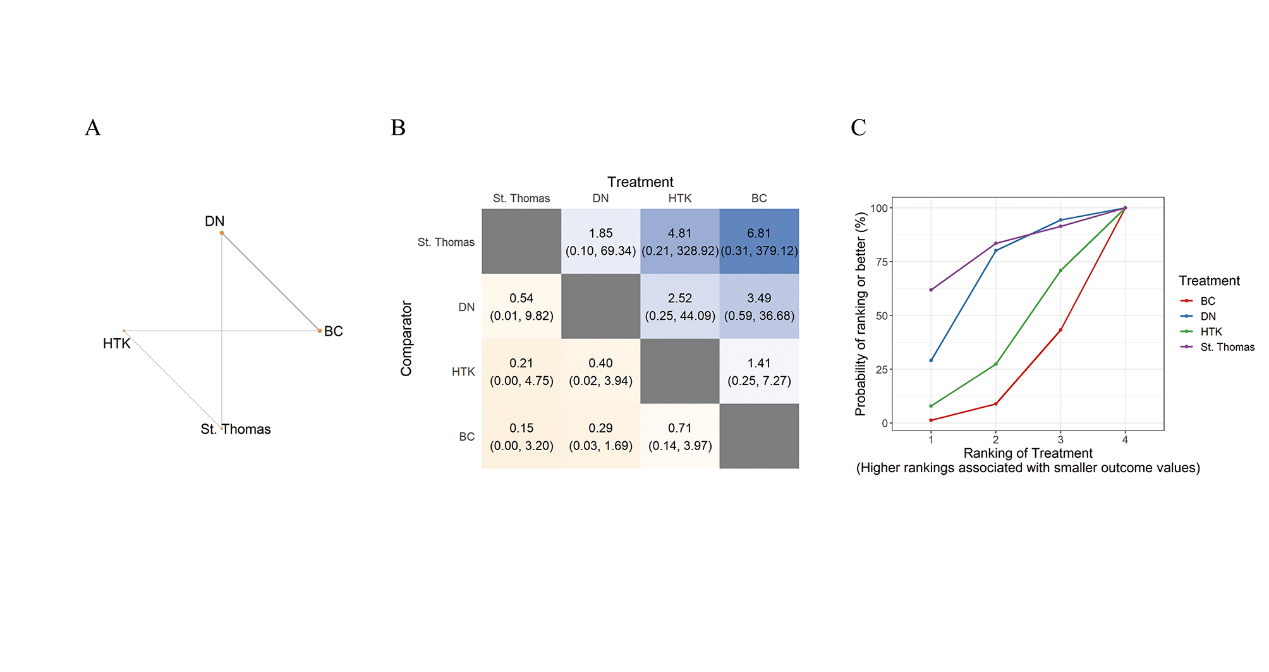


Figure S9. NMA of perioperative mortality across adult patients only in RCTs.


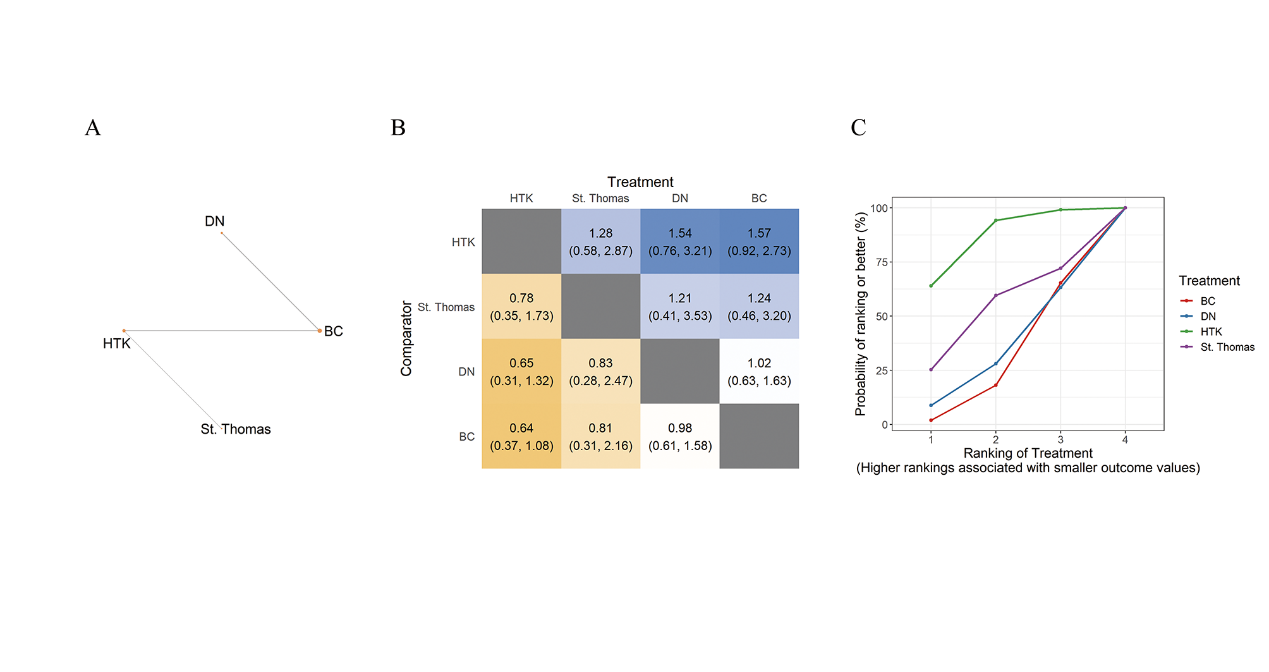


Figure S10. NMA of atrial fibrillation across adult patients only in RCTs.


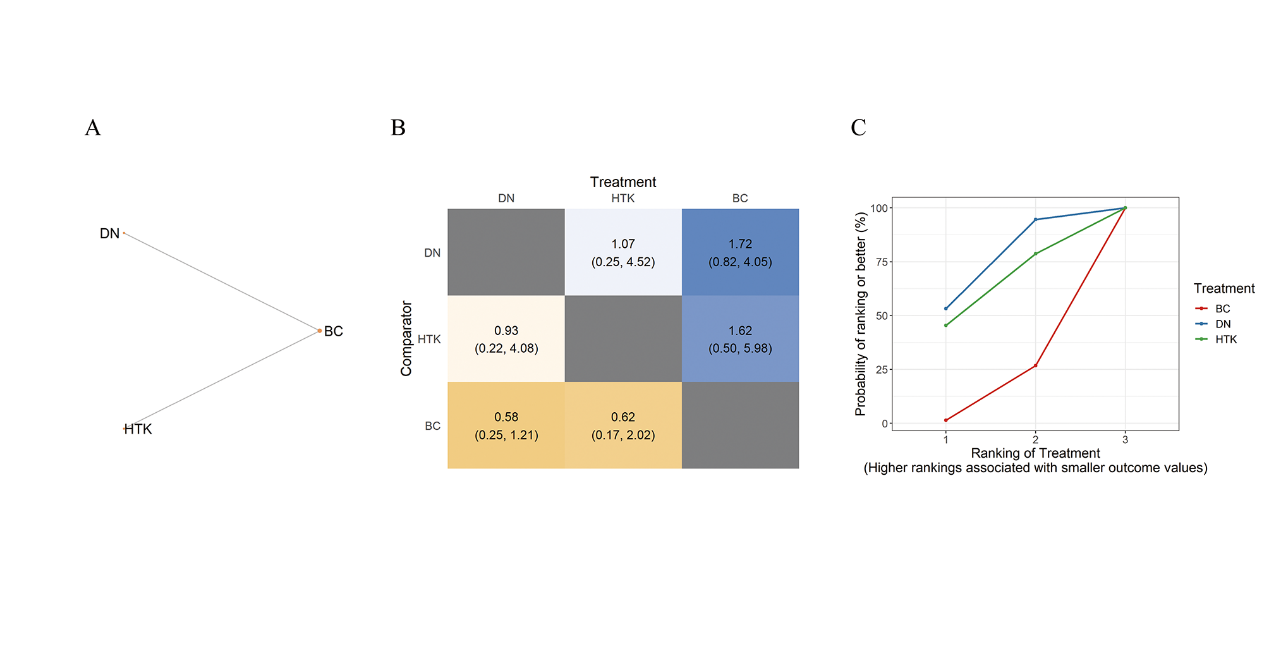


Figure S11. NMA of renal failure across adult patients only in RCTs.


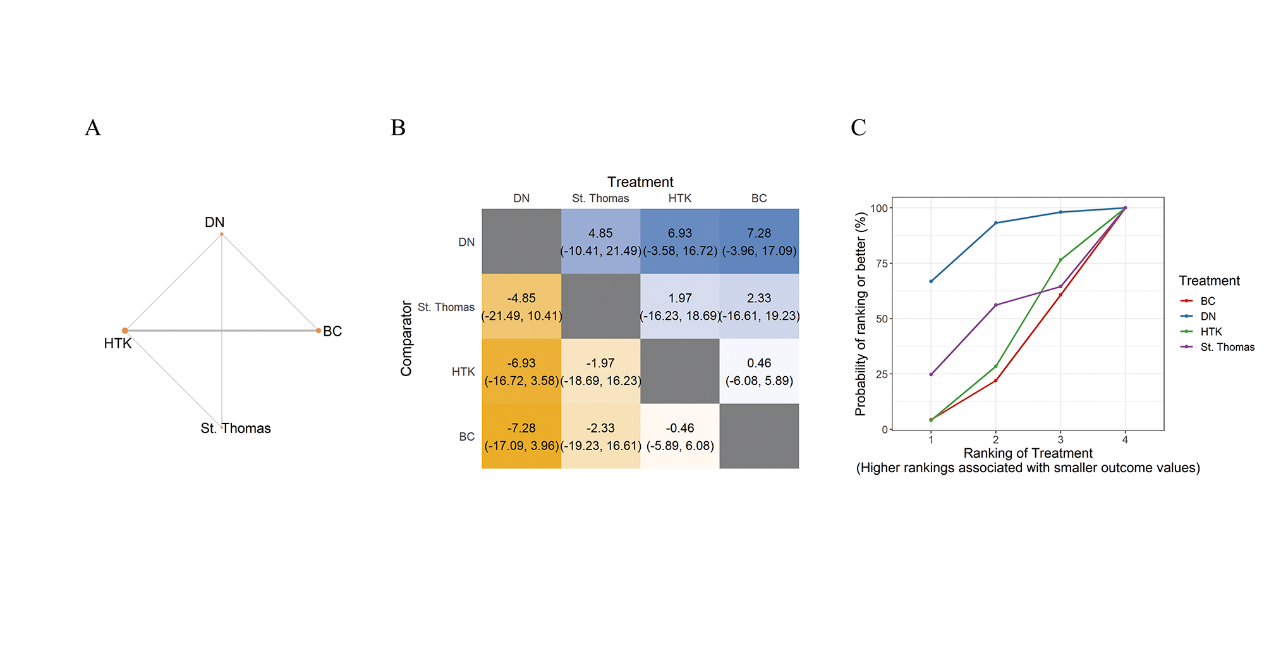


Figure S12. NMA of ICU stay across adult patients only in RCTs.


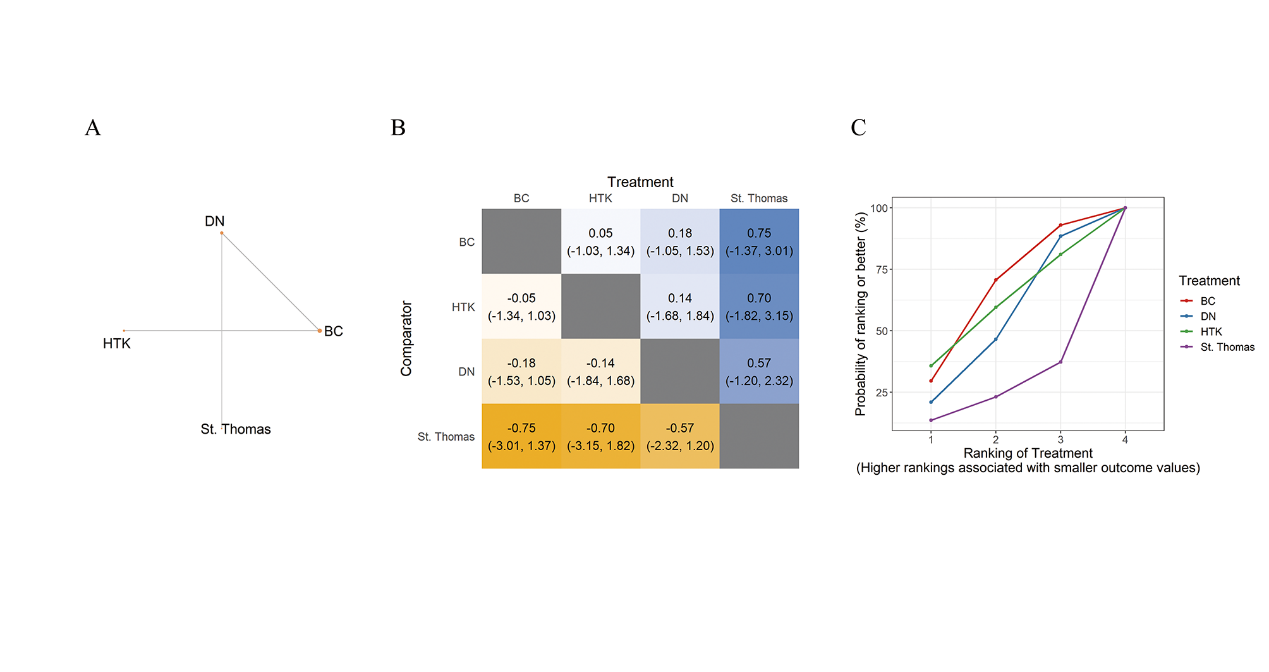


Figure S13. NMA of hospital stay across adult patients only in RCTs.


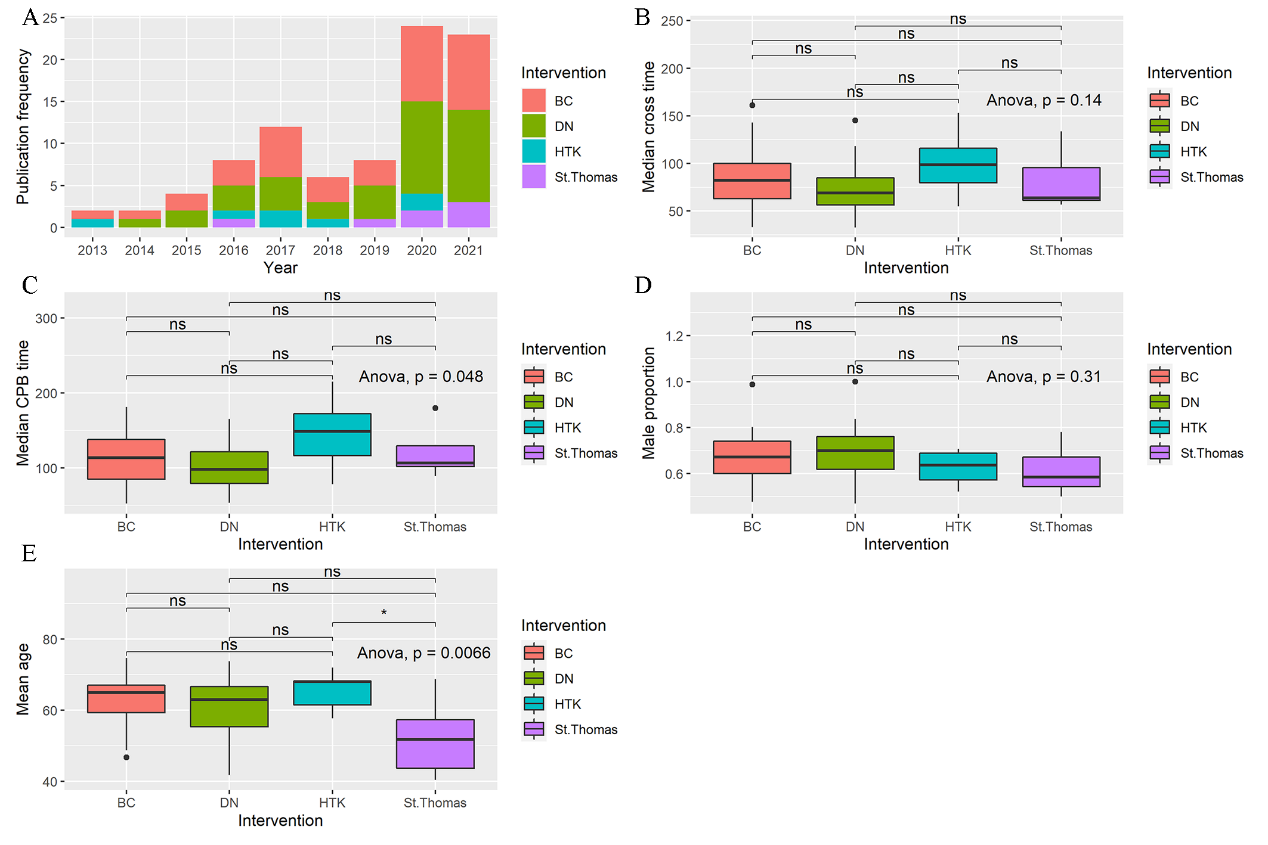


Figure S14. Transitivity analysis of each cardioplegia type across adult patients only in cohort studies.


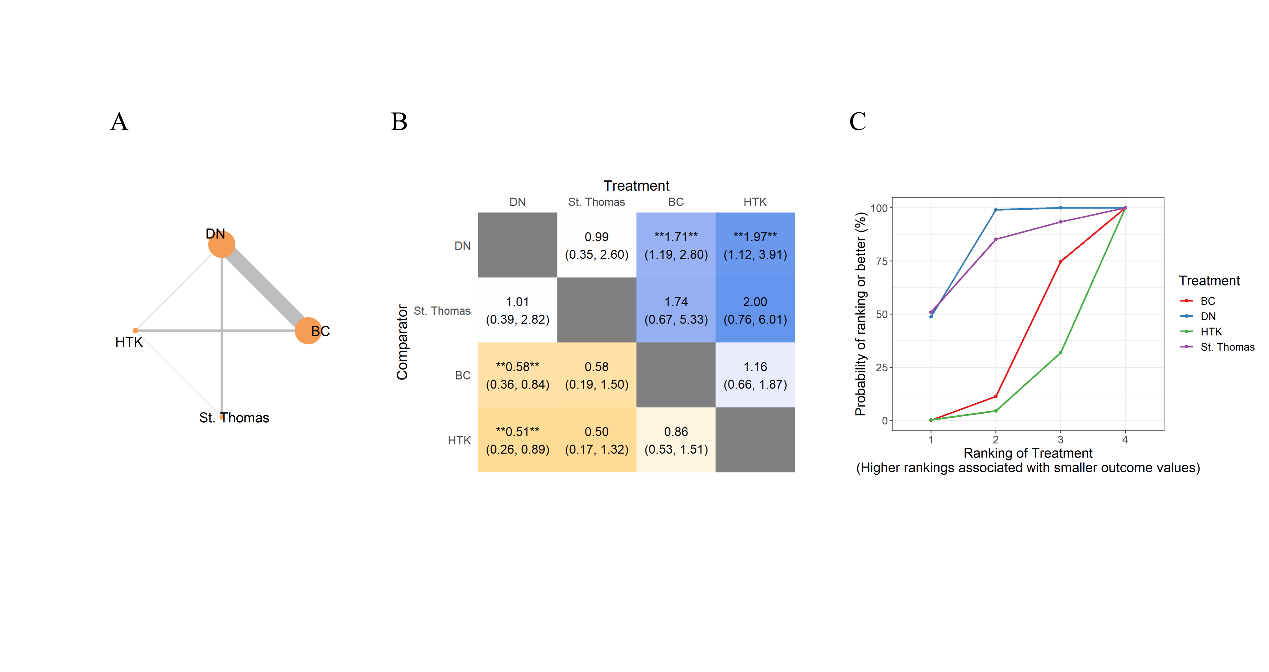


Figure S15. NMA of perioperative mortality across adult patients only in cohort studies.


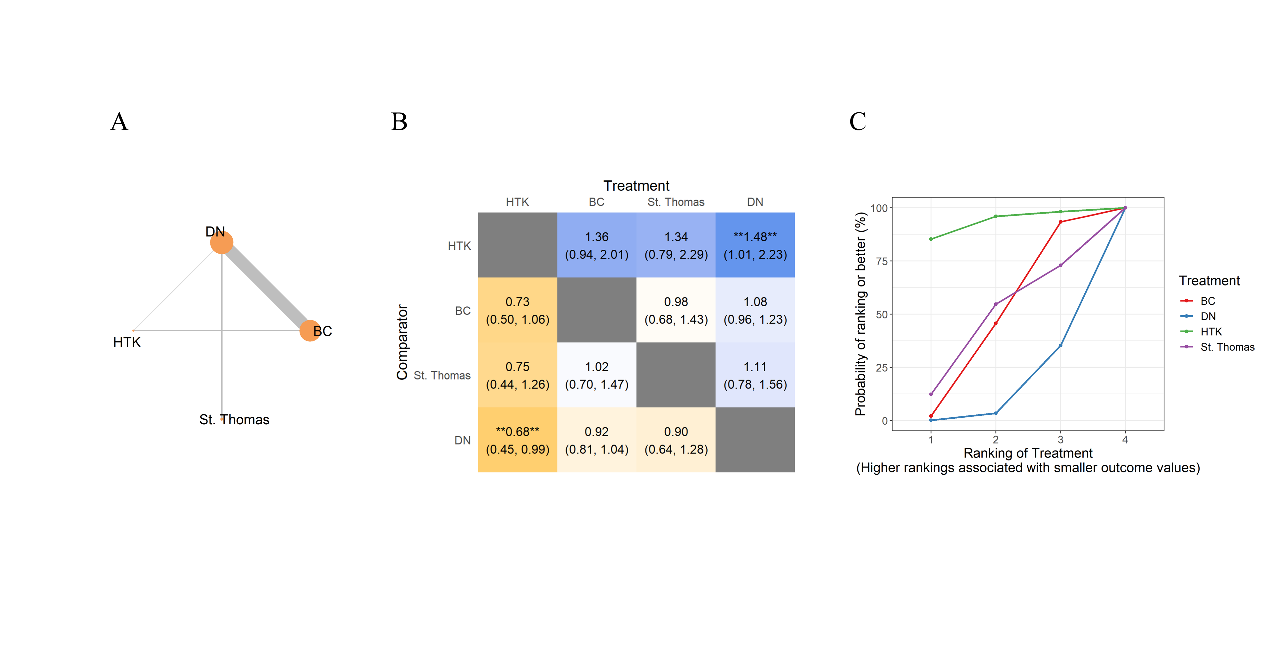


Figure S16. NMA of atrial fibrillation across adult patients only in cohort studies.


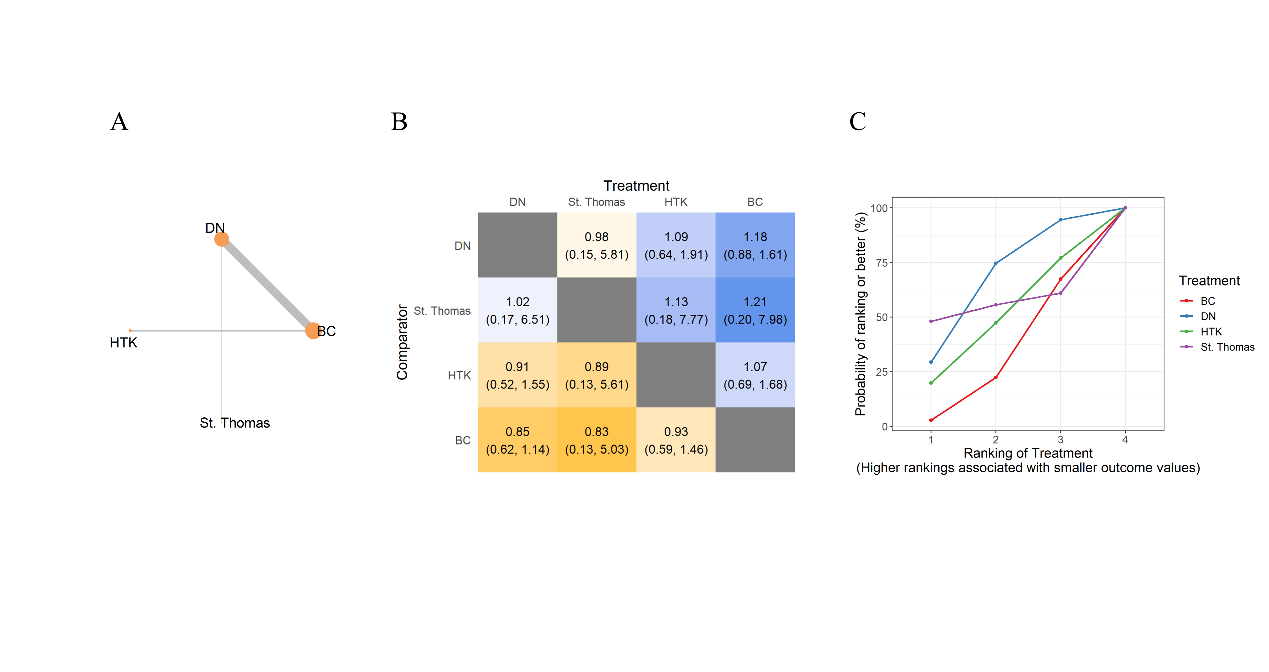


Figure S17. NMA of renal failure across adult patients only in cohort studies.


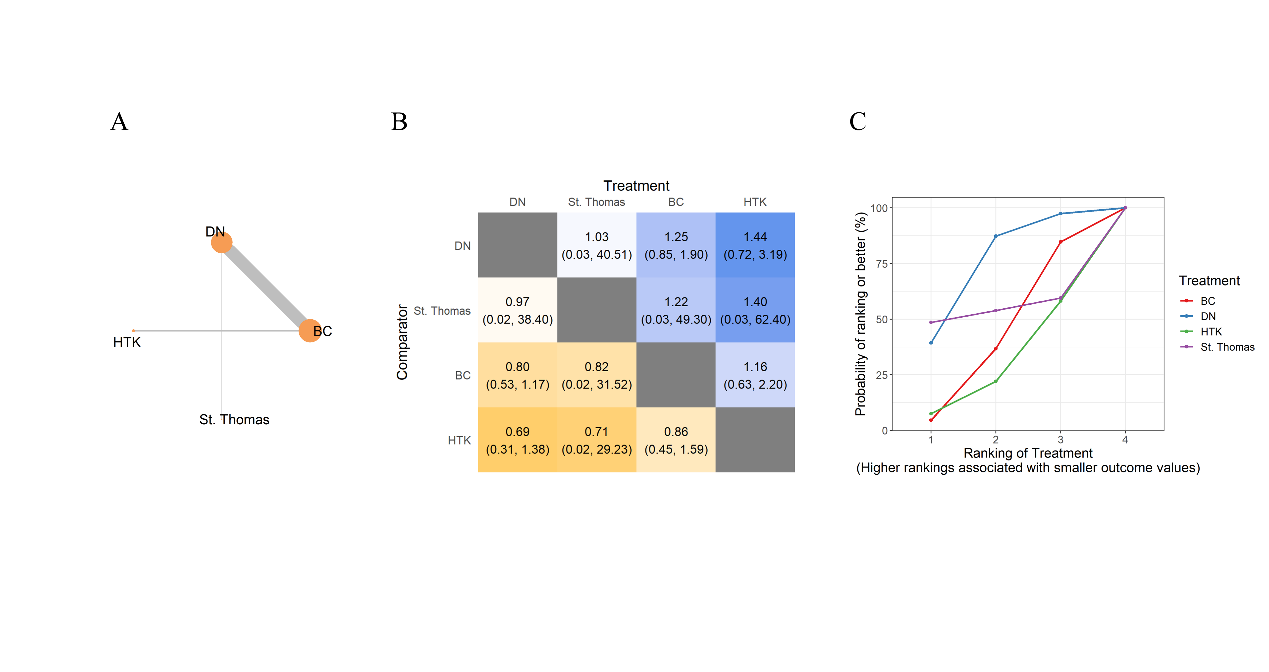


Figure S18. NMA of stroke across adult patients only in cohort studies.


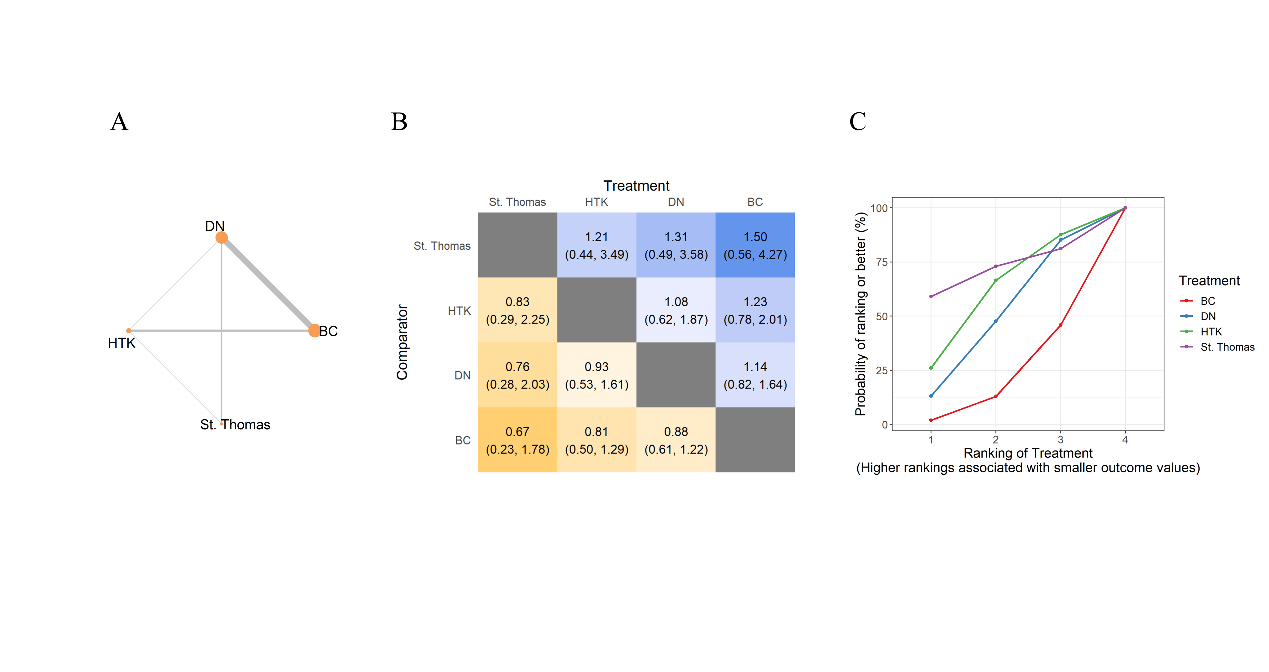


Figure S19. NMA of the use of intra-aortic balloon pump across adult patients only in cohort studies.


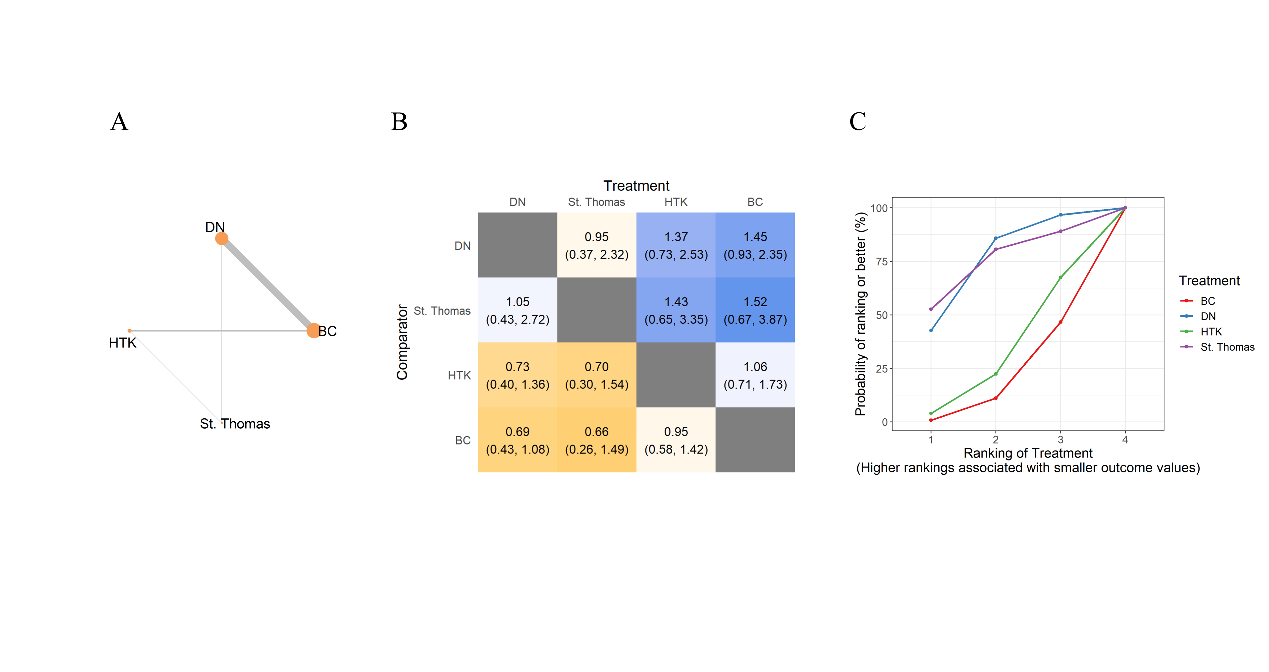


Figure S20. NMA of re-exploration across adult patients only in cohort studies.


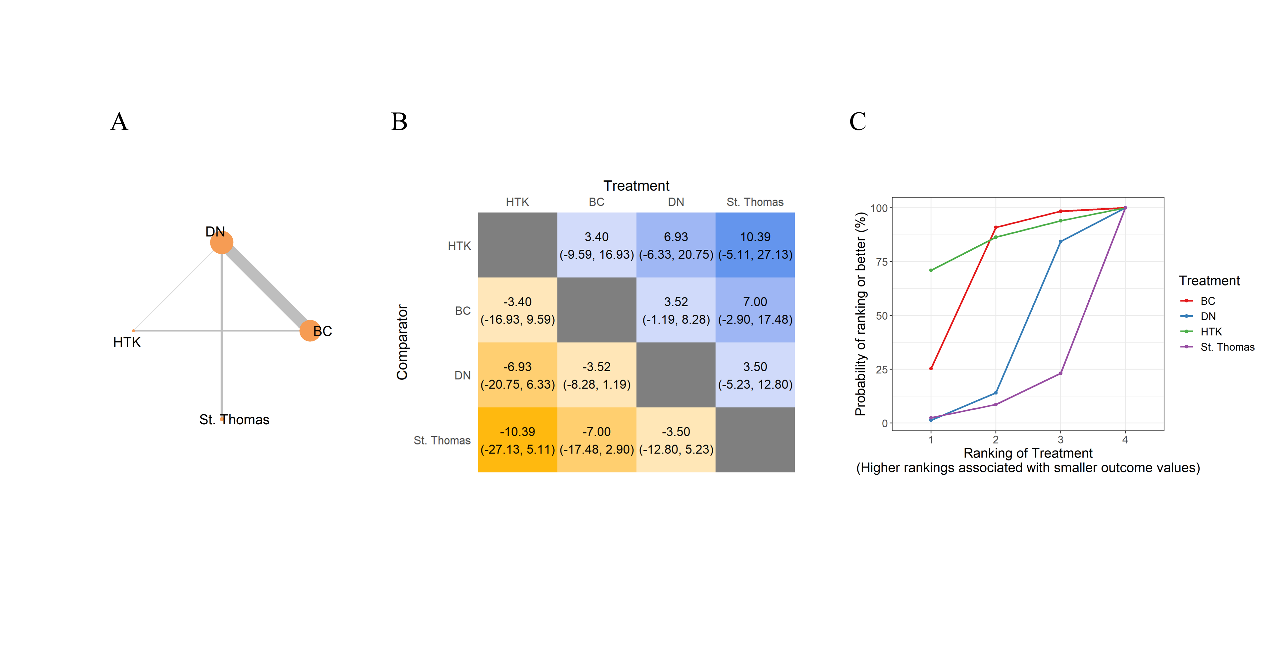


Figure S21. NMA of ICU stay across adult patients only in cohort studies.


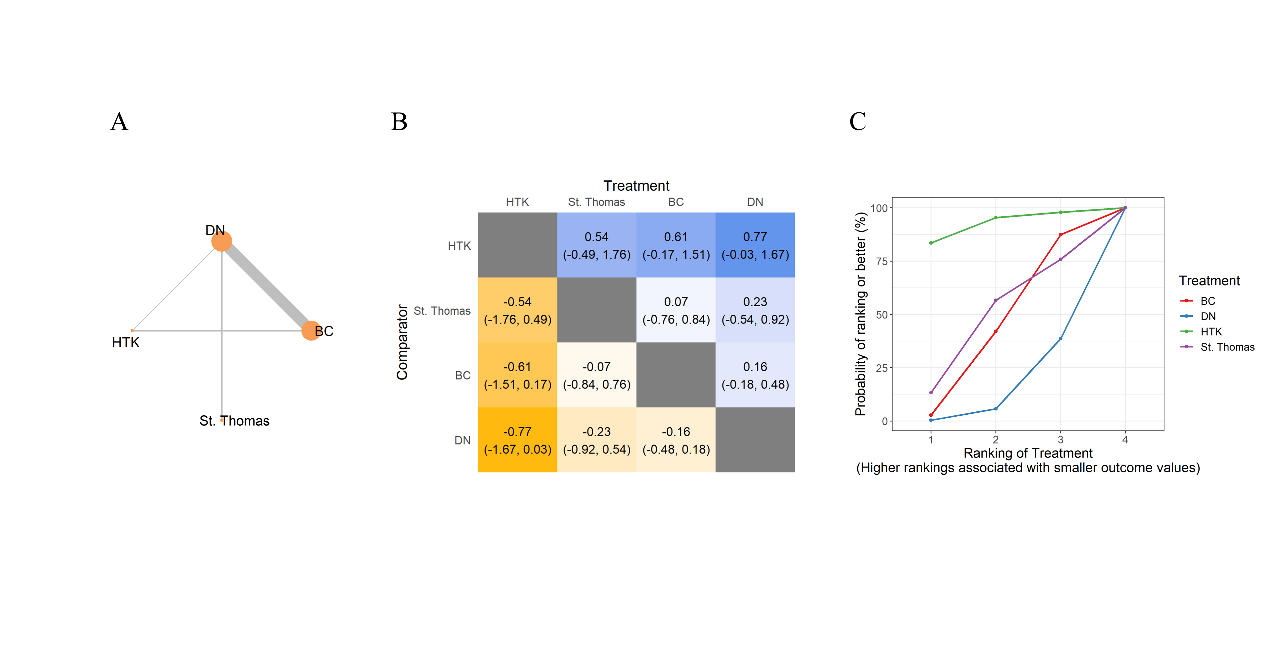


Figure S22. NMA of hospital stay across adult patients only in cohort studies.


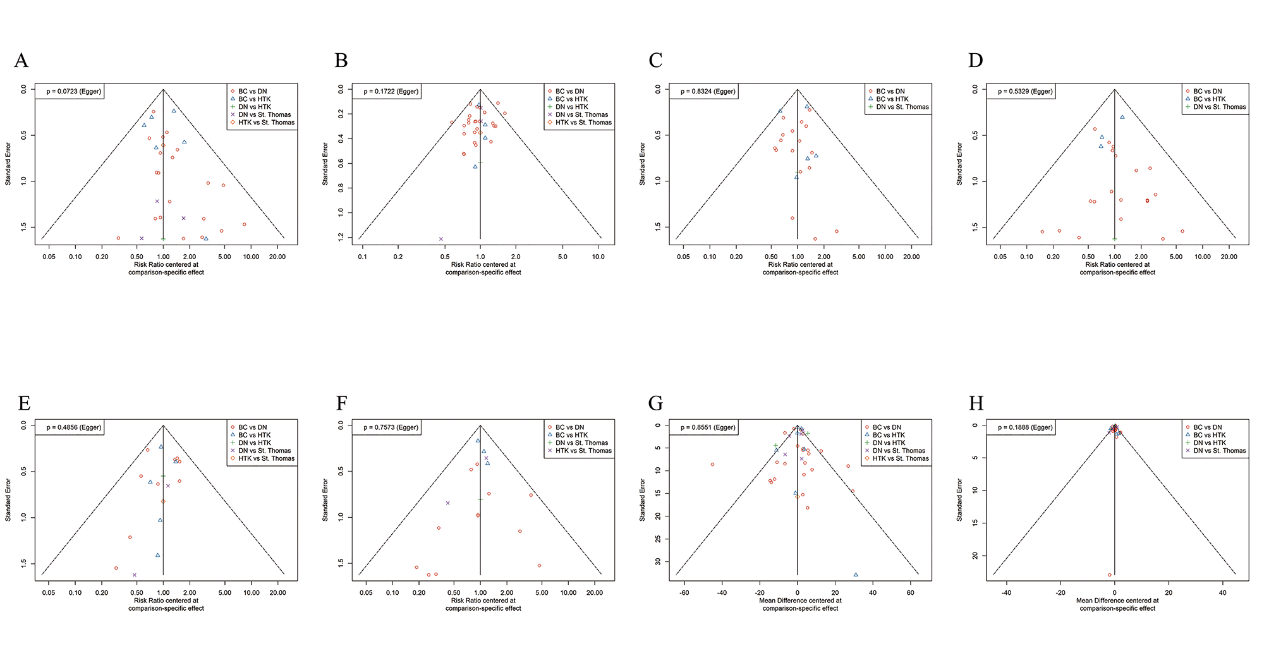


Figure S23. Comparison-adjusted funnel plots.


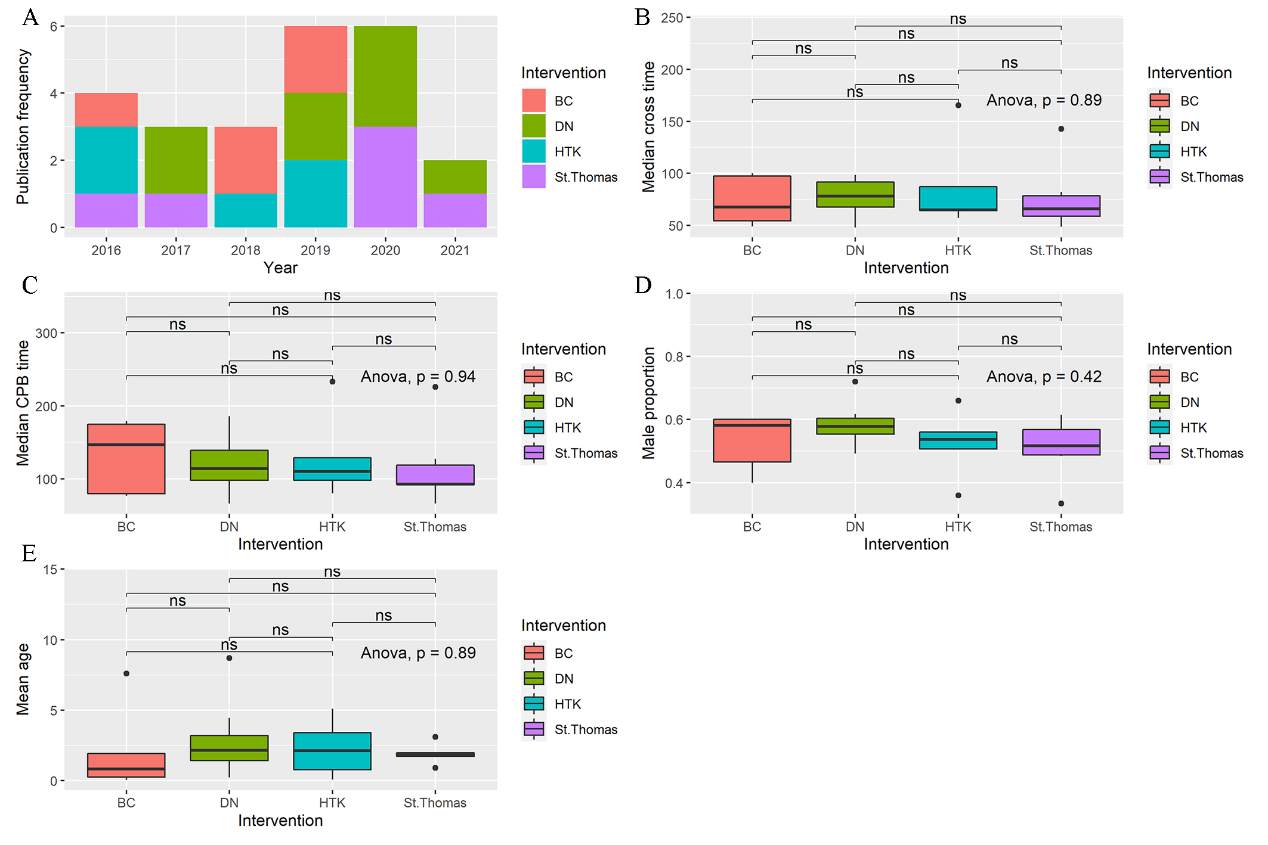


Figure S24. Transitivity analysis of each cardioplegia type across all pediatric patients.


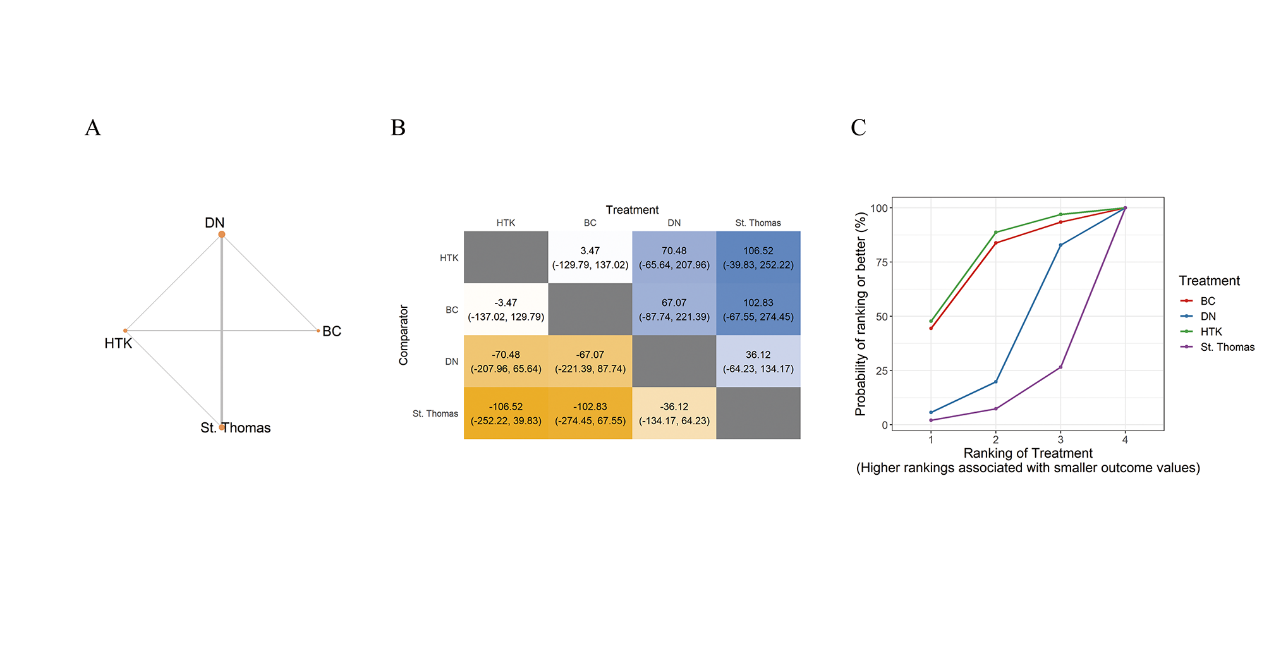


Figure S25. NMA of ICU stay across all pediatric trials.


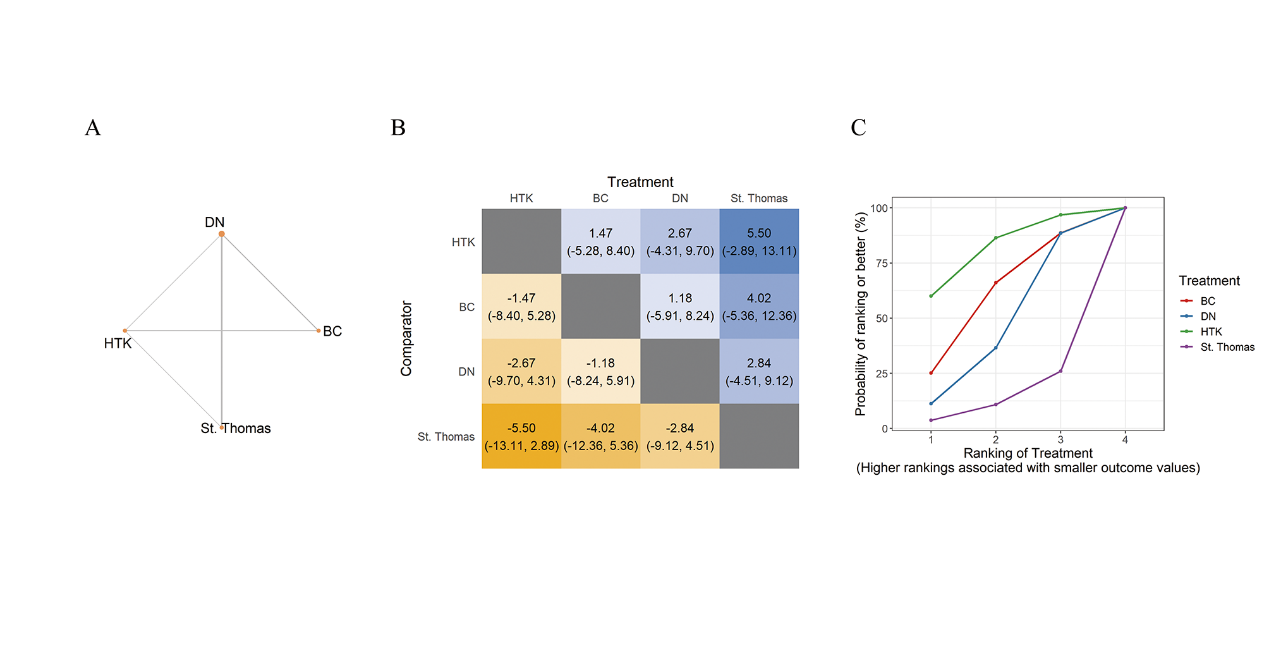


Figure S26. NMA of hospital stay across all pediatric trials.


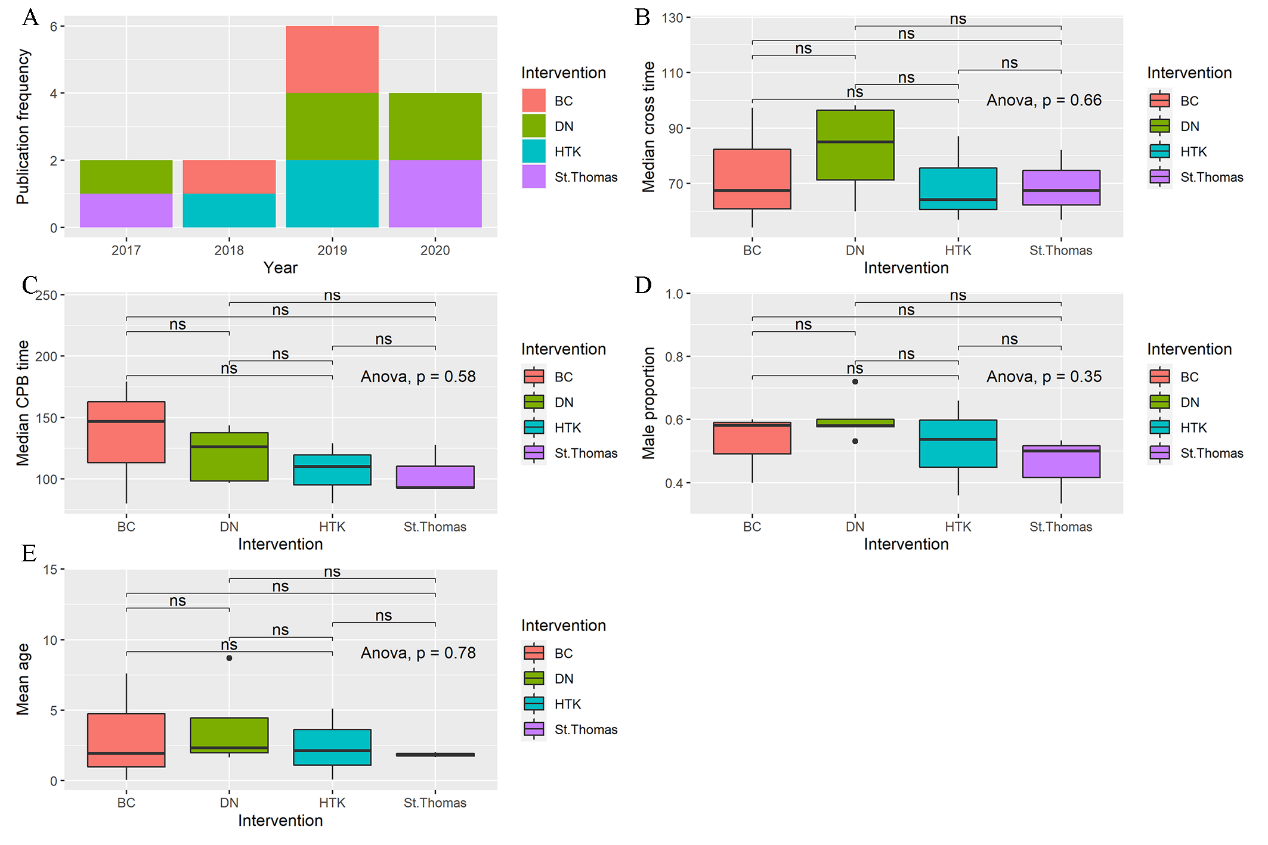


Figure S27. Transitivity analysis of each cardioplegia type across pediatric patients only in RCTs.


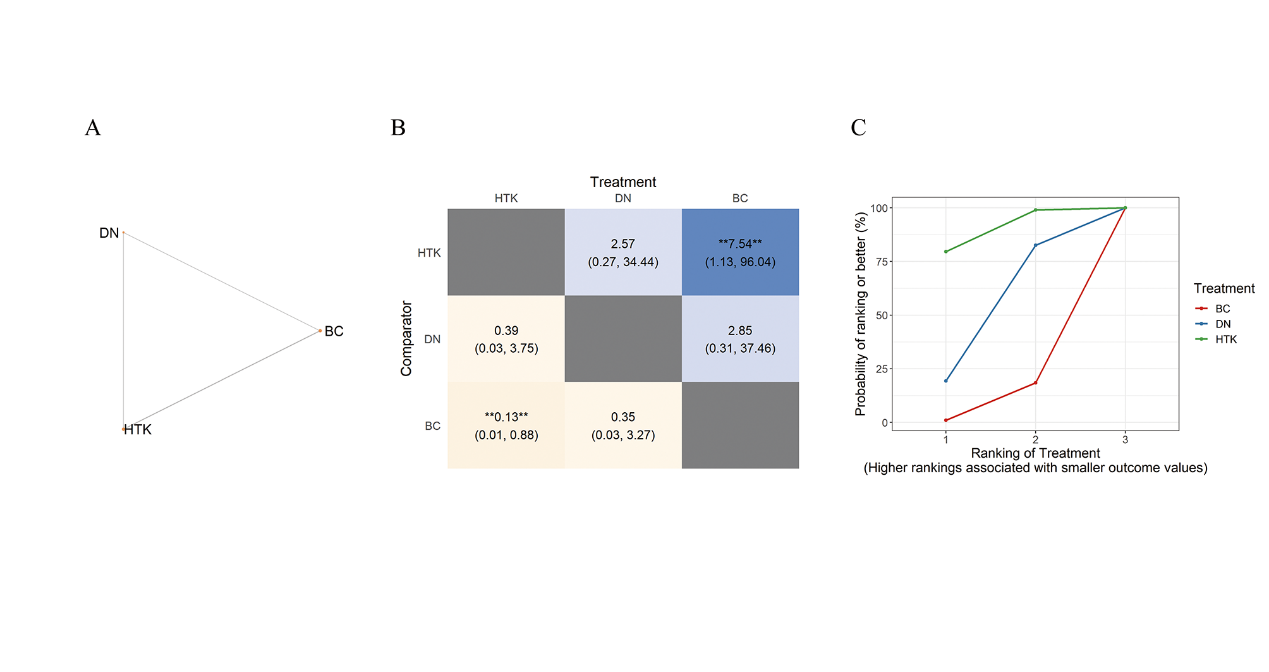


Figure S28. NMA of perioperative mortality across pediatric patients only in RCTs.


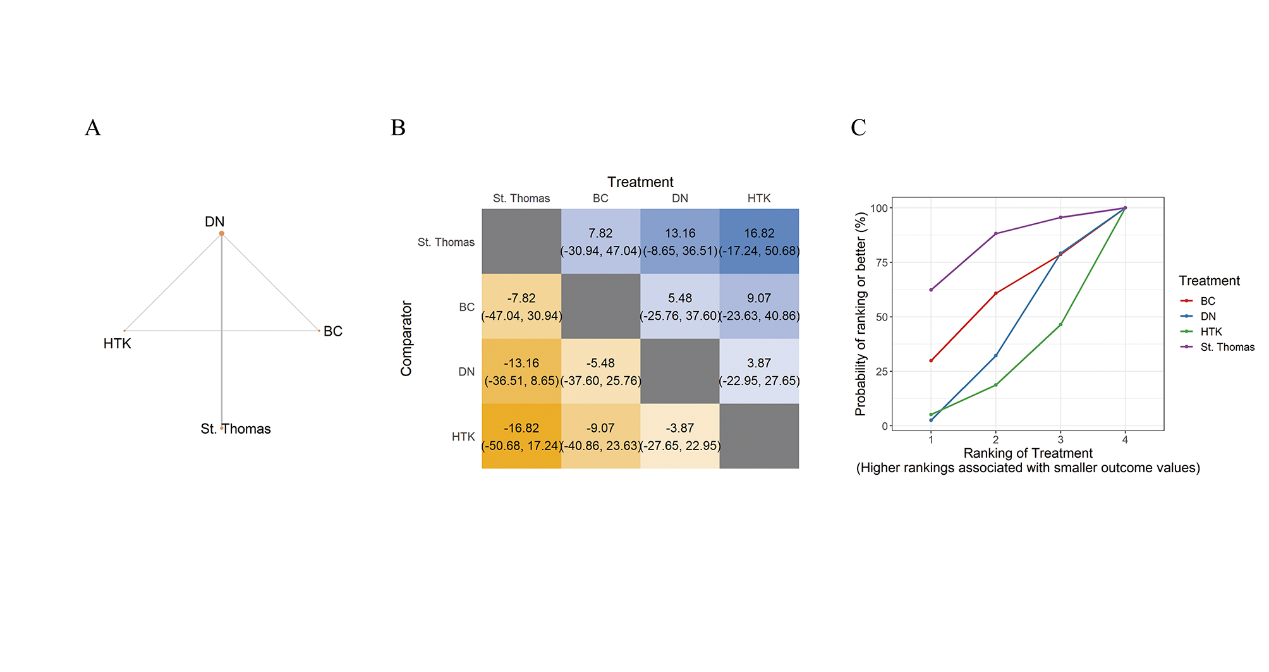


Figure S29. NMA of ICU stay across pediatric patients only in RCTs.


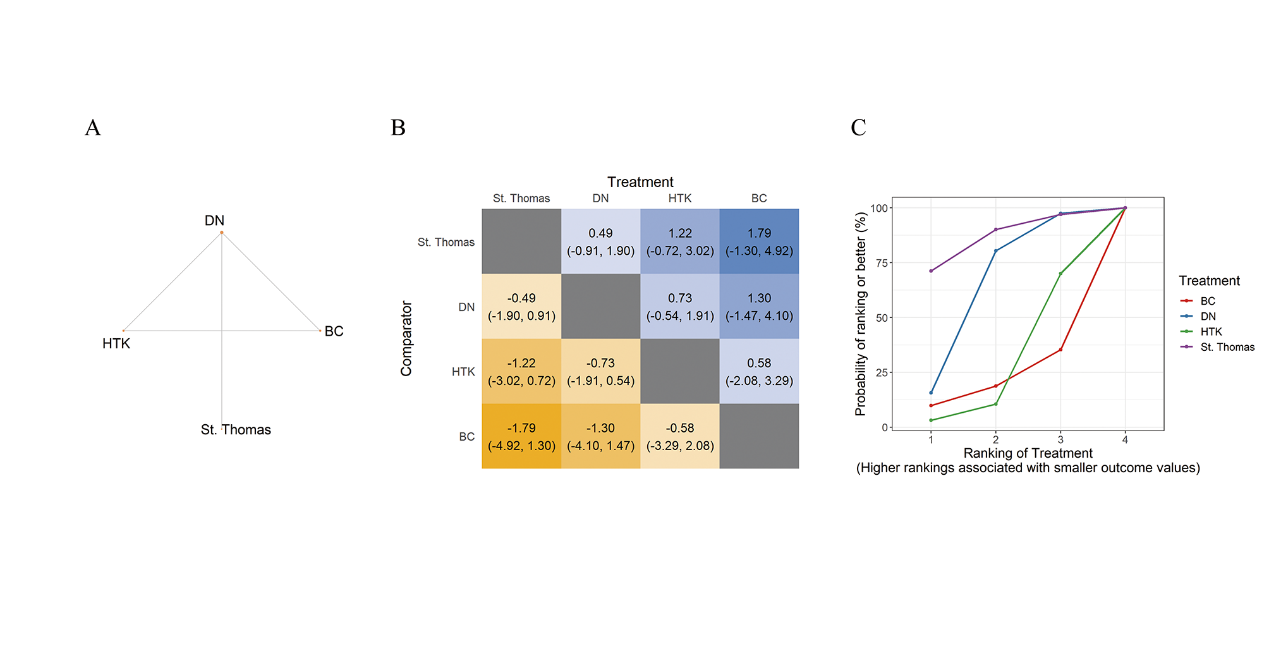


Figure S30. NMA of hospital stay across pediatric patients only in RCTs.


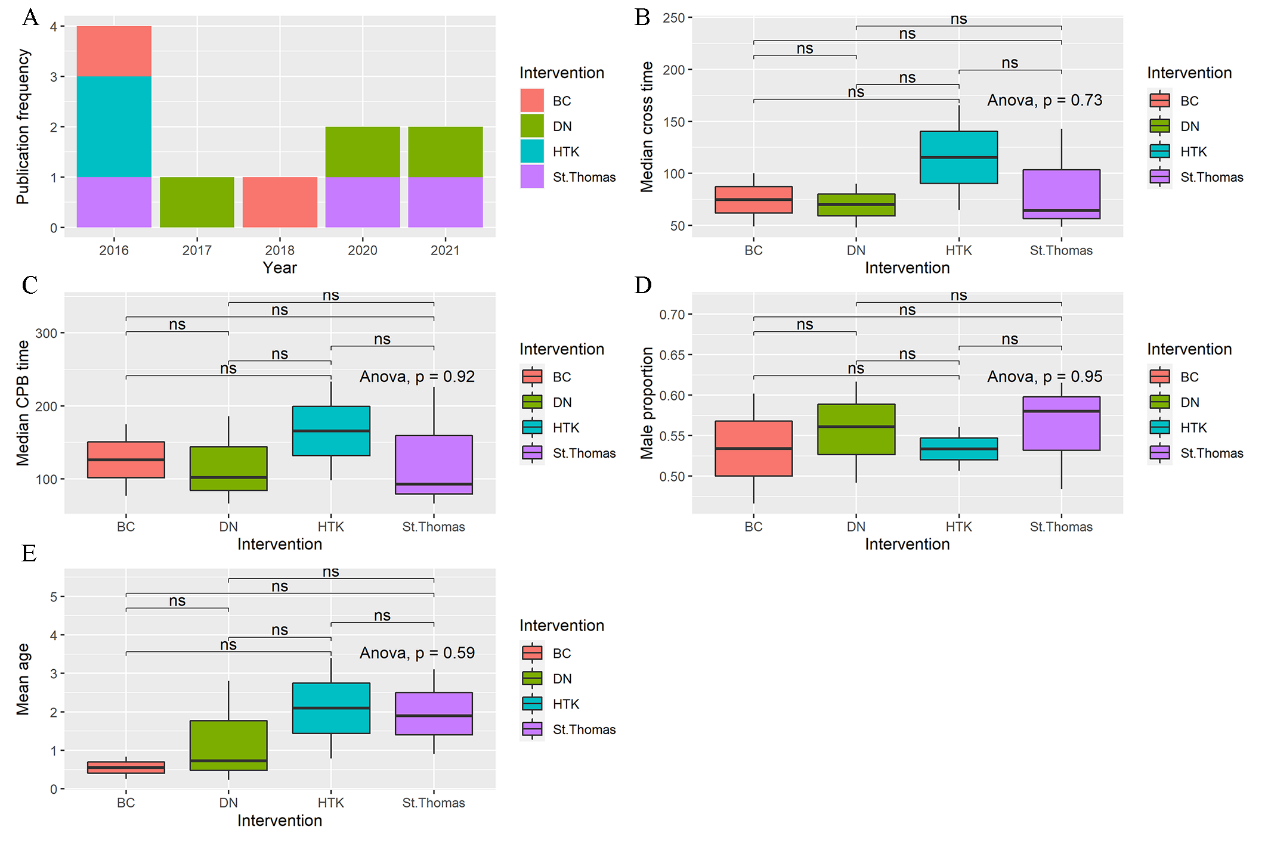


Figure S31. Transitivity analysis of each cardioplegia type across pediatric patients only in cohort studies.


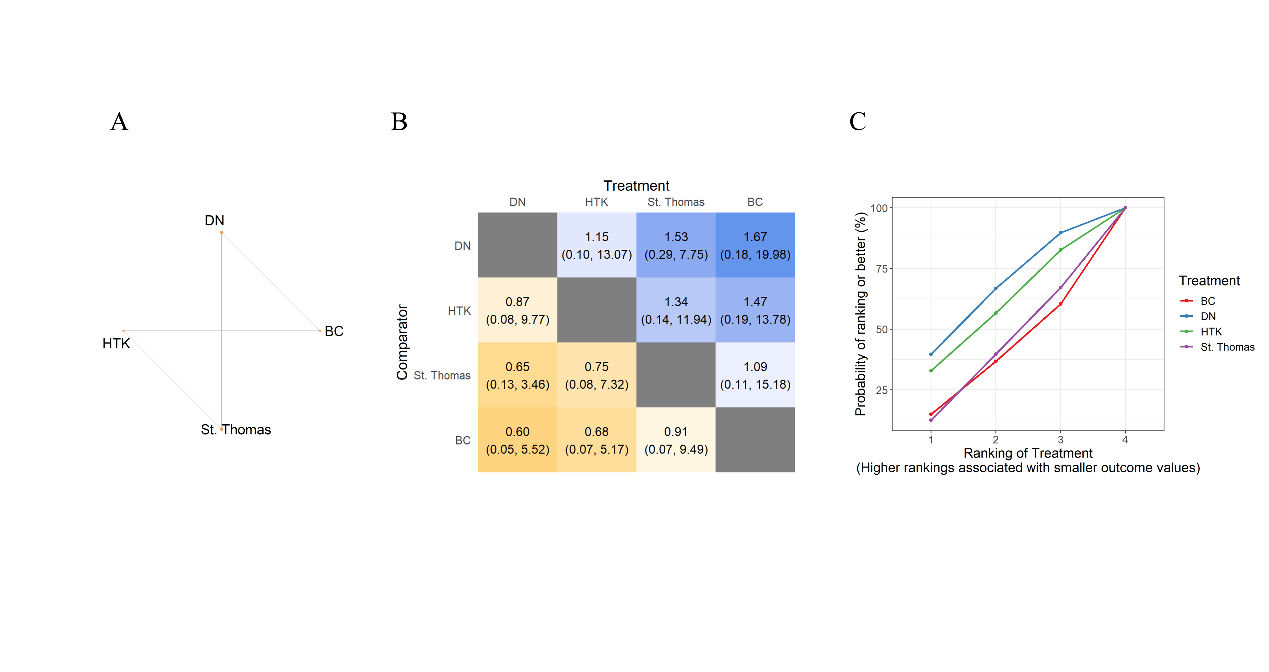


Figure S32. NMA of perioperative mortality across pediatric patients only in cohort studies.


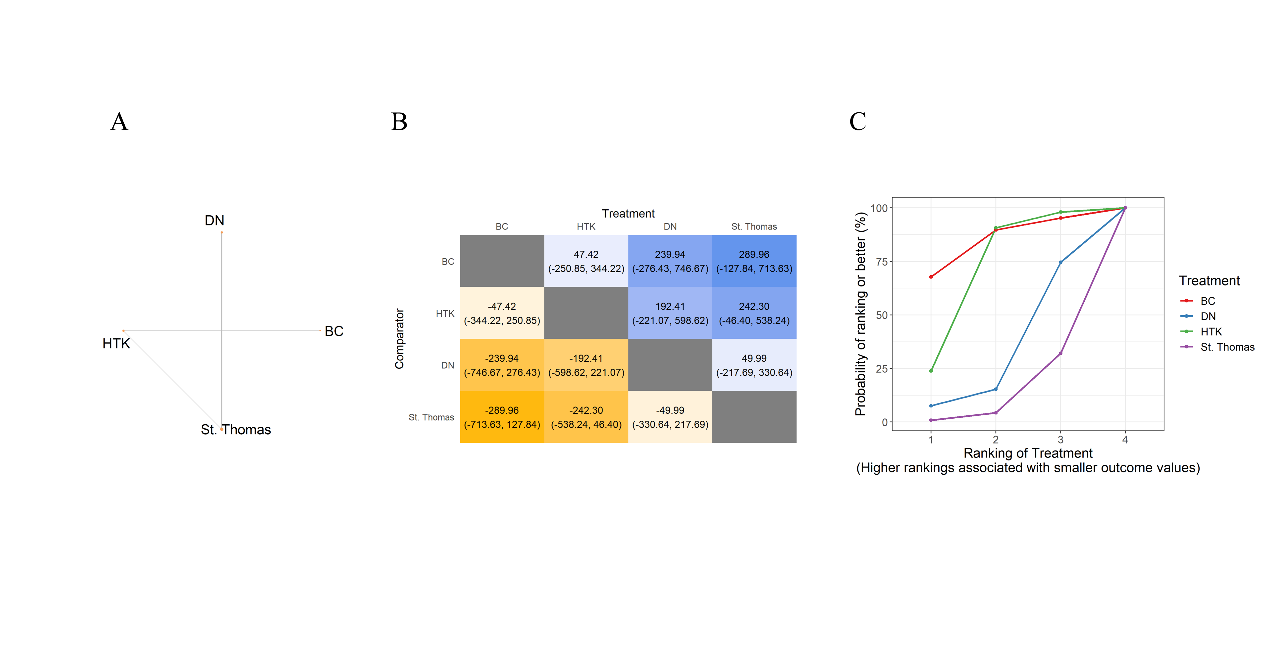


Figure S33. NMA of ICU stay across pediatric patients only in cohort studies.


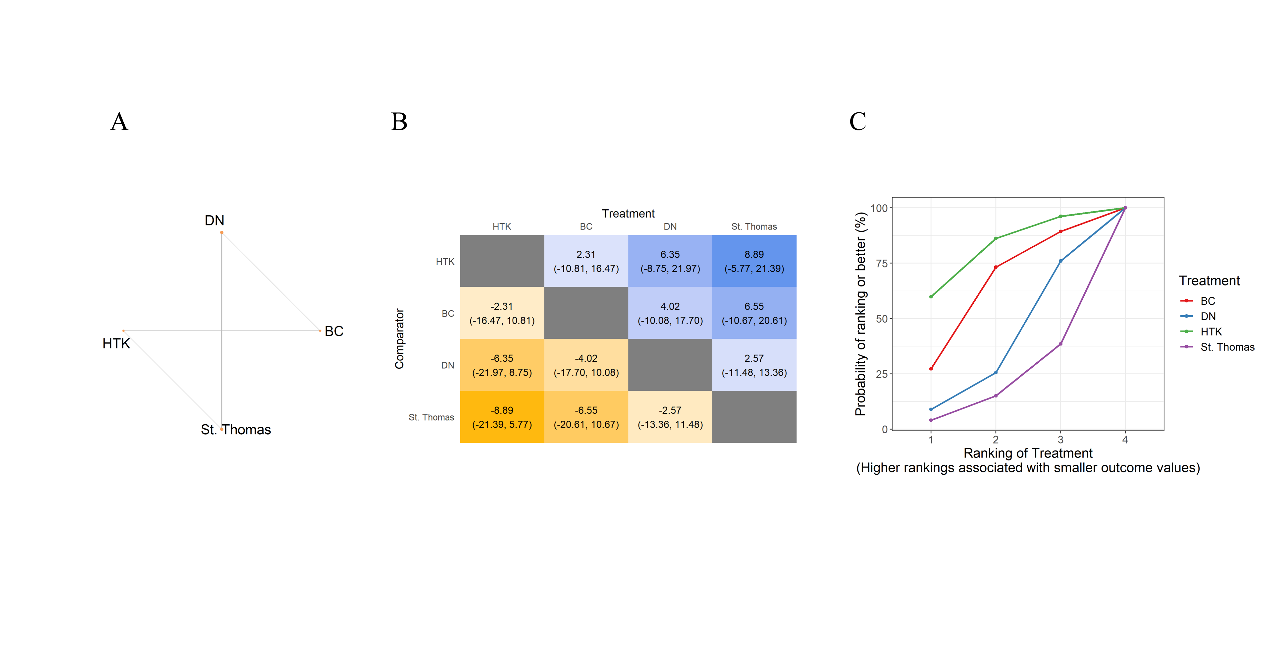


Figure S34. NMA of hospital stay across pediatric patients only in cohort studies.

Supplementary References

E1. Ad N, Holmes SD, Massimiano PS, Rongione AJ, Fornaresio LM, Fitzgerald D. The use of del Nido cardioplegia in adult cardiac surgery: A prospective randomized trial. *J Thorac Cardiovasc Surg*. (2018) 155:1011-18. doi: 10.1016/j.jtcvs.2017.09.146

E2. Ali I, Hassan A, Shokri H, Khorshed R. Efficacy of Histidine-Tryptophan-Ketoglutarate Solution Versus Blood Cardioplegia in Cardiac Surgical Procedures: A Randomized Controlled | Parallel Group Study. *Heart Surg Forum*. (2021) 24:E170-6. doi: 10.1532/hsf.3495

E3. Arafat AA, Hassan E, Alfonso JJ, Alanazi E, Alshammari AS, Mahmood A, Al-Otaibi K, Adam AI, Algarni KD, Pragliola C. Del Nido versus warm blood cardioplegia in adult patients with a low ejection fraction. *The Cardiothoracic Surgeon*. (2021) 29(1): 1-5. doi: 10.1186/s43057-021-00061-4

E4. Bigdelian H, Hosseini A. Effect of single-dose crystalloid cardioplegic agent compared to bloody cardioplegic agent in cardiac surgery in children with tetralogy of fallot. *ARYA atherosclerosis*. (2020) 16(1):24-32. doi: 10.22122/arya.v16i1.1943

E5. Braathen B, Jeppsson A, Scherstén H, Hagen OM, Vengen O, Rexius H, Lepore V, Tønnessen T. One single dose of histidine-tryptophan-ketoglutarate solution gives equally good myocardial protection in elective mitral valve surgery as repetitive cold blood cardioplegia: A prospective randomized study. *Journal of Thoracic and Cardiovascular Surgery*. (2011) 141:995-1001. doi: 10.1016/j.jtcvs.2010.07.011

E6. Busro PW, Romolo H, Sastroasmoro S, Rachmat J, Sadikin M, Santoso A, Boom CE, Suwarto S, Jusuf AA. Role of terminal warm blood cardioplegia in complex congenital heart surgery. *Asian Cardiovasc Thorac Ann*. (2018) 26:196-202. doi: 10.1177/0218492318759105

E7. Caneo LF, Matte GS, AL RT, Pegollo LMC, Amato Miglioli MC, G TdS, Amato LP, Miana LA, MR BM, Penha JG, et al. Initial experience with del Nido cardioplegia solution at a Pediatric and Congenital Cardiac Surgery Program in Brazil. *Perfusion*. (2021) 3:2676591211020471. doi: 10.1177/02676591211020471

E8. Cayir MC, Yuksel A. The Use of del Nido Cardioplegia for Myocardial Protection in Isolated Coronary Artery Bypass Surgery. *Heart Lung Circ*. (2020) 29:301-7. doi: 10.1016/j.hlc.2018.12.006

E9. Chen H, Wang L, Wan L, Xiao L, Chen X. Use of del Nido cardioplegia in acute aortic dissection surgery. *Perfusion*. (2021) 36:233-8. doi: 10.1177/0267659120938528

E10. Cvetkovic D, Kocica M, Soskic L, Vucicevic F, Petrovic O, Jovanovic I, Jovicic S, Trifkovic J, Kostovski S, Milicic B, et al. Comparison of Custodiol® and modified St. Thomas cardioplegia for myocardial protection in coronary artery bypass grafting. *Vojnosanitetski pregled*. (2020) 77:1126-34. doi: 10.2298/vsp181108192c

E11. Elassal AA, Al-Ebrahim K, Al-Radi O, Zaher ZF, Dohain AM, Abdelmohsen GA, Abdulla AH, Meshak MA, Abdulaziz MA, Eldesouki MS, et al. Myocardial Protection by Blood-Based Del Nido versus St. Thomas Cardioplegia in Cardiac Surgery for Adults and Children. *Heart Surg Forum*. (2020) 23:E689-95. doi: 10.1532/hsf.3099

E12. Gaudino M, Pragliola C, Anselmi A, Pieroni M, De Paulis S, Leone A, De Caterina AR, Massetti M. Randomized trial of HTK versus warm blood cardioplegia for right ventricular protection in mitral surgery. *Scandinavian Cardiovascular Journal*. (2013) 47:359-67. doi: 10.3109/14017431.2013.836241

E13. George G, Varsha AV, Philip MA, Vithayathil R, Srinivasan D, Sneha Princy FX, Sahajanandan R. Myocardial protection in cardiac surgery: Del Nido versus blood cardioplegia. *Ann Card Anaesth*. (2020) 23:477-84. doi: 10.4103/aca.ACA_153_19

E14. Gorjipour F, Dehaki MG, Totonchi Z, Hajimiresmaiel SJ, Azarfarin R, Pazoki-Toroudi H, Mahdavi M, Korbi M, Dehaki MG, Soltani B, et al. Inflammatory cytokine response and cardiac troponin i changes in cardiopulmonary bypass using two cardioplegia solutions; Del Nido and modified St. Thomas': A randomized controlled trial. *Perfusion (United Kingdom)*. (2017) 32:394-402. doi: 10.1177/0267659117691119

E15. Guajardo Salinas GE, Nutt R, Rodriguez-Araujo G. Del Nido cardioplegia in low risk adults undergoing first time coronary artery bypass surgery. *Perfusion*. (2017) 32:68-73. doi: 10.1177/0267659116661051

E16. Gunaydin S, Gunertem OE, Babaroglu S, Kunt AT, McCusker K, Ozisik K. Clinical outcomes of single-dose cardioplegia in high-risk coronary bypass. *Asian Cardiovasc Thorac Ann*. (2021) 29:77-83. doi: 10.1177/0218492320966434

E17. de Haan M, van Straten A, Overdevest E, de Jong M, Soliman-Hamad M. Safety of Custodiol cardioplegia: a cohort study in patients undergoing cardiac surgery with elongated aortic cross-clamp time. *Perfusion*. (2020) 35:591-7. doi: 10.1177/0267659119897239

E18. Hamad R, Nguyen A, Laliberté É, Bouchard D, Lamarche Y, El-Hamamsy I, Demers P. Comparison of del Nido Cardioplegia With Blood Cardioplegia in Adult Combined Surgery. *Innovations: Technology and Techniques in Cardiothoracic and Vascular Surgery*. (2017) 12:356-62. doi: 10.1097/IMI.0000000000000403

E19. Haranal M, Chin HC, Sivalingam S, Raja N, Mohammad Shaffie MS, Namasiwayam TK, Fadleen M, Fakhri N. Safety and Effectiveness of Del Nido Cardioplegia in Comparison to Blood-Based St. Thomas Cardioplegia in Congenital Heart Surgeries: A Prospective Randomized Controlled Study. *World J Pediatr Congenit Heart Surg*. (2020) 11:720-6. doi: 10.1177/2150135120936119

E20. Hoyer A, Lehmann S, Mende M, Noack T, Kiefer P, Misfeld M, Bakhtiary F, Mohr FW. Custodiol versus cold Calafiore for elective cardiac arrest in isolated aortic valve replacement: a propensity-matched analysis of 7263 patients. *Eur J Cardiothorac Surg*. (2017) 52:303-9. doi: 10.1093/ejcts/ezx052

E21. Brian W. Hummel M, Randall W. Buss, MD, Paul L. DiGiorgi, MD, Brittany N. Laviano, BSN. Myocardial Protection and Financial Considerations of Custodiol Cardioplegia in Minimally Invasive and Open Valve Surgery. *Innovations*. (2016) 11(6): 420-4. doi: 10.1097/IMI.0000000000000314

E22. Kuciński J, Górska A, Deja MA. Del Nido cardioplegia as a safe and effective method of myocardial protection in adult patients undergoing cardiac surgery: a single‑center experience. *Kardiol Pol*. (2019) 4:1040-6. doi: 10.33963/kp.14964

E23. Khaled D. Algarni MD M, FRCSC. Routine use of del Nido cardioplegia compared with blood cardioplegia in all types of adult cardiac surgery procedures. *J Card Surg*. (2020) 35:3340-6. doi: 10.1111/jocs.15060

E24. Kim WK, Kim HR, Kim JB, Jung SH, Choo SJ, Chung CH, Lee JW. del Nido cardioplegia in adult cardiac surgery: beyond single-valve surgery. *Interact Cardiovasc Thorac Surg*. (2018) 27:81-7. doi: 10.1093/icvts/ivy028

E25. Kim JS, Jeong JH, Moon SJ, Ahn H, Hwang HY. Sufficient myocardial protection of del Nido cardioplegia regardless of ventricular mass and myocardial ischemic time in adult cardiac surgical patients. *J Thorac Dis*. (2016) 8:2004-10. doi: 10.21037/jtd.2016.06.66

E26. Koda Y, Kitahara H, Nishida H, Jeevanandam V, Ota T. A proposed redosing interval of del Nido cardioplegia solution in adult cardiac surgery: a propensity-matched study. *Perfusion*. (2020) 36:463-9. doi: 10.1177/0267659120956534

E27. Koeckert MS, Smith DE, Vining PF, Ranganath NK, Beaulieu T, Loulmet DF, Zias E, Galloway AC, Grossi EA. Del Nido cardioplegia for minimally invasive aortic valve replacement. *Journal of Cardiac Surgery*. (2018) 33:64-8. doi: 10.1111/jocs.13536

E28. Kuserli Y, Turkyilmaz S, Turkyilmaz G, Kavala AA. Comparison of del Nido Cardioplegia and Blood Cardioplegia in Aortic Root Surgery. *Heart Surg Forum*. (2020) 23:E376-84. doi: 10.1532/hsf.2861

E29. Moktan Lama PB, Khakural P, Sigdel S, Raj Bhatta M, Sah Teli R, Baral RK, Bhattarai A, Pradhan B, Koirala B. Del Nido Cardioplegia in Coronary Artery Bypass Grafting Surgery: A safe, efficacious and economic alternative to St. Thomas solution; an experience from a developing nation. *Perfusion*. (2021) 36:470-5. doi: 10.1177/0267659121991033

E30. Lenoir M, Bouhout I, Jelassi A, Cartier R, Poirier N, El-Hamamsy I, Demers P. Del Nido cardioplegia versus blood cardioplegia in adult aortic root surgery. *J Thorac Cardiovasc Surg*. (2021) 162:514-22. doi: 10.1016/j.jtcvs.2020.01.022

E31. Li XW, Lin YZ, Lin H, Huang JB, Tang XM, Long XM, Lu WJ, Wen ZK, Liang J, Li DY, et al. Histidine-tryptophan-ketoglutarate solution decreases mortality and morbidity in high-risk patients with severe pulmonary arterial hypertension associated with complex congenital heart disease: an 11-year experience from a single institution. *Braz J Med Biol Res*. (2016) 49:e5208. doi: 10.1590/1414-431x20165208

E32. Luo H, Qi X, Shi H, Zhao H, Liu C, Chen H, Peng R, Yu Z, Hu K, Wang C, et al. Single-dose del Nido cardioplegia used in adult minimally invasive valve surgery. *J Thorac Dis*. (2019) 11:2373-82. doi: 10.21037/jtd.2019.05.78

E33. Marzouk M, Lafreniere-Bessi V, Dionne S, Simard S, Pigeon C, Dagenais F, Ad N, Jacques F. Transitioning to Del Nido cardioplegia for all-comers: the next switching gear? *BMC Cardiovasc Disord*. (2020) 20(1): 1-8. doi: 10.1186/s12872-020-01506-0

E34. Mehrabanian MJ, Dehghani Firoozabadi M, Ahmadi Tafti SH, Forouzan Nia SK, Najafi A, Mortazian M, Zeraatian Nejad Davani S, Soltaninia H, Ghiasi A, Gorjipour F, et al. Clinical Outcomes and Electrolyte Balance Factors in Complex Cardiac Operations in Adults; Del Nido® Versus Custodiol® Cardioplegia Solutions: A Randomized Controlled Clinical Trial. *Iranian Red Crescent Medical Journal*. (2018) 20(4). doi: 10.5812/ircmj.64648

E35. Mercan I, Dereli Y, Topcu C, Tanyeli O, Isik M, Gormus N, Ozturk EY. Comparison between the Effects of Bretschneider's HTK Solution and Cold Blood Cardioplegia on Systemic Endothelial Functions in Patients who Undergo Coronary Artery Bypass Surgery: a Prospective Randomized and Controlled Trial. *Braz J Cardiovasc Surg*. (2020) 35:634-43. doi: 10.21470/1678-9741-2019-0327

E36. Mick SL, Robich MP, Houghtaling PL, Gillinov AM, Soltesz EG, Johnston DR, Blackstone EH, Sabik JF, 3rd. del Nido versus Buckberg cardioplegia in adult isolated valve surgery. *J Thorac Cardiovasc Surg*. (2015) 149:626-34. doi: 10.1016/j.jtcvs.2014.10.085

E37. Mishra P, Jadhav RB, Mohapatra CK, Khandekar J, Raut C, Ammannaya GK, Seth HS, Singh J, Shah V. Comparison of del Nido cardioplegia and St. Thomas Hospital solution - two types of cardioplegia in adult cardiac surgery. *Kardiochir Torakochirurgia Pol*. (2016) 13:295-9. doi: 10.5114/kitp.2016.64867

E38. Negi SL, Mandal B, Singh RS, Puri GD. Myocardial protection and clinical outcomes in Tetralogy of Fallot patients undergoing intracardiac repair: a randomized study of two cardioplegic techniques. *Perfusion (United Kingdom)*. (2019) 34:495-502. doi: 10.1177/0267659119828890

E39. O'Donnell C, Wang H, Tran P, Miller S, Shuttleworth P, Boyd JH. Utilization of Del Nido Cardioplegia in Adult Coronary Artery Bypass Grafting　- A Retrospective Analysis. *Circ J*. (2019) 83:342-6. doi: 10.1253/circj.CJ-18-0780

E40. Orak Y, Kocarslan A, Boran OF, Acıpayam M, Eroglu E, Kirisci M, Doganer A. Comparison of the operative and postoperative effects of del Nido and blood cardioplegia solutions in cardiopulmonary bypass surgery. *Braz J Cardiovasc Surg*. (2020) 35:689-96. doi: 10.21470/1678-9741-2019-0436

E41. Ota T, Yerebakan H, Neely RC, Mongero L, George I, Takayama H, Williams MR, Naka Y, Argenziano M, Bacha E, et al. Short-term outcomes in adult cardiac surgery in the use of del Nido cardioplegia solution. *Perfusion*. (2016) 31:27-33. doi: 10.1177/0267659115599453

E42. De Palo M, Guida P, Mastro F, Nanna D, Quagliara TA, Rociola R, Lionetti G, Paparella D. Myocardial protection during minimally invasive cardiac surgery through right mini-thoracotomy. *Perfusion*. (2017) 32:245-52. doi: 10.1177/0267659116679249

E43. Pizano A, Montes FR, Carreño M, Echeverri D, Umaña JP. Histidine-Tryptophan-Ketoglutarate Solution versus Blood Cardioplegia in Cardiac Surgery: A Propensity-Score Matched Analysis. *Heart Surg Forum*. (2018) 21:E158-64. doi: 10.1532/hsf.1920

E44. Pourmoghadam KK, Ruzmetov M, O'Brien MC, Piggott KD, Plancher G, Narasimhulu SS, Benjamin T, Decampli WM. Comparing del Nido and Conventional Cardioplegia in Infants and Neonates in Congenital Heart Surgery. *Ann Thorac Surg*. (2017) 103:1550-6. doi: 10.1016/j.athoracsur.2016.10.070

E45. Pragliola C, Hassan E, Ismail H, Al Otaibi K, Alfonso JJ, Algarni KD. del Nido Cardioplegia in Adult Patients: A Propensity-Matched Study of 102 Consecutive Patients. *Heart Lung Circ*. (2020) 29:1405-11. doi: 10.1016/j.hlc.2019.08.019

E46. Qulisy EA, Fakiha A, Debis RS, Jamjoom AA, Elassal AA, Al-Radi OO. Custodiol versus blood cardioplegia in pediatric cardiac surgery, two-center study. *Journal of the Egyptian Society of Cardio-Thoracic Surgery*. (2016) 24:38-42. doi: 10.1016/j.jescts.2016.05.001

E47. Rai G, Chandrababu R, Sevagur Kamath G, Pai B S. Analogy of Del Nido and conventional cardioplegia in patients undergoing isolated mitral valve replacement surgery: A single-center retrospective study. *Clinical Epidemiology and Global Health*. (2021) 12:100829. doi: 10.1016/j.cegh.2021.100829

E48. Reidy MR, Jimenez E, Omer S, Cornwell LD, Runbeck SX, Preventza O, Loor G, Rosengart TK, Coselli JS. Single-Dose del Nido Cardioplegia Compared With Standard Cardioplegia During Coronary Artery Bypass Grafting at a Veterans Affairs Hospital. *Tex Heart Inst J*. (2021) 48(1): e196981. doi: 10.14503/thij-19-6981

E49. Ross JDW, Newland RF, Hamson RTJ, Rice GD, Baker RA. Del Nido cardioplegia in adult cardiac surgery: analysis of myocardial protection and post-operative high-sensitivity Troponin T. *ANZ J Surg*. (2021) 91:2192-8. doi: 10.1111/ans.17135

E50. Sanetra K, Gerber W, Shrestha R, Domaradzki W, Krzych L, Zembala M, Cisowski M. The del Nido versus cold blood cardioplegia in aortic valve replacement: A randomized trial. *J Thorac Cardiovasc Surg*. (2020) 159:2275-83. doi: 10.1016/j.jtcvs.2019.05.083

E51. Sanri US, Ozsin KK, Toktas F, Yavuz S. Comparison of Del Nido Cardioplegia and Blood Cardioplegia in Terms of Development of Postoperative Atrial Fibrillation in Patients Undergoing Isolated Coronary Artery Bypass Grafting. *Braz J Cardiovasc Surg*. (2021) 36:158-64. doi: 10.21470/1678-9741-2020-0047

E52. Schutz A, Zhang Q, Bertapelle K, et al. Del Nido cardioplegia in coronary surgery: a propensity-matched analysis[J]. Interactive CardioVascular and Thoracic Surgery, (2020) 30(5): 699-705. doi: 10.1093/icvts/ivaa010

E53. Sharma A, Dixit S, Mittal S, Sharma M, Sharma D, Mawar KK. DelNido cardioplegia versus St Thomas cardioplegia solution in double valve replacement: a single centre experience. *Perfusion*. (2021) 36:476-81. doi: 10.1177/0267659120961921

E54. Shi H, Luo H, Qi X, Zhao H, Liu C, Chen H, Peng R, Yu Z, Hu K, Li X, et al. del Nido cardioplegia in surgery for aortic root disease: a historically controlled study. *J Thorac Dis*. (2020) 12:4105-14. doi: 10.21037/jtd-20-1101

E55. Shu C, Hong L, Shen X, Zhang W, Niu Y, Song X, Kong J, Zhang C. Effect of Del Nido cardioplegia on ventricular arrhythmias after cardiovascular surgery. *BMC Cardiovasc Disord*. (2021) 21(1): 1-11. doi: 10.1186/s12872-020-01844-z

E56. Talwar S, Chatterjee S, Sreenivas V, Makhija N, Kapoor PM, Choudhary SK, Airan B. Comparison of del Nido and histidine-tryptophan-ketoglutarate cardioplegia solutions in pediatric patients undergoing open heart surgery: A prospective randomized clinical trial. *J Thorac Cardiovasc Surg*. (2019) 157:1182-92. doi: 10.1016/j.jtcvs.2018.09.140

E57. Timek T, Willekes C, Hulme O, Himelhoch B, Nadeau D, Borgman A, Clousing J, Kanten D, Wagner J. Propensity Matched Analysis of del Nido Cardioplegia in Adult Coronary Artery Bypass Grafting: Initial Experience With 100 Consecutive Patients. *Ann Thorac Surg*. (2016) 101:2237-41. doi: 10.1016/j.athoracsur.2015.12.058

E58. Timek TA, Beute T, Robinson JA, Zalizadeh D, Mater R, Parker JL, Lypka M, Willekes CL. Del Nido cardioplegia in isolated adult coronary artery bypass surgery. *J Thorac Cardiovasc Surg*. (2020) 160:1479-85. doi: 10.1016/j.jtcvs.2019.09.027

E59. Ucak HA, Uncu H. Comparison of Del Nido and Intermittent Warm Blood Cardioplegia in Coronary Artery Bypass Grafting Surgery. *Ann Thorac Cardiovasc Surg*. (2019) 25:39-45. doi: 10.5761/atcs.oa.18-00087

E60. Ucak HA, Ucak D. Single-Dose Del Nido Cardioplegia vs. Blood Cardioplegia in Aortic Valve Replacement Surgery. *Braz J Cardiovasc Surg*. (2021) 36:229-36. doi: 10.21470/1678-9741-2020-0063

E61. Vaidya S, Adhikari AB, Karmacharya R, Rai K. Comparative study between the use of St. Thomas' II cardioplegia and del Nido cardioplegia in patients who underwent open-heart surgery. *Nepalese Heart Journal*. (2020) 17:23-7. doi: 10.3126/njh.v17i1.28802

E62. Valente AS, Lustosa GP, Mota LAM, Lima A, Mesquita FA, Gondim A, Rodrigues FA, Pompeu RG, Branco KC. Comparative Analysis of Myocardial Protection with HTK Solution and Hypothermic Hyperkalemic Blood Solution in the Correction of Acyanogenic Congenital Cardiopathies - A Randomized Study. *Brazilian journal of cardiovascular surgery*. (2019) 34:271‐8. doi: 10.21470/1678-9741-2018-0243

E63. Viana FF, Shi WY, Hayward PA, Larobina ME, Liskaser F, Matalanis G. Custodiol versus blood cardioplegia in complex cardiac operations: An Australian experience. *European Journal of Cardio-thoracic Surgery*. (2013) 43:526-31. doi: 10.1093/ejcts/ezs319

E64. Vistarini N, Laliberte E, Beauchamp P, Bouhout I, Lamarche Y, Cartier R, Carrier M, Perrault L, Bouchard D, El-Hamamsy I, et al. Del Nido cardioplegia in the setting of minimally invasive aortic valve surgery. *Perfusion*. (2017) 32:112-7. doi: 10.1177/0267659116662701

E65. Vivacqua A, Robinson J, Abbas AE, Altshuler JM, Shannon FL, Podolsky RH, Sakwa MP. Single-dose cardioplegia protects myocardium as well as traditional repetitive dosing: A noninferiority randomized study. *J Thorac Cardiovasc Surg*. (2020) 159:1857-63. doi: 10.1016/j.jtcvs.2019.03.125

E66. Halit Yerebakan RAS, Marc Najjar, Estibaliz Castillero, Linda Mongero. Del Nido Cardioplegia can be safely administered in high-risk coronary artery bypass grafting surgery after acute myocardial infarction: a propensity matched comparison. (2014) 9(1): 1-7. doi: 10.1186/s13019-014-0141-5

E67. Ziazadeh D, Mater R, Himelhoch B, Borgman A, Parker JL, Willekes CL, Timek TA. Single-dose del Nido Cardioplegia in Minimally Invasive Aortic Valve Surgery. *Semin Thorac Cardiovasc Surg*. (2017) 29(4): 471-6. doi: 10.1053/j.semtcvs.2017.10.001

PRISMA NMA Checklist of Items to Include When Reporting A Systematic Review Involving a Network Meta-analysis

| Section/Topic | Item # | Checklist Item | Reported on Page # |
| --- | --- | --- | --- |
| TITLE |  |  |  |
| Title | 1 | Identify the report as a systematic review *incorporating a network meta-analysis (or related form of meta-analysis).* | *1* |
|  |  |  |  |
| ABSTRACT |  |  | *2* |
| Structured summary | 2 | Provide a structured summary including, as applicable:  Background: main objectives  Methods: data sources; study eligibility criteria, participants, and interventions; study appraisal; and *synthesis methods, such as network meta-analysis.*  Results: number of studies and participants identified; summary estimates with corresponding confidence/credible intervals; *treatment rankings may also be discussed. Authors may choose to summarize pairwise comparisons against a chosen treatment included in their analyses for brevity.*  Discussion/Conclusions: limitations; conclusions and implications of findings.  Other: primary source of funding; systematic review registration number with registry name. |  |
|  |  |  |  |
| INTRODUCTION |  |  |  |
| Rationale | 3 | Describe the rationale for the review in the context of what is already known*, including mention of why a network meta-analysis has been conducted.* | *3* |
| Objectives | 4 | Provide an explicit statement of questions being addressed, with reference to participants, interventions, comparisons, outcomes, and study design (PICOS). | *3* |
|  |  |  |  |
| METHODS |  |  |  |
| Protocol and registration | 5 | Indicate whether a review protocol exists and if and where it can be accessed (e.g., Web address); and, if available, provide registration information, including registration number. | */* |
| Eligibility criteria | 6 | Specify study characteristics (e.g., PICOS, length of follow-up) and report characteristics (e.g., years considered, language, publication status) used as criteria for eligibility, giving rationale. *Clearly describe eligible treatments included in the treatment network, and note whether any have been clustered or merged into the same node (with justification).* | *4* |
| Information sources | 7 | Describe all information sources (e.g., databases with dates of coverage, contact with study authors to identify additional studies) in the search and date last searched. | *4* |
| Search | 8 | Present full electronic search strategy for at least one database, including any limits used, such that it could be repeated. | *4* |
| Study selection | 9 | State the process for selecting studies (i.e., screening, eligibility, included in systematic review, and, if applicable, included in the meta-analysis). | *4* |
| Data collection process | 10 | Describe method of data extraction from reports (e.g., piloted forms, independently, in duplicate) and any processes for obtaining and confirming data from investigators. | *4* |
| Data items | 11 | List and define all variables for which data were sought (e.g., PICOS, funding sources) and any assumptions and simplifications made. | *4* |
| Geometry of the network | S1 | Describe methods used to explore the geometry of the treatment network under study and potential biases related to it. This should include how the evidence base has been graphically summarized for presentation, and what characteristics were compiled and used to describe the evidence base to readers. | *5* |
| Risk of bias within individual studies | 12 | Describe methods used for assessing risk of bias of individual studies (including specification of whether this was done at the study or outcome level), and how this information is to be used in any data synthesis. | *4-5* |
| Summary measures | 13 | State the principal summary measures (e.g., risk ratio, difference in means). *Also describe the use of additional summary measures assessed, such as treatment rankings and surface under the cumulative ranking curve (SUCRA) values, as well as modified approaches used to present summary findings from meta-analyses.* | *5* |
| Planned methods of analysis | 14 | Describe the methods of handling data and combining results of studies for each network meta-analysis. This should include, but not be limited to:   - *Handling of multi-arm trials;* - *Selection of variance structure;* - *Selection of prior distributions in Bayesian analyses; and* - *Assessment of model fit.* | *5* |
| Assessment of Inconsistency | S2 | Describe the statistical methods used to evaluate the agreement of direct and indirect evidence in the treatment network(s) studied. Describe efforts taken to address its presence when found. | *5* |
| Risk of bias across studies | 15 | Specify any assessment of risk of bias that may affect the cumulative evidence (e.g., publication bias, selective reporting within studies). | *5* |
| Additional analyses | 16 | Describe methods of additional analyses if done, indicating which were pre-specified. This may include, but not be limited to, the following:   - Sensitivity or subgroup analyses; - Meta-regression analyses; - *Alternative formulations of the treatment network; and* - *Use of alternative prior distributions for Bayesian analyses (if applicable).* | *5* |
|  |  |  |  |
| RESULTS |  |  |  |
| Study selection | 17 | Give numbers of studies screened, assessed for eligibility, and included in the review, with reasons for exclusions at each stage, ideally with a flow diagram. | *6，Figure 1* |
| Presentation of network structure | S3 | Provide a network graph of the included studies to enable visualization of the geometry of the treatment network. | [*Supplementary materials*](javascript:;) |
| Summary of network geometry | S4 | Provide a brief overview of characteristics of the treatment network. This may include commentary on the abundance of trials and randomized patients for the different interventions and pairwise comparisons in the network, gaps of evidence in the treatment network, and potential biases reflected by the network structure. | *9-10* |
| Study characteristics | 18 | For each study, present characteristics for which data were extracted (e.g., study size, PICOS, follow-up period) and provide the citations. | *6,7,* [*Supplementary materials*](javascript:;) |
| Risk of bias within studies | 19 | Present data on risk of bias of each study and, if available, any outcome level assessment. | [*Supplementary materials*](javascript:;) |
| Results of individual studies | 20 | For all outcomes considered (benefits or harms), present, for each study: 1) simple summary data for each intervention group, and 2) effect estimates and confidence intervals. *Modified approaches may be needed to deal with information from larger networks.* | *6-8* |
| Synthesis of results | 21 | Present results of each meta-analysis done, including confidence/credible intervals. *In larger networks, authors may focus on comparisons versus a particular comparator (e.g. placebo or standard care), with full findings presented in an appendix. League tables and forest plots may be considered to summarize pairwise comparisons.* If additional summary measures were explored (such as treatment rankings), these should also be presented. | *6-8* |
| Exploration for inconsistency | S5 | Describe results from investigations of inconsistency. This may include such information as measures of model fit to compare consistency and inconsistency models, *P* values from statistical tests, or summary of inconsistency estimates from different parts of the treatment network. | *7,8* |
| Risk of bias across studies | 22 | Present results of any assessment of risk of bias across studies for the evidence base being studied. | *8* |
| Results of additional analyses | 23 | Give results of additional analyses, if done (e.g., sensitivity or subgroup analyses, meta-regression analyses*, alternative network geometries studied, alternative choice of prior distributions for Bayesian analyses,* and so forth). | *8,9* |
|  |  |  |  |
| DISCUSSION |  |  |  |
| Summary of evidence | 24 | Summarize the main findings, including the strength of evidence for each main outcome; consider their relevance to key groups (e.g., healthcare providers, users, and policy-makers). | *9-10* |
| Limitations | 25 | Discuss limitations at study and outcome level (e.g., risk of bias), and at review level (e.g., incomplete retrieval of identified research, reporting bias). *Comment on the validity of the assumptions, such as transitivity and consistency. Comment on any concerns regarding network geometry (e.g., avoidance of certain comparisons).* | *10-11* |
| Conclusions | 26 | Provide a general interpretation of the results in the context of other evidence, and implications for future research. | *11* |
|  |  |  |  |
| FUNDING |  |  |  |
| Funding | 27 | Describe sources of funding for the systematic review and other support (e.g., supply of data); role of funders for the systematic review. This should also include information regarding whether funding has been received from manufacturers of treatments in the network and/or whether some of the authors are content experts with professional conflicts of interest that could affect use of treatments in the network. | *11* |
